# Aluminium ammonium sulphate induces epithelial pyroptosis and eosinophilic inflammation under dysbiotic conditions: ameliorative effects of heparin

**DOI:** 10.1038/s41420-026-03237-1

**Published:** 2026-07-01

**Authors:** Ayako Wakabayashi, Atsuko Owaki, Ken Iwatsuki, Etsuko Toda, Keisuke Tanaka, Soichiro Kumamoto, Yuichi Koshiishi, Yukino Machida, Shinobu Kunugi, Yasuhiro Nishiyama, Shoji Matsune, Yasuyuki Negishi, Rimpei Morita

**Affiliations:** 1 Department of Microbiology and Immunology, Nippon Medical School, Tokyo, Japan; 2 Faculty of Applied Bioscience, Tokyo University of Agriculture, Tokyo, Japan; 3 Laboratory for Morphological and Biomolecular Imaging, Nippon Medical School, Tokyo, Japan; 4 Department of Analytic Human Pathology, Nippon Medical School, Tokyo, Japan; 5 The NODAI Genome Research Center, Tokyo University of Agriculture, Tokyo, Japan; 6 Department of Veterinary Pathology, Nippon Veterinary and Life Science University, Tokyo, Japan; 7 Department of Neurology, Faculty of Medical Sciences, University of Fukui, Fukui, Japan; 8 Department of Otolaryngology, Nippon Medical School Musashi Kosugi Hospital, Kanagawa, Japan; 9 Present Address: Tokyo University of Information Sciences, Chiba, Japan; 10 Present Address: Division of Cancer and Senescence Biology, Cancer Research Institute, Kanazawa University, Kanazawa, Japan; 11 Present Address: Division of Vaccine Immunology, International Institute for Zoonosis Control, Hokkaido University, Hokkaido, Japan; 12 Present Address: Laboratory of Veterinary Pathology, Tokyo University of Agriculture and Technology, Tokyo, Japan; 13 Present Address: Department of Otolaryngology, Japanese Red Cross Omori Hospital, Tokyo, Japan

**Keywords:** Cell death, Mucosal immunology, Cell death and immune response, Dysbiosis, Intestinal diseases

## Abstract

Aluminium ammonium sulphate (AAS) is a food additive used in some countries, but its effects on intestinal epithelial cells (IECs) remain poorly understood. We investigated whether AAS promotes IEC death and eosinophilic infiltration under dysbiotic conditions and whether heparin suppresses these effects. Mice pretreated with antibiotics or control water were orally administered AAS. IEC death was assessed by RNA sequencing, western blotting, and flow cytometry. Microbiota composition was analysed by 16S rRNA sequencing, and eosinophilic infiltration was evaluated by histology and flow cytometry. Immortalised murine IECs were stimulated with AAS in the presence or absence of bacterial components, and the effects of heparin were examined in vitro and in vivo. Antibiotic-induced dysbiosis altered IEC metabolic programmes and reduced polysaccharide-degrading bacteria. Under these conditions, AAS further reduced Actinobacteria, including *Bifidobacterium*, increased mitochondrial reactive oxygen species (mtROS), and promoted cleavage of caspase-1, caspase-6, caspase-8, caspase-11, interleukin-33, gasdermin D (GSDMD), and gasdermin E (GSDME) in IECs. These changes were associated with epithelial pyroptosis and eosinophilic infiltration in the small intestine. AAS induced similar cleavage events in IECs primed with lipopolysaccharide and lipoteichoic acid in vitro. Antioxidant studies showed that caspase-1/11-GSDMD activation was ROS-dependent, whereas caspase-6/8-GSDME activation involved ROS-independent mechanisms. Heparin suppressed these cleavage events, reduced IEC death both in vitro and in vivo, and attenuated eosinophilic inflammation. These findings identify a previously unrecognised pro-inflammatory effect of AAS in the dysbiotic gut and suggest low-dose heparin as a potential protective strategy for intestinal epithelial homoeostasis.

**Figure:**
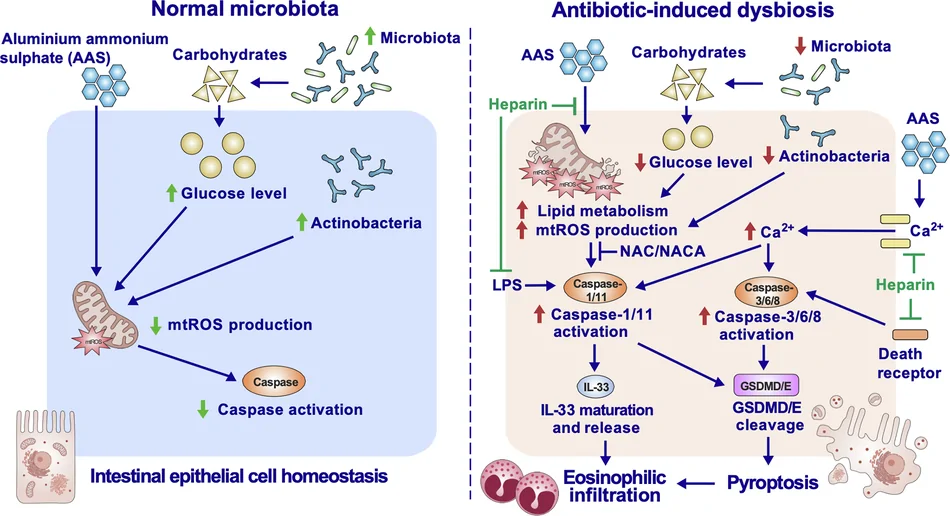

## Introduction

Eosinophilic gastrointestinal disorders (EGIDs) are characterised by eosinophilic infiltration of the gastrointestinal tract, which may involve the oesophagus, stomach, small intestine, or colon, leading to inflammation, tissue damage, and clinical symptoms [1]. The incidence and prevalence of EGIDs have been increasing in developed countries [2]. Food antigens, the gut microbiota, and antibiotic (ATB) exposure are implicated in their development [3], although the underlying mechanisms remain unclear. Epithelial barriers maintain tissue homoeostasis and protect against external factors such as pathogens and antigens [4]. Barrier defects are linked to allergic and inflammatory diseases, including asthma, atopic dermatitis, and eosinophilic oesophagitis [5]. Certain chemicals used in daily life can disrupt the epithelial barrier; for example, detergents cause barrier dysfunction and eosinophilic inflammation in the oesophagus [6] and increase interleukin (IL)-33 expression and eosinophilic inflammation in the airway [7].

Advances in food processing have led to the use of food additives. Potassium aluminium sulphate (PAS, E522) and ammonium aluminium sulphate (AAS, E523) are widely used in several countries, including the EU, Japan, and China, as firming agents, stabilisers, and colour fixatives [8]. In the United States, however, their use is limited to specific technical functions such as leavening and pH control under the GRAS (Generally Recognized as Safe) framework. Aluminium-containing compounds such as aluminium hydroxide and aluminium phosphate have long served as adjuvants in vaccines owing to their immune-enhancing properties [9, 10]. PAS has also shown immunostimulatory effects in animals and was once explored as a vaccine adjuvant; however, its use was not approved owing to reproducibility issues [9, 10]. Aluminium adjuvants induce Th2 responses, promote IgG1 and IgE production, and recruit eosinophils and monocytes [10]. Aluminium adjuvants are also known to promote NLRP3 inflammasome formation and caspase-1 activation in macrophages [11]. Activation of caspase-1/4/5/11 causes pyroptosis via gasdermin D (GSDMD) fragmentation and enhances IL-1β secretion [11]. Aluminium adjuvants also affect epithelial, endothelial, and stromal cells, promoting IL-33 cleavage under cellular stress [12], which is released through GSDMD pores [13] and activates ST2-expressing group 2 innate lymphoid cells, Th2 cells, and mast cells [12]. Based on these properties of aluminium adjuvants, we hypothesised that PAS and AAS may disrupt the epithelial barrier and promote type 2 inflammation in the gut.

Commensal bacteria and their metabolites, such as short-chain fatty acids, support epithelial barrier homoeostasis [14]. Dysbiosis is linked to barrier damage [5], and ATB treatment can induce intestinal dysfunction and NLRP3 activation [15]. Germ-free mice show increased numbers of intestinal and lung eosinophils compared with specific pathogen–free mice [16]. Therefore, we investigated whether ATB-induced dysbiosis exacerbates AAS-induced intestinal inflammation and epithelial injury.

We previously showed that oral cholera toxin adjuvant induces epithelial cell death, dendritic cell maturation, and cytotoxic T-cell responses in the intestine [17], suggesting that oral immunostimulants can trigger local inflammation. In this study, we examined whether oral AAS induces epithelial cell death and eosinophilic infiltration in mice with ATB-induced dysbiosis, using an ampicillin, vancomycin, neomycin, and metronidazole (AVNM) cocktail [18]. We found that AAS reduced the abundance of Actinobacteria and promoted cleavage of IL-33, caspases—including caspase-11—and pyroptosis executioner proteins in intestinal epithelial cells (IECs), which was associated with epithelial death and eosinophilic inflammation. Importantly, heparin, which has been reported to inhibit caspase-11 independently of its anticoagulant activity [19], suppressed multiple caspases involved in pyroptosis and alleviated AAS-induced epithelial damage and eosinophilic infiltration in this context.

## Results

### AAS promotes eosinophilic infiltration in the small intestine, with enhanced effects under ATB-treated conditions

To investigate the pro-inflammatory potential of aluminium-containing compounds, we first assessed whether oral administration of aluminium salts, PAS or AAS, promoted intestinal eosinophilic infiltration in mice. C57BL/6 J mice received 10⁻⁵ mol AAS or vehicle (PBS) via oral gavage once weekly for 3 weeks. AAS administration increased the total number of lamina propria (LP) cells and the proportion of Siglec-F⁺ CCR3⁺ MHC class II^-^ eosinophils within the LP fraction of the small intestine compared to the PBS group (Supplementary Fig. 1A–D). This effect was more pronounced with AAS than PAS. In contrast, neither compound significantly altered eosinophil counts in peripheral blood (Supplementary Fig. 1A–D).

**Fig. 1:**
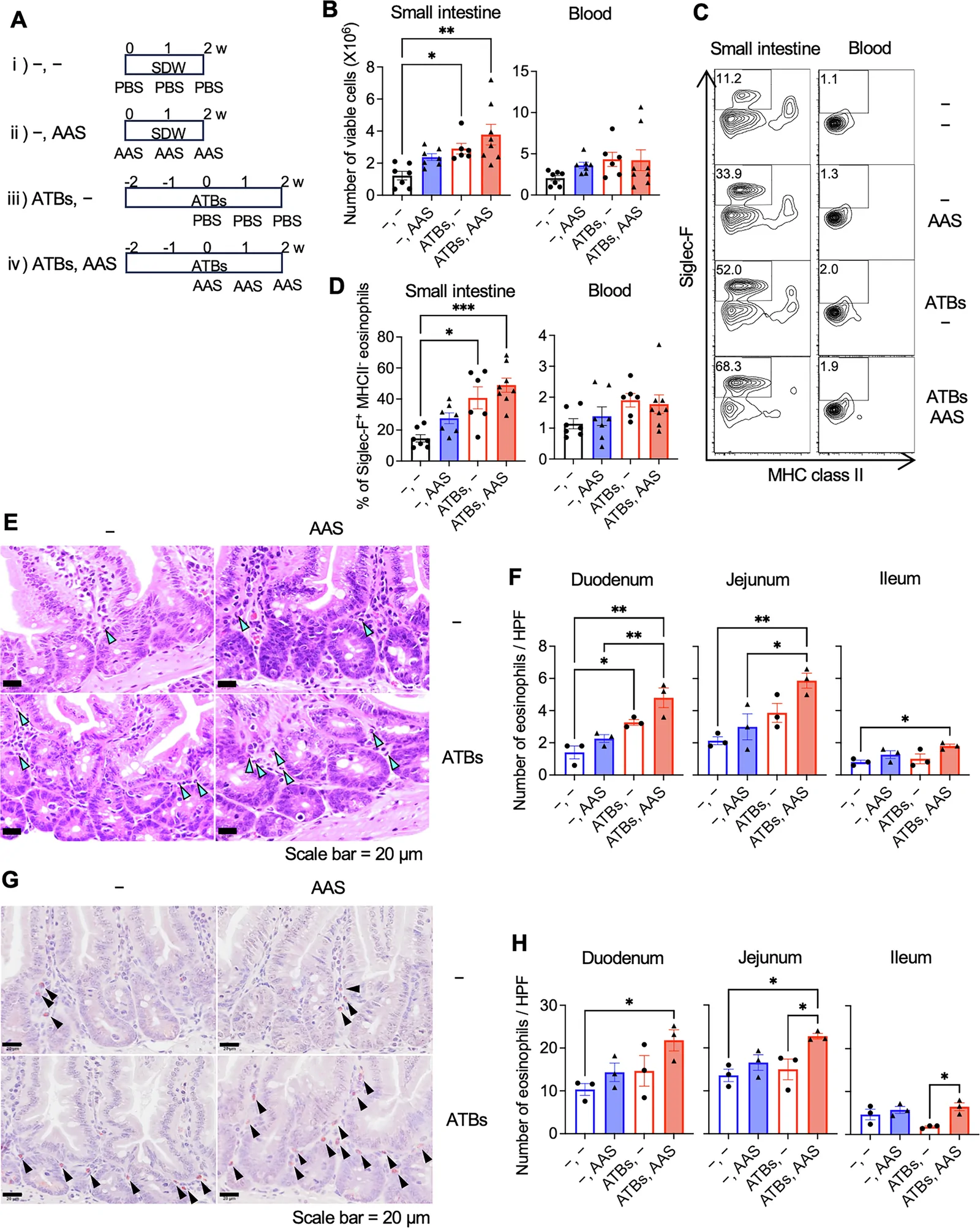
Ammonium aluminium sulphate (AAS) promotes eosinophilic infiltration in the small intestine, with enhanced effects under antibiotic (ATB)-treated conditions. **A** Experimental design: C57BL/6 J mice were treated with either sterile distilled water (SDW) or ATBs, followed by oral administration of AAS (10⁻⁵ mol) or PBS once weekly for 3 weeks. Small intestinal tissues and peripheral blood were harvested 16 h after the final dose. **B** Viable eosinophil counts in the small intestinal lamina propria (LP) and peripheral blood, assessed by trypan blue exclusion. Representative flow cytometry plots (**C**) and quantification (**D**) of Siglec-F⁺ MHC class II⁻ eosinophils in LP and blood. Data are presented as mean ± standard error of the mean (SEM) (*n* = 6–7 mice/group). *P < 0.05, **P < 0.01, ***P < 0.005 by Kruskal–Wallis test. Haematoxylin and eosin (HE) staining (**E**) and quantification of eosinophils per high-power field (HPF) in jejunal sections (**F**). Direct fast scarlet (DFS) staining (**G**) and quantification of eosinophils per HPF (**H**). Arrows indicate eosinophils. Data are presented as mean ± SEM (*n* = 3 mice/group). *P < 0.05, **P < 0.01 by one-way analysis of variance (ANOVA). Statistical comparisons between indicated groups are shown in the figure.

Next, we examined the effect of AAS under conditions of ATB-induced dysbiosis. Mice were treated with or without a cocktail of four ATBs (ampicillin, vancomycin, neomycin, and metronidazole; AVNM), and orally administered 1 × 10⁻⁵ mol AAS or PBS weekly for 3 weeks. Small intestinal tissues and blood were collected 16 hours after the final dose (Fig. 1A). AVNM treatment alone increased intestinal LP cell numbers and eosinophil proportions, and AAS administration was associated with a further increase under dysbiotic conditions (Fig. 1B–D). Histological analysis with haematoxylin and eosin (HE) (Fig. 1E, F) and Direct Fast Scarlet (DFS) staining (Fig. 1G, H) revealed prominent eosinophilic infiltration in the duodenum and jejunum of AVNM + AAS-treated mice. No significant changes were observed in blood eosinophils or intestinal neutrophils (Fig. 1C, D; Supplementary Fig. 1E).

Collectively, these findings suggest that AAS contributes to eosinophilic infiltration in the murine small intestine, with effects that are enhanced under ATB-induced dysbiosis.

### AAS promotes epithelial cell death in the intestine, with enhanced effects under ATB-treated conditions

We next examined whether epithelial cell death precedes eosinophilic infiltration in the murine intestine. Oral administration of 10⁻⁵ mol AAS increased the percentage of dying/dead (Zombie⁺ Annexin V⁺) EpCAM⁺ CD45⁻ intestinal epithelial cells compared with PBS treatment (Supplementary Fig. 2A–D). In contrast, neither AAS nor PAS affected cell death in CD45⁺ leucocytes (Supplementary Fig. 2B–D).

**Fig. 2:**
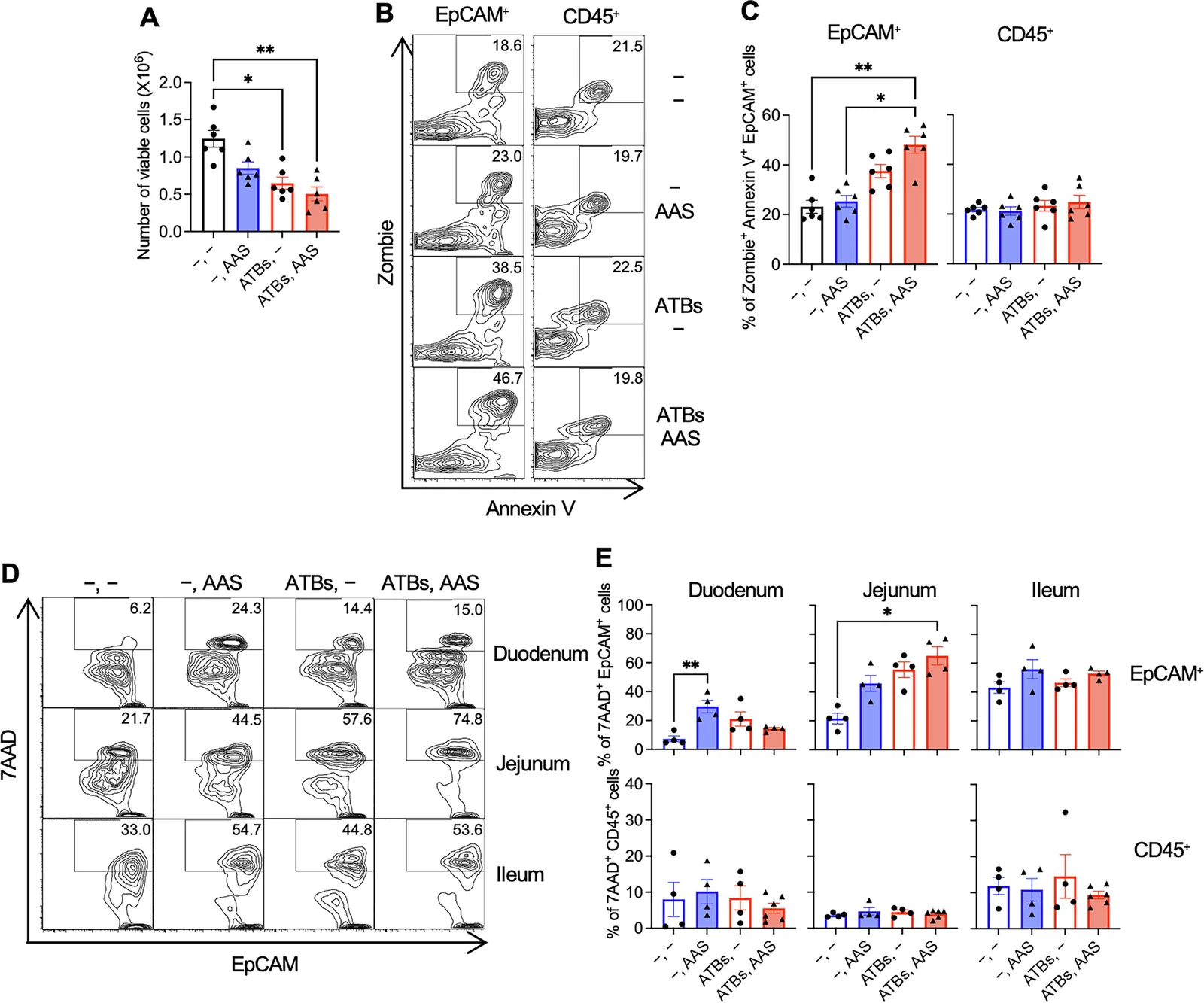
AAS promotes epithelial cell death in the intestine, with enhanced effects under ATB-treated conditions. **A** Number of viable intestinal epithelial cells (IECs) isolated from mice 16 h after oral administration of AAS (10⁻⁵ mol) or PBS, following 2 weeks of treatment with or without ATBs. Representative flow cytometry plots (**B**) and quantification (**C**) of dying/dead (Zombie⁺ Annexin V⁺) EpCAM⁺ IECs and CD45⁺ leucocytes from the small intestine. Data are presented as mean ± SEM (*n* = 6 mice/group). *P < 0.05, **P < 0.01 by Kruskal–Wallis test. Regional analysis of cell death in the duodenum, jejunum, and ileum. Representative plots of 7-AAD⁺ EpCAM⁺ IECs (**D**) and quantification of dying/dead EpCAM⁺ and CD45⁺ cells (**E**) in each intestinal segment. Data are presented as mean ± SEM (*n* = 4 mice/group). *P < 0.05, **P < 0.01 by Kruskal–Wallis test.

To assess the impact of ATB exposure on AAS-induced epithelial injury, mice were pretreated with AVNM starting 2 weeks before the first AAS administration, and ATBs were continued throughout the AAS treatment period until sacrifice. Mice then received 10⁻⁵ mol AAS or PBS, and IECs were isolated 1 or 16 h post-administration. ATB treatment alone led to a reduction in viable IECs and a modest increase in epithelial cell death. AAS administration showed a trend toward a further increase, although this did not reach statistical significance (Supplementary Fig. 2E; Fig. 2A–C). CD45⁺ leucocyte death remained unchanged (Fig. 2B, C).

Regional analysis revealed a significant increase in dying/dead IECs in the jejunum of ATB-pretreated mice following AAS administration (Fig. 2D, E). In contrast, duodenal epithelial cell death was observed even in the absence of ATB pretreatment (Fig. 2D, E). CD45⁺ cell death was not increased in any intestinal segment under any condition (Fig. 2E; Supplementary Fig. 2F).

These findings suggest that AAS promotes epithelial cell death in the murine small intestine, with effects that are enhanced under ATB-induced dysbiotic conditions.

### AAS induces cleavage of caspases, gasdermins, and IL-1 family cytokines in IECs, with enhanced responses under ATB-treated conditions

**Fig. 3:**
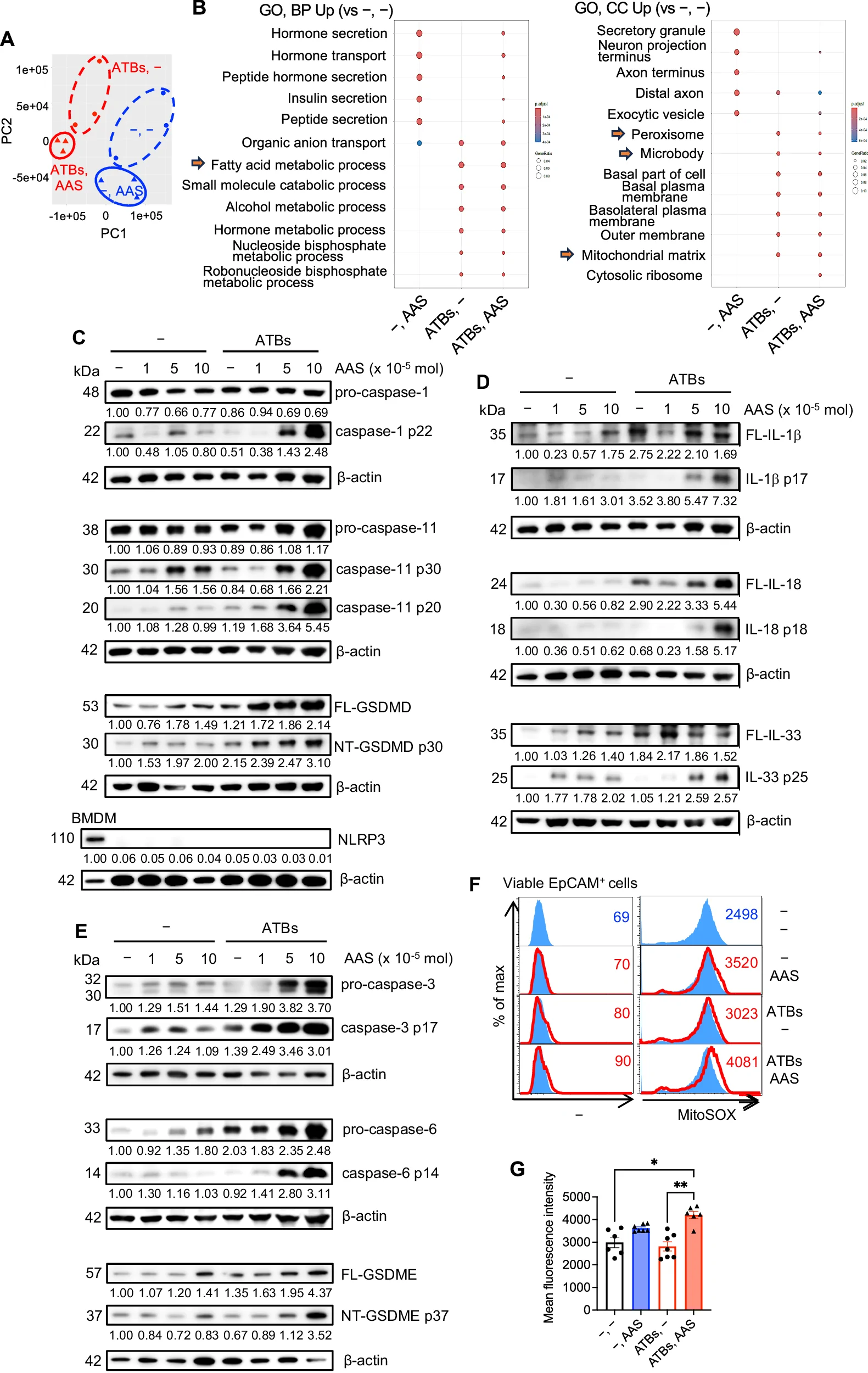
AAS induces cleavage of caspases, gasdermins, and IL-1 family cytokines in IECs, with enhanced responses under ATB-treated conditions. **A** Principal component analysis (PCA) of RNA-seq data from murine IECs isolated 1 h after AAS administration (10⁻⁵ mol), following 2 weeks of treatment with or without ATBs. **B** Gene Ontology enrichment analysis of upregulated differentially expressed genes (DEGs) compared with untreated controls, in biological process (BP) and cellular component (CC) categories. *n* = 3 mice/group. **C**–**E** Immunoblot analysis of pooled IEC lysates from 5 mice per group, collected 4 h after AAS administration (1–10 × 10⁻⁵ mol) following ATB or no treatment. **C** Cleavage of caspase-1, caspase-11, and gasdermin D (GSDMD), with no detectable expression of NLRP3. BMDMs were used as a positive control for NLRP3 expression. **D** Cleavage of IL-1β, IL-18, and IL-33. **E** Cleavage of caspase-3, caspase-6, and gasdermin E (GSDME). Most target proteins were analysed on separate gels/membranes, and the corresponding β-actin loading control is shown for each blot. IL-1β (**D**) was analysed on the same gel as caspase-1 (**C**). Numbers below the bands indicate densitometric values normalised to the corresponding β-actin signal, with the control set as 1. **F**, **G** Mitochondrial reactive oxygen species (mtROS) levels in viable EpCAM⁺ IECs assessed using MitoSOX Red. **F** Representative histograms. **G** Mean fluorescence intensity quantification. Data are mean ± SEM (*n* = 6–7 mice/group). *P < 0.05, **P < 0.01 by Kruskal–Wallis test.

To explore the molecular basis of epithelial cell death, we performed RNA sequencing of sorted EpCAM⁺ CD45⁻ IECs from mice treated with AAS, with or without prior ATB exposure (Supplementary Fig. 3A). Principal component analysis revealed distinct transcriptional profiles among the untreated (−/−), AAS-only (−/AAS), ATBs-only (ATBs/−), and combined ATBs + AAS (ATBs/AAS) groups (Fig. 3A). AAS treatment alone upregulated inflammatory genes including *Il33*, *Il17c*, and *Nfkb2*, while ATB treatment induced gene expression associated with fatty acid metabolism (*Ppara*, *Pdk4*, *Cd36*) and cell death (*Casp6*) (Supplementary Fig. 3B, C). Notably, *Casp6* expression was further enhanced in the ATBs/AAS group. Gene ontology enrichment analysis showed that AAS-affected genes were primarily involved in hormone and peptide secretion. In contrast, ATB exposure upregulated genes associated with fatty acid metabolism, peroxisome, microbody, and mitochondrial matrix, and downregulated those related to membrane rafts and apical plasma membranes (Fig. 3B; Supplementary Fig. 3D), suggesting altered metabolic function and membrane vulnerability in IECs under dysbiosis.

Western blot analysis of IECs revealed that cleaved caspase-6 (p14) and caspase-11 (p20) were detectable 4 h after low-dose AAS (1 × 10⁻⁵ mol) administration, with more prominent cleavage observed in the ATB-treated group (Supplementary Fig. 4A). Higher doses (5–10 × 10⁻⁵ mol) of AAS induced cleavage of caspase-1, caspase-11, GSDMD, IL-1β, IL-18, and IL-33, with markedly enhanced cleavage in ATB-treated mice (Fig. 3C, D). Cleaved caspase-1, caspase-11, and GSDMD, key mediators of pyroptosis, were particularly elevated in the ATBs/AAS group. NLRP3 expression was not detected in IECs, despite clear detection in bone marrow-derived macrophages (BMDMs) used as a positive control (Fig. 3C). In addition, AAS promoted cleavage of caspase-3, caspase-6, and GSDME in IECs under ATB-treated conditions (Fig. 3E). Gasdermin E (GSDME), a known pyroptotic effector downstream of caspase-3/6/7/8 [20], showed greater cleavage in the ATBs/AAS group (Fig. 3E). Caspase-9 activation, however, was not observed (Supplementary Fig. 4B).

MitoSOX analysis further demonstrated that AAS markedly increased mitochondrial reactive oxygen species (mtROS) levels in both EpCAM⁺ IECs and CD45⁺ leucocytes, with higher levels observed following ATB treatment (Fig. 3F, G; Supplementary Fig. 4C–E).

Collectively, these findings indicate that AAS promotes mtROS production and cleavage of caspase-1, caspase-3, caspase-6, caspase-11, GSDMD, GSDME, IL-1β, IL-18, and IL-33 in IECs, with these responses being enhanced under dysbiotic conditions. Although NLRP3 was not detected in this experimental system, these cleavage patterns support activation of pyroptosis-related pathways, while the possible involvement of other inflammasome sensors remains to be determined.

### AAS is associated with reduced Actinobacteria, including *Bifidobacterium*, in IECs under ATB-induced dysbiosis

**Fig. 4:**
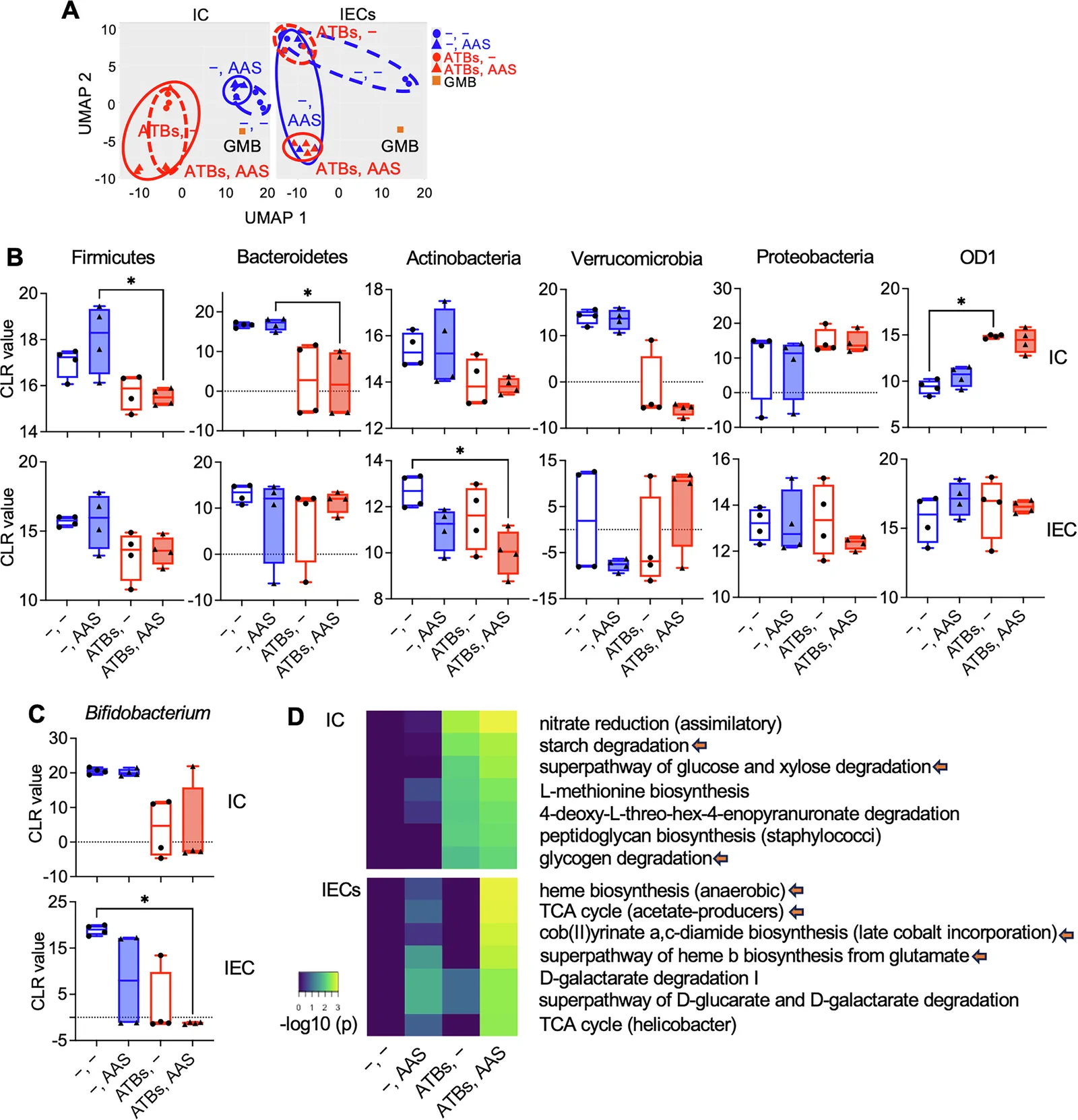
AAS is associated with reduced Actinobacteria, including *Bifidobacterium*, in IECs under ATB-induced dysbiosis. Intestinal contents (IC) and IECs were isolated from mice 1 h after AAS administration (10⁻⁵ mol), following 2 weeks of treatment with or without ATBs, and subjected to 16S rRNA amplicon sequencing. **A** Uniform Manifold Approximation and Projection (UMAP) analysis showing microbial composition in IC and IECs from untreated (blue circle, dotted line), AAS-treated (blue triangle, solid line), ATB-treated (red circle, dotted line), AAS + ATB-treated mice (red triangle, solid line), and gut microbiome standard (GMB). **B** Comparison of centred log-ratio (CLR)-transformed phylum-level bacterial abundance in IC and IECs. **C** CLR-transformed abundance of *Bifidobacterium*. **D** Heatmap of predicted KEGG pathways downregulated by AAS and/or ATB treatment in IC and IECs (PICRUSt2 analysis). Data are shown as box-and-whisker plots (*n* = 4 mice/group). *P < 0.05 by Kruskal–Wallis test.

Mitochondrial ROS production and caspase-11 activation are associated with bacterial invasion of the cytoplasm [21]. To investigate the effects of AAS and ATBs on the gut microbiota, we performed 16S rRNA sequencing of intestinal contents and IECs from mice treated with AAS with or without prior ATB treatment. Bacterial genes detected in IECs were more diverse and distinct from those in intestinal contents (Supplementary Fig. 5A, B). Uniform manifold approximation and projection (UMAP) analysis showed differences in the microbial composition between the intestinal contents of ATB-treated and -untreated mice (Fig. 4A). Among ATB-treated mice, bacterial genes detected in IECs were different between AAS-treated and non-AAS-treated mice (Fig. 4A). ATB treatment was associated with reduced abundance of Firmicutes and Bacteroidetes in the intestinal contents, with lower levels observed in the ATBs/AAS group (Fig. 4B). Notably, AAS administration under ATB treatment was associated with a reduction of Actinobacteria, including *Bifidobacterium* in IECs (Fig. 4B, C). Segmented filamentous bacteria (*Candidatus* Arthromitus), known to promote Th17 differentiation and neutrophil recruitment [22], were reduced in IECs of ATB-treated mice (Supplementary Fig. 5C). This was consistent with reduced *Il17c* expression in IECs (Supplementary Fig. 3B, C) and the absence of intestinal neutrophil infiltration following ATB treatment (Supplementary Fig. 1E). Bacterial pathway analysis revealed that ATB treatment reduced microbial pathways related to degradation of polysaccharides, such as starch and glycogen, glucose, and xylose, in the intestinal contents (Fig. 4D). Interestingly, AAS administration after ATB treatment suppressed bacterial haeme biosynthesis, the TCA cycle, and cobalt incorporation in IECs (Fig. 4D). Actinobacteria produce haeme and cobalamin, which are essential for host antioxidant defences [23]. Reduced levels of haeme and cobalamin may impair mitochondrial function and promote oxidative stress [24]. *Bifidobacterium* also contributes to cobalamin production and possesses antioxidant properties [25]. Therefore, the AAS-associated reduction of Actinobacteria, including *Bifidobacterium*, may contribute to increased oxidative stress, consistent with increased mtROS levels observed in IECs (Fig. 3F, G).

### AAS enhances pyroptotic cell death in IECs under priming conditions with lipopolysaccharide (LPS) and lipoteichoic acid (LTA)

**Fig. 5:**
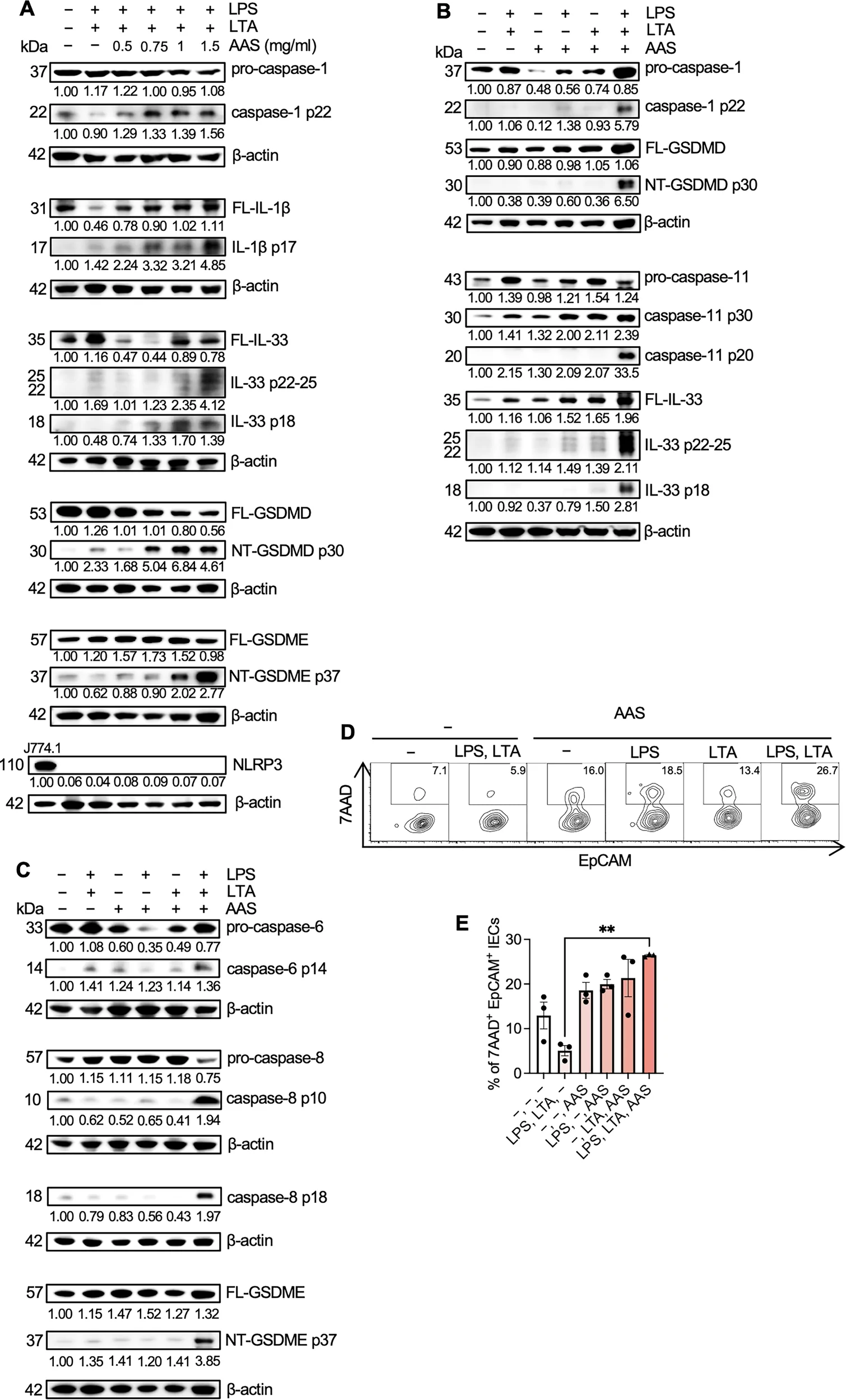
AAS enhances pyroptotic cell death in IECs under LPS/LTA priming conditions. **A** Cleavage of caspase-1, IL-1β, IL-33, GSDMD, and GSDME, with no detectable expression of NLRP3 in CI-IEC lysates primed with LPS and LTA (10 µg/mL each for 4 h) and stimulated with AAS (0.5–1.5 mg/mL for 4 h). J774.1 cells were used as a positive control for NLRP3 expression. **B**, **C** Immunoblot analysis of CI-IEC lysates primed with or without LPS and/or LTA and stimulated with AAS (1 mg/mL). **B** Cleavage of caspase-1, caspase-11, IL-33, and GSDMD. **C** Cleavage of caspase-6, caspase-8, and GSDME. Most target proteins were analysed on separate gels/membranes, and the corresponding β-actin loading control is shown for each blot. GSDMD was analysed on the same gel as caspase-1, and IL-33 was analysed on the same gel as caspase-11 (**B**). Numbers below the bands indicate densitometric values normalised to the corresponding β-actin signal, with the control set as 1. Flow cytometry analysis of dying/dead 7-AAD⁺ CI-IECs following AAS stimulation under LPS/LTA priming. Representative plots (**D**) and quantification (**E**). Data are presented as mean ± SEM from three wells/group. **P < 0.01 by Kruskal–Wallis test. Data are representative of ≥2 independent experiments. LPS lipopolysaccharide, LTA lipoteichoic acid, CI-IEC CI-muADINTESTI.

Alum has been shown to activate caspase-1 and induce pyroptosis through the NLRP3 inflammasome and GSDMD in macrophages and monocytes following LPS priming [11, 26]. To assess whether a similar mechanism operates in IECs, we utilised immortalised murine IECs, CI-muADINTESTI (CI-IECs). These cells were EpCAM⁺ CD45⁻ and expressed pro-caspase-1, caspase-6, caspase-8, caspase-11, full-length (FL) IL-33, FL-GSDMD, FL-GSDME, Toll-like receptor (TLR)2, and TLR4, but lacked detectable NLRP3 expression, consistent with the molecular profile of primary IECs (Supplementary Fig. 6A, B). AAS enhanced cleavage of caspase-1, caspase-6, caspase-8, caspase-11, IL-1β, IL-33, GSDMD, and GSDME under LPS/LTA priming conditions, without detectable NLRP3 expression, accompanied by increased 7-AAD^+^ cell death in CI-IECs (Fig. 5A–E). Reduced cleavage and cell death were observed in the absence of AAS, LPS, or LTA, and the cleavage patterns were not affected by ATB pretreatment (Fig. 5B–E, Supplementary Fig. 6C). Together, these results demonstrate that AAS promotes pyroptotic signalling in IECs under LPS/LTA priming conditions in the absence of detectable NLRP3 expression.

### ROS-dependent and -independent pathways contribute to AAS-induced IEC death

**Fig. 6:**
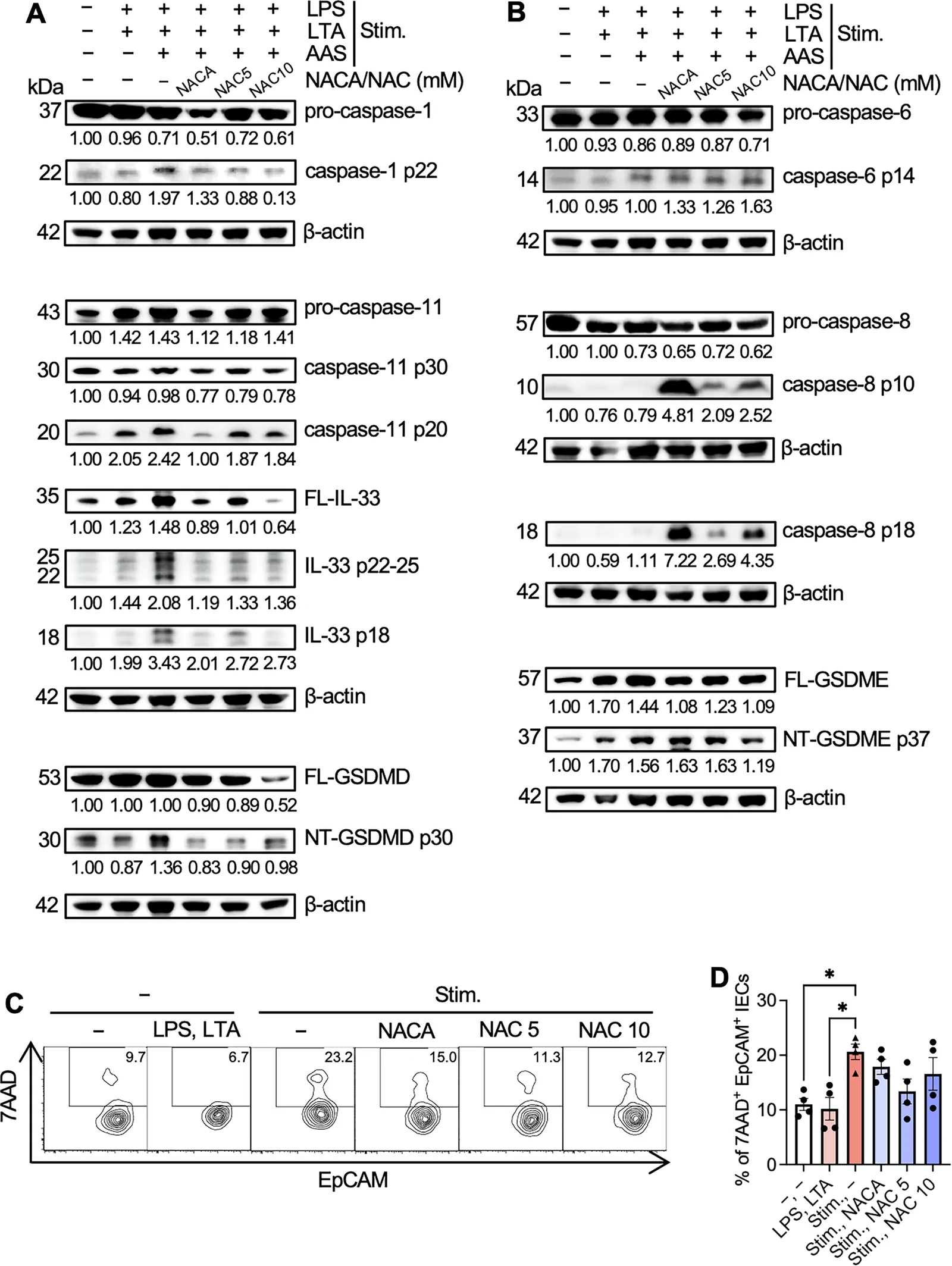
ROS-dependent and -independent pathways contribute to AAS-induced IEC death. **A**, **B** Immunoblot of CI-IEC lysates pretreated with NACA (4 mM) or NAC (5 or 10 mM) prior to AAS stimulation (1 mg/mL for 4 h) under LPS/LTA priming. **A** Cleavage of caspase-1, caspase-11, IL-33, and GSDMD. **B** Cleavage of caspase-6, caspase-8, and GSDME. Most target proteins were analysed on separate gels/membranes, and the corresponding β-actin loading control is shown for each blot. IL-33 was analysed on the same gel as caspase-11 (**A**). Caspase-6 (**B**) was analysed on the same gel as caspase-1 (**A**). Numbers below the bands indicate densitometric values normalised to the corresponding β-actin signal, with the control set as 1. **C, D** Flow cytometry analysis of dying/dead 7-AAD⁺ CI-IECs pretreated with NACA or NAC before AAS stimulation under LPS/LTA priming. Representative plots (**C**) and quantification (**D**). Data are presented as mean ± SEM from four wells/group. *P < 0.05 by one-way ANOVA. Data are representative of ≥2 independent experiments. NACA N-acetylcysteine amide, NAC N-acetylcysteine.

To examine the contribution of oxidative stress, we tested the antioxidants N-acetylcysteine amide (NACA) and N-acetylcysteine (NAC) [27]. Both NACA and NAC suppressed AAS-induced cleavage of caspase-1 and caspase-11, IL-33, and GSDMD, whereas they did not inhibit cleavage of caspase-6, caspase-8, or GSDME (Fig. 6A, B). Notably, NACA enhanced caspase-8 cleavage, suggesting a potential pro-apoptotic shift under these conditions. Consistent with these biochemical findings, NACA and NAC only partially reduced AAS-induced cell death and did not fully prevent it (Fig. 6C, D). These results suggest that ROS contributes to AAS-induced epithelial injury but is not sufficient to account for the full spectrum of caspase and gasdermin activation, indicating the involvement of both ROS-dependent and ROS-independent pathways in IEC pyroptosis.

### Heparin suppresses AAS-induced pyroptotic signalling and IL-33 cleavage in IECs in vitro

**Fig. 7:**
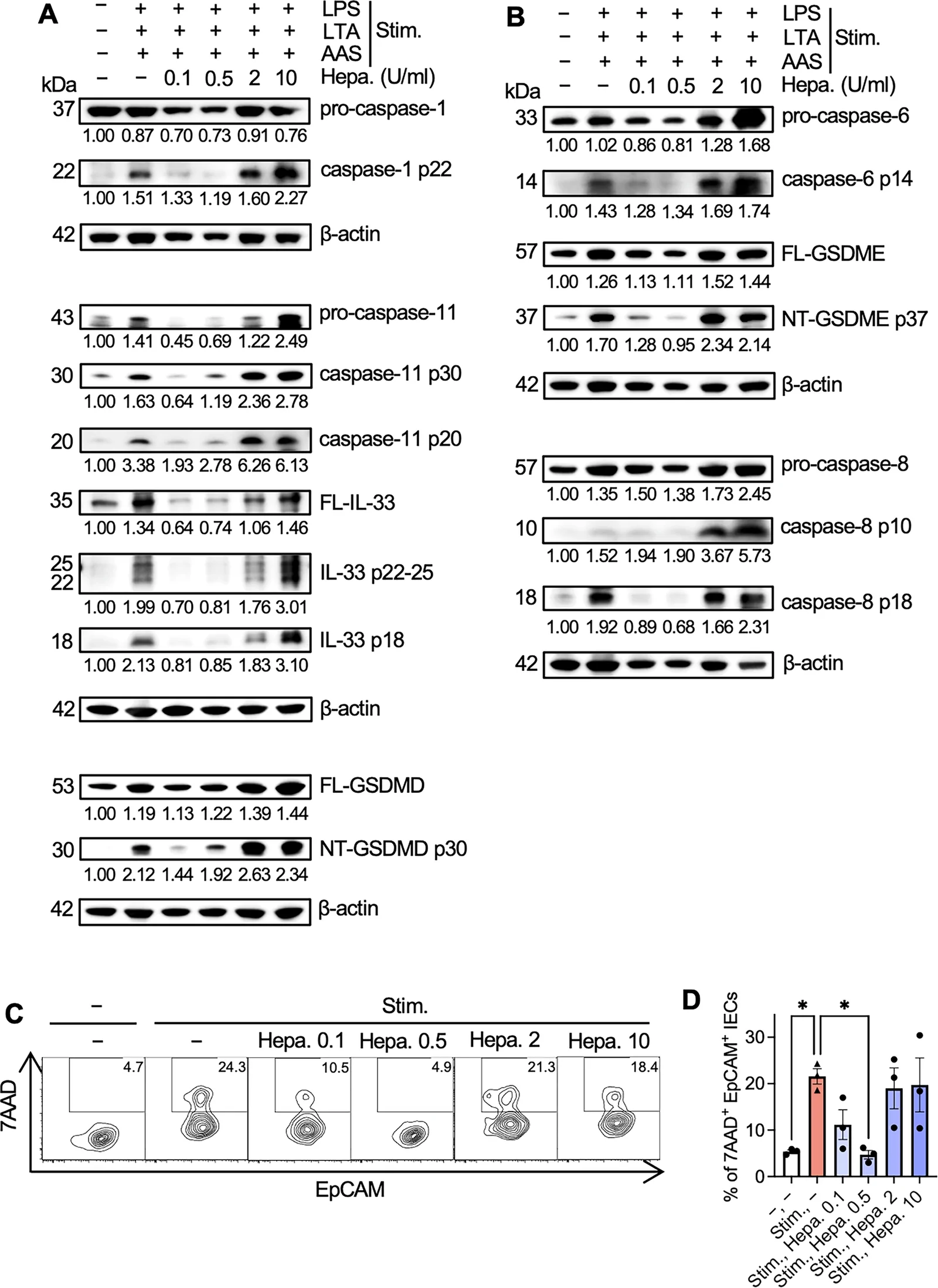
Heparin suppresses AAS-induced pyroptotic signalling and IL-33 cleavage in IECs in vitro. **A**, **B** Immunoblot of CI-IEC lysates pretreated with heparin (0.1–10 U/mL) prior to AAS stimulation (1 mg/mL for 4 h) under LPS/LTA priming. **A** Cleavage of caspase-1, caspase-11, IL-33, and GSDMD. **B** Cleavage of caspase-6, caspase-8, and GSDME. Most target proteins were analysed on separate gels/membranes, and the corresponding β-actin loading control is shown for each blot. IL-33 was analysed on the same gel as caspase-11 (**A**). GSDME was analysed on the same gel as caspase-6, and caspase-8 cleavage products (p10 and p18) were analysed on the same gel (**B**). Numbers below the bands indicate densitometric values normalised to the corresponding β-actin signal, with the control set as 1. Flow cytometry analysis of dying/dead 7-AAD⁺ CI-IECs pretreated with heparin before AAS stimulation under LPS/LTA priming. Representative plots (**C**) and quantification (**D**). Data are presented as mean ± SEM from three wells/group. *P < 0.05 by one-way ANOVA. Data are representative of ≥2 independent experiments.

Heparin, which inhibits caspase-11 independently of its anticoagulant properties [19], was tested for its protective effect against AAS-induced pyroptosis. Treatment with 0.5 U/mL heparin suppressed the cleavage of caspase-1, caspase-6, caspase-8, caspase-11, IL-33, GSDMD, and GSDME (Fig. 7A, B), and significantly reduced CI-IEC cell death (Fig. 7C, D). In contrast, higher concentrations of heparin (2–10 U/mL) failed to suppress AAS-induced signalling or cell death (Fig. 7A–D). Heparin itself did not affect cell viability, as treatment with various concentrations of heparin (0.5–200 U/mL) did not alter the percentage of 7-AAD⁺ cells in CI-IECs (Supplementary Fig. 6D, E). In contrast, the caspase-1 inhibitor belnacasan (VX-765) [28] failed to block GSDMD cleavage in AAS-treated CI-IECs (Supplementary Fig. 6F). These results indicate that AAS promotes pyroptotic cell death in IECs through multiple caspase pathways under LPS and LTA priming conditions. Together, these findings indicate that low-dose heparin suppresses AAS-induced pyroptotic signalling in IECs by inhibiting both ROS-dependent (caspase-1/11–GSDMD) and ROS-independent (caspase-6/8–GSDME) pathways.

### Heparin suppresses AAS-induced pyroptotic signalling in IECs and epithelial injury in vivo

**Fig. 8:**
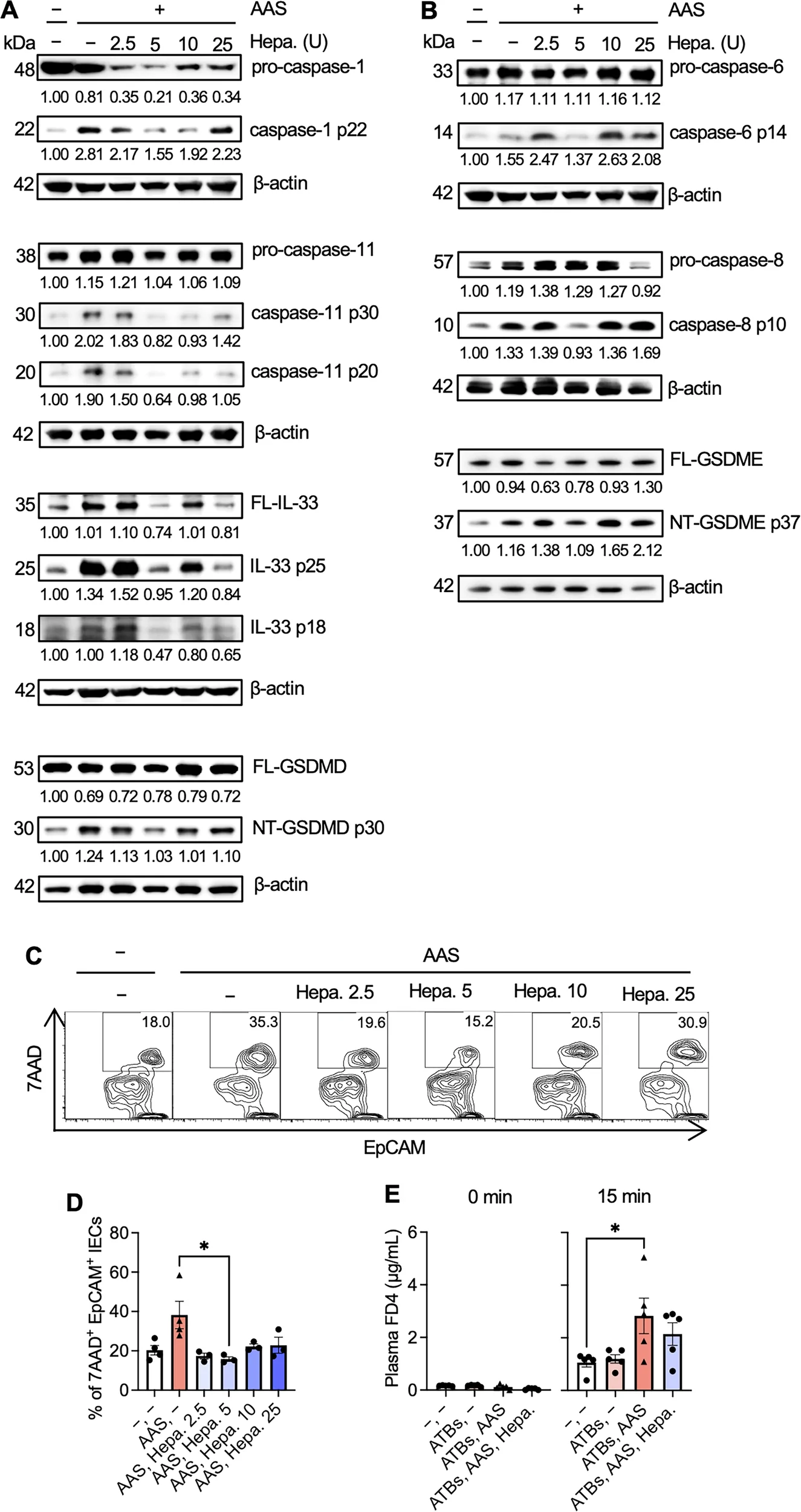
Heparin suppresses AAS-induced pyroptotic signalling in IECs and epithelial injury in vivo. **A**, **B** Immunoblot analysis of IEC lysates from ATB-treated mice injected with heparin (2.5–25 U) 30 min before oral administration of AAS (5 × 10⁻⁵ mol). IECs were isolated 4 h after AAS administration. **A** Cleavage of caspase-1, caspase-11, IL-33, and GSDMD. **B** Cleavage of caspase-6, caspase-8, and GSDME. Data represent pooled IEC lysates from 5 mice per group. Most target proteins were analysed on separate gels/membranes, and the corresponding β-actin loading control is shown for each blot. Caspase-6 (**B**) was analysed on the same gel as caspase-1 (**A**). Numbers below the bands indicate densitometric values normalised to the corresponding β-actin signal, with the control set as 1. Flow cytometry analysis of dying/dead 7-AAD⁺ CD45⁻ EpCAM⁺ IECs. Representative plots (**C**) and quantification of dying/dead IECs (**D**). Data are presented as mean ± SEM (*n* = 3–4 mice/group). *P < 0.05 by Kruskal–Wallis test. **E** Plasma FITC–dextran (FD4) leakage assay. Intestinal permeability was evaluated 15 min after oral gavage of FD4 in ATB-treated mice administered AAS with or without injection of 5 U heparin. Data are presented as mean ± SEM (*n* = 5 mice/group). *P < 0.05 by one-way ANOVA.

Furthermore, we assessed whether heparin administration could suppress AAS-induced epithelial injury and eosinophilic inflammation in vivo. Mice received subcutaneous injections of heparin (2.5–25 U) prior to AAS administration during ATB treatment. Among the tested doses, 5 U heparin showed the most pronounced inhibitory effects on the cleavage of caspase-1, caspase-6, caspase-8, caspase-11, IL-33, GSDMD, and GSDME in IECs (Fig. 8A, B), and markedly reduced epithelial cell death (Fig. 8C, D). Heparin at 5 U also tended to reduce plasma FITC–dextran (FD4) levels following oral FD4 administration, suggesting partial preservation of barrier integrity (Fig. 8E). In contrast, a higher dose of 25 U heparin failed to fully block cleavage of caspase-1, caspase-6, caspase-8, and GSDME or epithelial cell death (Fig. 8A–D). Treatment with the caspase-1 inhibitor belnacasan, administered either orally or intraperitoneally, did not prevent pyroptosis-related protein cleavage and, in some cases, enhanced cleavage; it also failed to suppress AAS-induced epithelial cell death (Supplementary Fig. 7A–D). Collectively, these findings indicate that low-dose heparin, but not high-dose heparin or belnacasan, effectively suppresses pyroptotic epithelial cell death in the dysbiotic intestine exposed to AAS.

### Heparin ameliorates AAS-induced eosinophilic infiltration in the murine small intestine

**Fig. 9:**
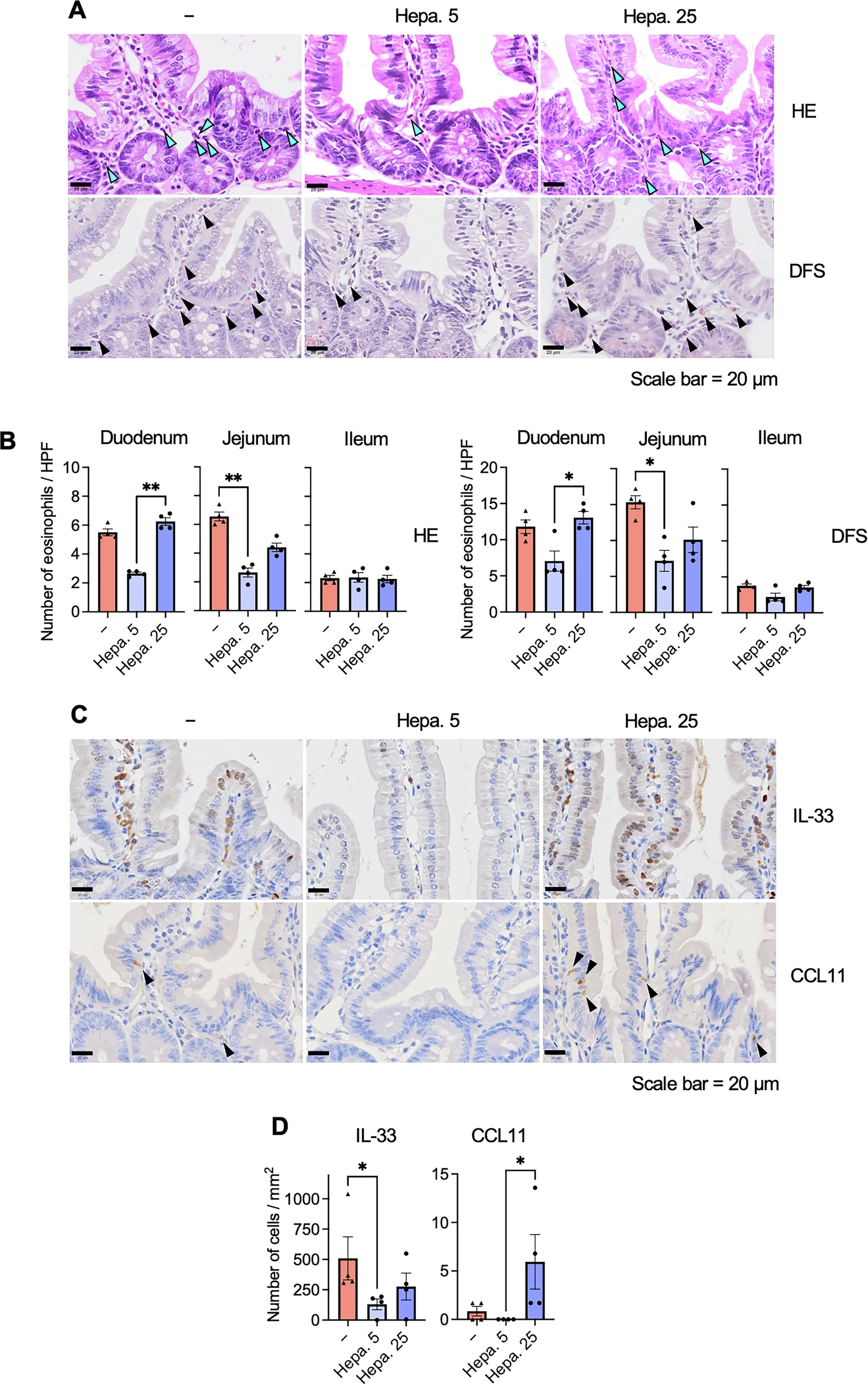
Heparin ameliorates AAS-induced eosinophilic infiltration in the murine small intestine. **A**–**D** Histological and immunohistochemical analysis of eosinophilic inflammation in the small intestine after 3 weekly doses of AAS (5 × 10⁻⁵ mol) with or without injection of heparin (5 or 25 U). The small intestine was collected 16 h after the final AAS administration and stained with HE or DFS (**A**, **B**) and immunostained for IL-33 and CCL11 (**C**, **D**). **A** Representative HE and DFS staining in the jejunum. Arrows indicate eosinophils. **B** Quantification of eosinophils per HPF. Representative IL-33 and CCL11 immunostaining (**C**) and their quantification (**D**) in the jejunum. Data are presented as mean ± SEM (*n* = 4 mice/group). *P < 0.05, **P < 0.01 by Kruskal–Wallis test.

Finally, we assessed whether heparin attenuated AAS-induced eosinophilic inflammation in vivo. Injection of 5 U heparin ameliorated eosinophilic infiltration in the small intestine (Fig. 9A, B) and suppressed the intestinal expression of IL-33 and CCL11 (Fig. 9C, D). In contrast, 25 U heparin failed to suppress eosinophilic infiltration or intestinal IL-33 expression and instead increased epithelial CCL11 expression (Fig. 9C, D). In jejunal and colonic tissue homogenates, neither the AAS-induced elevation of CCL11 and IL-5 nor any suppressive effect of heparin could be detected (Supplementary Fig. 7E). Coagulation status remained unaffected at doses up to 10 U, whereas 25 U heparin completely inhibited blood clotting in two out of three mice. Treatment with belnacasan, administered intraperitoneally, failed to suppress eosinophilic infiltration (Supplementary Fig. 7F, G). Taken together, these findings indicate that low-dose heparin, but not high-dose heparin or belnacasan, effectively suppresses eosinophilic inflammation in the dysbiotic intestine exposed to AAS.

## Discussion

Our study demonstrates that AAS, an aluminium-containing food additive, exacerbates pyroptotic epithelial cell death and promotes IL-33 maturation in IECs, ultimately leading to eosinophilic infiltration under dysbiotic conditions. Eosinophils release cytotoxic proteins, such as major basic protein, and various inflammatory mediators, leading to mucosal inflammation and epithelial tissue damage [29]. Similar to aluminium adjuvants, AAS induces inflammatory cell death, thereby disrupting the mucosal barrier and initiating intestinal immune activation. Mature IL-33, released through GSDMD pores, promotes type 2 inflammation and allergic responses [30]. Consistent with the marked eosinophilic infiltration observed in AAS-treated dysbiotic mice, immunohistochemistry showed epithelial expression of IL-33 and CCL11 (eotaxin) in the small intestinal mucosa. These findings support the involvement of IL-33–CCL11-associated pathways in eosinophil recruitment in this model. Importantly, this sequence of events aligns with previous evidence showing that epithelial injury precedes eosinophilic infiltration: epithelial-derived IL-33 is rapidly released following epithelial damage and subsequently drives type-2 immune activation and eosinophil accumulation [31, 32]. Thus, the early occurrence of IEC pyroptosis and IL-33 cleavage in our model is consistent with an established temporal cascade in which epithelial damage acts as the upstream trigger for eosinophilic inflammation.

Notably, AAS-induced epithelial cell death and eosinophilic infiltration were most prominent in mice with ATB-induced dysbiosis, although AAS alone also induced detectable epithelial injury, particularly in the duodenum under non-dysbiotic conditions. In untreated mice, AAS caused cell death mainly in duodenal epithelial cells, which harbour the lowest bacterial density, whereas cell death was not observed in the jejunum and ileum. This preferential susceptibility of duodenal epithelial cells may be explained by the fact that the duodenum is the first intestinal segment exposed to orally administered AAS and is therefore likely to experience the highest local concentration. In addition, the relatively low density of commensal microbiota in the duodenum may limit microbiota-mediated protective effects against epithelial injury. In contrast, under dysbiotic conditions, epithelial injury extended to the jejunum, where microbial density is higher [33]. These findings suggest that commensal bacteria and their metabolites protect IECs against AAS-induced cytotoxicity. Under dysbiosis, AAS also impaired gut barrier integrity, as shown by increased FD4 leakage. Because intestinal epithelial cells are directly exposed to luminal contents, orally administered AAS can interact with these cells from the luminal side without necessarily crossing an intact epithelial barrier. Consistent with this, AAS increased intestinal permeability, as evidenced by elevated plasma FD4 levels, indicating that epithelial cell death was associated with barrier dysfunction. This disruption of barrier integrity may subsequently facilitate the penetration of luminal antigens and contribute to tissue inflammation. Because AAS-induced epithelial injury and eosinophilic infiltration were most evident in the jejunum, whereas the ileum showed antibiotic-associated villus atrophy, our analysis focused on the small intestine. Evaluation of the colon remains important; however, cytokine ELISA of both the small and large intestine showed no group-dependent differences (Supplementary Fig. 7E).

Following ATB treatment, genes related to fatty acid metabolism and peroxisome function were upregulated in IECs. Although these transcriptional changes suggest enhanced β-oxidation and peroxisomal activity, we acknowledge that our data do not directly demonstrate increased oxidative stress or causally link these pathways to AAS susceptibility. As the expression of peroxisomal membrane proteins and β-oxidation enzymes is higher in the duodenum and jejunum than in the ileum [34], these regions may be more susceptible to oxidative stress, but further validation is needed.

Furthermore, AAS-induced pyroptosis in IECs required microbial components such as LPS and LTA. Thus, under dysbiotic conditions, the jejunal epithelium, which harbours relatively high bacterial density and exhibits active β-oxidation, appears to be the most susceptible site for epithelial cell death. In our study, AVNM treatment was associated with reduced abundance of Firmicutes and Bacteroidetes in the intestinal contents, with lower levels observed in the ATBs/AAS group. These bacterial phyla are involved in polysaccharide degradation [35], consistent with the reduced predicted degradation of starch, glycogen, glucose, and xylose in the intestinal lumen. As a result, glucose-derived energy was reduced, and IECs relied more heavily on aerobic fatty acid metabolism in mitochondria, peroxisomes, and microbodies. This metabolic shift may promote mitochondrial dysfunction and oxidative stress in IECs [36, 37].

Consistent with this notion, our in vitro experiments using the antioxidants NAC and NACA showed that ROS contributes to AAS-induced epithelial injury but is not the sole driver of cell death. Both antioxidants suppressed the cleavage of caspase-1/11, IL-33, and GSDMD, but had little effect on caspase-6/8 or GSDME activation and only partially reduced cell death, indicating the involvement of both ROS-dependent and ROS-independent pathways in AAS-induced IEC pyroptosis. These findings suggest that ROS-dependent signalling explains part of the epithelial injury and that additional caspase pathways contribute to AAS-induced pyroptosis. AVNM treatment alone modestly increased epithelial vulnerability, thereby establishing an elevated baseline level of susceptibility. The marked caspase activation, gasdermin cleavage, and eosinophilic infiltration observed after AAS administration therefore reflect additional effects beyond those induced by AVNM alone.

AAS administration was associated with a reduction in Actinobacteria, including *Bifidobacterium*, in IECs from AVNM-treated mice, which are involved in microbial synthesis of haeme and cobalamin. Because our study did not directly assess micronutrient uptake or transporter function in IECs, aluminium may influence micronutrient metabolism under dysbiotic conditions, and clarifying the causal relationship will be an important direction for future studies. Nevertheless, a reduction in Actinobacteria—key contributors to microbial cofactor biosynthesis—may weaken antioxidant defences and thereby render IECs more susceptible to injury under dysbiosis [23, 38–40].

Murine IECs express TLR2 and TLR4 (Supplementary Fig. 6B), which recognize LTA from Gram-positive and LPS from Gram-negative bacteria, respectively, and are critical for IEC homoeostasis [41]. In CI-IECs, AAS-induced pyroptotic responses were most evident under combined LPS and LTA priming conditions. Inflammasome activation can occur via the canonical NLRP3–caspase-1 pathway [11] or the non-canonical caspase-11 pathway [42]. Although the cleavage of caspase-1 and IL-1β suggested inflammasome-related signalling, NLRP3 protein was not detected in IECs in our experimental system. Thus, the present study does not provide evidence for canonical NLRP3 inflammasome activation. Instead, our results suggest that AAS induces pyroptosis-related responses through NLRP3-independent mechanisms, potentially involving other inflammasome sensors. Consistent with this, our transcriptomic analysis of IECs from antibiotic-treated mice revealed increased expression of inflammasome-related receptor genes, although canonical *Nlrp3* expression was not detected. These findings raise the possibility that caspase-1 activation in IECs under dysbiotic conditions may be initiated by an upstream mechanism distinct from the NLRP3 pathway. Caspase-6, caspase-8, and caspase-11 were involved in GSDMD- and GSDME-mediated pyroptosis in both in vivo and in vitro systems. Caspase-6 also facilitates multiple programmed cell death pathways, including pyroptosis, apoptosis, and necroptosis (PANoptosis) [43]. AAS-induced cleavage of caspases and gasdermins was markedly enhanced in IECs from dysbiotic mice, suggesting that microbial disruption promotes this pathological pathway. Further studies are needed to elucidate the detailed signalling mechanisms, including the identity of the upstream inflammasome sensors, and to determine how microbial metabolites modulate inflammasome activation.

Aluminium ions (Al³⁺) are known to enter cells, induce mitochondrial dysfunction, increase mtROS production, and activate inflammasomes [44–46]. Although heparin can bind certain metal ions [47], its affinity for Al³⁺ is relatively low [48], and thus Al³⁺ chelation alone is unlikely to explain the robust inhibitory effects observed in our study. Heparin likely suppresses AAS-induced epithelial injury through multiple complementary mechanisms. First, heparin is known to interfere with LPS binding and non-canonical caspase-11 activation through electrostatic interactions [49, 50], consistent with our observation that caspase-11 cleavage was strongly inhibited. Second, heparin binds divalent cations, including Ca²⁺, with moderate affinity [51], and Ca²⁺ fluxes are tightly linked to activation of caspase-1/11 pathways; thus, partial Ca²⁺ sequestration may attenuate inflammasome signalling. Third, highly sulfated glycosaminoglycans can interact with death-receptor signalling complexes and modulate caspase-8 activation [52, 53], providing a plausible mechanism for the reduced caspase-8 and GSDME cleavage observed in our experiments. Finally, sulfated polysaccharides have been reported to inhibit calpain activity [54, 55], which may further suppress executioner caspases such as caspase-6. Among the concentrations tested, low-dose heparin showed marked inhibitory effects on AAS-induced caspase activation and gasdermin cleavage in CI-IECs, whereas higher-dose heparin failed to suppress gasdermin cleavage. This reduced efficacy was not due to cytotoxicity, as cell viability remained unchanged even at 200 U/mL. Because our study used calcium–heparin, high concentrations may increase extracellular Ca²⁺ load, which can promote caspase-8 activation, mitochondrial stress, and GSDME-mediated cell death, thereby counteracting the protective effects observed at lower doses. High-dose heparin similarly failed to inhibit AAS-induced IEC death in vivo (1 U/g) and in vitro (>2 U/mL) (Figs. 7, 8), supporting the notion that supra-physiological concentrations diminish its protective effects. Future studies comparing calcium–heparin with non-calcium formulations, such as sodium–heparin, will be important to determine whether the loss of protection at high doses reflects Ca²⁺-dependent rather than heparin-intrinsic effects.

Although AAS is used as a food additive in certain countries, its biological effects on the intestinal epithelium under dysbiotic conditions remain poorly understood. Our findings raise concerns about the potential impact of aluminium-based additives on intestinal health, particularly in individuals with disrupted microbiota, such as those undergoing antibiotic therapy. Given that low-dose heparin suppressed caspase activation, gasdermin cleavage, and eosinophilic inflammation, it may serve as a potential therapeutic candidate to mitigate aluminium-induced mucosal injury [56]; however, further validation is required before clinical application in inflammatory or allergic gastrointestinal disorders.

The AAS doses used in this study, corresponding to approximately 180–1800 mg/kg as AAS or 11–110 mg/kg in elemental aluminium equivalents (10⁻⁵–10⁻⁴ mol per mouse), were selected to model acute peak exposure rather than chronic dietary intake. Although precise physiological epithelial exposure levels to aluminium salts remain incompletely defined, these doses allowed us to evaluate epithelial and immune responses under dysbiotic conditions. Further studies are needed to determine the relevance of these acute exposure levels to chronic dietary aluminium intake.

## Conclusions

In summary, under dysbiotic conditions, AAS induced the cleavage of caspase-1, caspase-6, caspase-8, caspase-11, IL-33, GSDMD, and GSDME in IECs, which was associated with epithelial cell death and subsequent eosinophilic inflammation. This murine model may provide novel insights into the pathogenesis of EGIDs and other allergic conditions affecting the intestinal mucosa. Low-dose heparin effectively suppressed these processes, suggesting its therapeutic potential for protecting against aluminium-induced epithelial injury and barrier dysfunction.

### Limitations and future directions

While this study elucidates the mucosal effects of aluminium additives under dysbiotic conditions, further studies are needed to clarify the underlying mechanisms, relevance to chronic dietary exposure, and translational potential of low-dose heparin in eosinophilic gastrointestinal disorders.

## Materials and methods

### Mice

For this study, 8-week-old C57BL/6 J male mice were obtained from CLEA (Tokyo, Japan) and used for experiments at 8-12 weeks of age. The mice were maintained in microisolator cages under specific pathogen–free conditions and fed autoclaved laboratory chow and water at the Laboratory Animal Center of Nippon Medical School (Tokyo, Japan). Sample sizes were chosen on the basis of our previous studies [17] and standard practice in similar mouse models; no formal statistical power calculation was performed.

### ATB treatment and oral administration of an aluminium-containing compound

The following ATBs were used: ampicillin sodium salt (Nacalai Tesque, Tokyo, Japan), vancomycin hydrochloride (Nacalai Tesque), neomycin sulphate (Nacalai Tesque), and metronidazole (Nacalai Tesque). Mice were treated with a combination of ampicillin (1 mg/mL), vancomycin (0.5 mg/mL), neomycin (1 mg/mL), and metronidazole (0.25 mg/mL) (AVNM) or no ATBs in drinking water until the intestines were removed. The mice were then administered 10^-5^, 5 × 10^-5^, or 10^-4 ^mol AAS (AlNH₄(SO₄)₂·12H₂O; Alfa Aesar, Ward Hill, MA, USA) in PBS or PBS alone via oral gavage using a gavage needle under isoflurane anaesthesia. The administered doses of AAS corresponded to approximately 180–1800 mg/kg of AAS, or 11–110 mg/kg as elemental aluminium (Al³⁺) equivalents, based on a 25-g mouse (10⁻⁵–10⁻⁴ mol per mouse).

### Treatment of mice with heparin

Heparin calcium (5 or 25 U; Mochida Pharmaceutical Co., Ltd., Tokyo, Japan) was diluted in PBS and injected subcutaneously into mice weighing approximately 25 g (0.2 or 1 U/g) 30 min before the oral administration of AAS.

### Isolation of the eosinophil fraction from the intestine and blood

LP cells containing eosinophils were prepared as described previously [17]. Small intestines were obtained from mice, faecal material was flushed from the lumen using PBS, and the connective tissues were carefully removed. After the Peyer’s patches were removed, the intestine was cut along its length, washed with PBS, and cut into 1-cm segments. The gut segments were stirred in 50 mL of Hanks’ balanced salt solution (HBSS, Invitrogen, Carlsbad, CA, USA) with 5 mM EDTA (Nacalai Tesque) and 10 µg/mL polymyxin B (Sigma-Aldrich, St. Louis, MO, USA) at 37 °C for 20 min, washed thoroughly with PBS, and minced with scissors. The minced tissues were then stirred in 20 mL of HBSS containing 10% foetal calf serum, 2 mg/mL collagenase D (Roche, Basel, Switzerland), and 200 µg/mL DNase I (Roche) at 37 °C for 60 min, and the harvested cells were passed through a nylon mesh. Subsequently, the cells were resuspended in 30% Percoll (GE Healthcare, Uppsala, Sweden), underlain with 60% Percoll, and centrifuged at 950 × *g* for 20 min. The eosinophil fraction was collected from the 30/60% interface. For isolation of the eosinophil fraction from the blood, 250 μL of blood was collected from mice using heparinised capillaries under anaesthesia. After haemolysis, blood cells were treated with Percoll solution, and the eosinophil fraction was collected as described above. Viable cells from the intestine and blood were counted under a microscope after trypan blue staining of the fraction.

### Isolation of IECs

IECs were prepared as previously described with some modifications [17]. Small intestines were obtained from the mice, and faecal material was flushed from the lumen using PBS. The guts were everted, cut into several segments, and shaken at 200 rpm in 45 mL of HBSS with 5% foetal calf serum at 37 °C for 30 min. Cells harvested from the intestinal epithelium were passed through a 100-μm cell strainer to remove tissue debris. Subsequently, the cells were resuspended in 25% Percoll, underlain with 40% Percoll, and centrifuged at 600 × *g* for 10 min. IECs were collected from the 25/40% interface. Viable cells were counted using a microscope after trypan blue staining of the fraction.

### Flow cytometry

Fc receptors were blocked with anti–mouse CD16/CD32 (2.4G2; BioLegend, San Diego, CA, USA), and eosinophils were stained using the following antibodies (Abs) and appropriate isotype controls, which were purchased from BioLegend: anti-mouse CD3e (145-2C11), CD19 (6D5), CD45 (30-F11), CD45R/B220 (RA3-6B2), NK1.1 (PK136), I-A/I-E (M5/114.15.2), CD170 (Siglec-F; S17007L), and CD193 (CCR3; J073E5). IECs were stained with anti-mouse CD45 (30-F11), CD326 (EpCAM; G8.8), and F4/80 (BM8). Cell death was evaluated using Zombie NIR (BioLegend), Annexin V (BioLegend), and 7-AAD (BioLegend). MitoSOX (Thermo Fisher Scientific, Waltham, MA, USA) was used to detect mtROS. Data were acquired on an LSRFortessa X-20 (BD Biosciences, Franklin Lakes, NJ, USA) and analysed using FlowJo software v.9.9.4 (Tree Star, Ashland, OR, USA).

### Histology

The intestinal regions were defined using a modified anatomical approach based on Nakajima-Adachi et al. [57]. The duodenum was operationally defined as the proximal 4 cm distal to the gastric junction, the ileum as the distal 8 cm proximal to the caecal junction, and the jejunum as the intervening segment. Small intestines were obtained from mice, and the duodenum, jejunum, and ileum were fixed with a 10% formalin-neutral buffer solution and embedded in paraffin. Sections (4 μm thick) were de-paraffinised and stained with HE or DFS. For DFS staining, the sections were incubated with DFS stain solution (Muto Pure Chemicals, Tokyo, Japan) for 60 min at 50 °C. After washing with water, nuclei were stained with Mayer’s haematoxylin, and the sections underwent 70–100% ethanol dehydration, were cleared with xylene, and mounted on a coverslip. Images were acquired using the Slideview VS200 microscope (Olympus, Tokyo, Japan). The number of eosinophils per high-power field (HPF) was counted at five sites per tissue using QuPath software [58] v.0.6.0, and the average number was used. Eosinophils were identified by their bilobed nuclei and strongly eosinophilic cytoplasmic granules in HE staining, and by the selective red staining of their cytoplasmic granules in DFS staining.

For immunohistochemistry, de-paraffinised jejunal sections were subjected to antigen retrieval, blocked, and incubated with primary antibodies against IL-33 (AF3626, R&D Systems, Minneapolis, MN, USA) or CCL11/eotaxin (AF-420, R&D Systems). After incubation with an HRP-conjugated secondary antibody, signals were visualised using a 3,3′-diaminobenzidine (DAB) substrate. Sections were counterstained with haematoxylin, dehydrated, cleared, and mounted. Images were acquired using a Slideview VS200 microscope. DAB-positive cells were quantified using QuPath software, and the number of IL-33-positive or CCL11-positive cells was calculated as cells per mm² in jejunal sections.

### RNA-seq and data analysis

The IEC fraction was isolated from the murine small intestine as described above and stained with 7-AAD and anti-mouse CD45, EpCAM, and F4/80 Ab. Viable 7-AAD^-^ EpCAM^+^ CD45^-^ F4/80^-^ IECs were sorted using a FACSAria II (BD Biosciences). RNA was extracted using the RNeasy Mini Kit and RNeasy MinElute Cleanup Kit (Qiagen, Venlo, the Netherlands), according to the manufacturer’s instructions. RNA quality was confirmed using a Bioanalyzer (Agilent Technologies, Santa Clara, CA, USA). Sequencing libraries were prepared using 30–100 ng of total RNA and the NEBNext Ultra II RNA Library Prep Kit for Illumina (New England Biolabs, Ipswich, MA, USA), and their concentrations and average lengths were measured on a Bioanalyzer using the Agilent DNA High Sensitivity Kit (Agilent Technologies). Additionally, qPCR measurements were performed using the Kapa Library Quantification Kit (Kapa Biosystems, Wilmington, MA, USA) to obtain accurate concentrations. Sequencing was performed on a NextSeq (Illumina, San Diego, CA, USA) using the NextSeq 500/550 High Output Kit v.2.5 (75 cycles) (Illumina) with recipe PE_75_6, and base calls were performed using RTA v.2.4.11. Sequencing data were generated in FASTQ format using bcl2fastq v.2.18.0.12. The mouse (*Mus musculus*) genome sequence (GRCm39) and the corresponding annotation information (GFF3 format) were obtained from the NCBI Genome database. Clean read data with adaptors and the first 15 bases removed were mapped to the genome sequence obtained as the reference genome using the CLC Genomics Workbench 21 (Qiagen). A generalised linear model test was used to select DEGs among the groups. RNA-seq data analyses and visualisation were conducted using R v.4.1.2. Expression change analysis was conducted using the R “DESeq2” package. Differentially expressed genes (DEGs) were identified based on a fold change ≥ 1.5 or ≤ –1.5 and an adjusted p-value (padj) ≤ 0.05. Dot plots, bubble plots, and heat maps were visualised using the R packages “ggplot2”, “clusterProfiler”, and “gplots”, respectively.

### Western blotting

CD45^-^ IECs were purified from the IEC fraction isolated from the mouse small intestine using Percoll, as described above, by removing CD45^+^ cells using a biotin anti-CD45 antibody (BioLegend) and MojoSort streptavidin nanobeads (BioLegend). BMDMs and J774.1 cells were included as positive controls for NLRP3 expression. The cells were lysed in a lysis solution consisting of 50 mM Tris-HCl, 250 mM NaCl, 5 mM EDTA, 1% NP-40, 1 mM dithiothreitol, a protease inhibitor cocktail (Nacalai Tesque), and a phosphatase inhibitor cocktail (Nacalai Tesque) for 30 min on ice and centrifuged at 20,000 × *g* at 4 °C for 2 min. Supernatants were stored at −80 °C until further use. Samples were mixed with sodium dodecyl sulphate (SDS), 40 mM dithiothreitol, and bromothymol blue, heated at 95 °C for 5 min, then subjected to 12% SDS–polyacrylamide gel electrophoresis, and transferred to polyvinylidene difluoride membranes (Immobilon-P, Millipore, Bedford, MA, USA). The membranes were blocked for 2 h at room temperature (RT; approximately 24 °C) with 5% skim milk (Morinaga Milk Co., Tokyo, Japan) in PBS with Tween 20 (0.05%) and incubated with the primary antibodies—anti-β-actin (2F1-1, BioLegend), anti-caspase-1 (5B10, BioLegend), anti-caspase-3 (#9662, Cell Signalling Technology, Danvers, MA, USA), anti-caspase-6 (#9762, Cell Signalling Technology), anti-caspase-8 (#4790 and #8592, Cell Signalling Technology), anti-caspase-9 (#9509, Cell Signalling Technology), anti-caspase-11 (EPR18628, Abcam, Cambridge, UK), anti-IL-1β (#AF401-NA, R&D Systems), anti-IL-18 (EPR19956, Abcam), anti-IL-33 (AF3626, R&D Systems), anti-GSDMD (#93709 and #39754 T, Cell Signalling Technology), anti-GSDME (#88874, Cell Signalling Technology), and anti-NLRP3 (#15101, Cell Signalling Technology)—in signal enhancer HIKARI solution A (Nacalai Tesque) at 4 °C overnight. The membranes were then incubated with horseradish peroxidase–conjugated secondary antibody in signal enhancer HIKARI solution B (Nacalai Tesque) for 1 h at RT. Membranes were visualised using Chemi-Lumi One Super (Nacalai Tesque). Data were acquired using Amersham ImageQuant 800 (Cytiva, Tokyo, Japan). After detection, membranes were stripped using WB Stripping Solution Strong (Nacalai Tesque) and reprobed for β-actin as a loading control. When necessary, membranes were also reprobed with other primary antibodies from different host species and/or antibodies detecting proteins at non-overlapping molecular weights to avoid signal interference. Band intensities were quantified relative to β-actin using ImageJ2 software (v.2.16.0), and background intensity for each lane was subtracted prior to normalisation.

### 16S rRNA amplicon analysis

Intestinal contents were collected from the murine small intestine and stored at −80 °C until use. Samples were homogenised in CD1 solution (QIAamp PowerFecal Pro DNA Kit, Qiagen) with SK38 beads using a Precellys 24 homogeniser (Bertin Technologies, Montigny-le-Bretonneux, France). DNA was extracted using the QIAamp PowerFecal Pro DNA Kit (Qiagen) according to the manufacturer’s instructions. The IEC fraction was isolated from the murine small intestine, and viable 7-AAD^-^ EpCAM^+^ CD45^-^ F4/80^-^ IECs were sorted as described above and stored at −80 °C until use. Sorted IECs were lysed in AUT solution (QIAamp UCP DNA Micro Kit, Qiagen) with reagent DX and proteinase K (Qiagen) at 56 °C for 10 min and homogenised in ZR BashingBeads tubes (Zymo Research, Irvine, CA, USA) using a Precellys 24 homogeniser. DNA was extracted using a QIAamp UCP DNA Micro Kit (Qiagen) according to the manufacturer’s instructions. DNA was extracted from the ZymoBIOMICS gut microbiome standard (Zymo Research) using the QIAamp PowerFecal Pro DNA Kit and QIAamp UCP DNA Micro Kit in the same manner and was used as a control. Extracted DNA samples were resuspended in Tris-EDTA buffer. The 16S rRNA gene sequence tags corresponding to the hypervariable V3–V4 region were generated using the MiSeq sequencing platform (Illumina). The full-length primer sequences [59] are: 16S amplicon PCR forward primer = 5′-TCGTCGGCAGCGTCAGATGTGTATAAGAGACAGCCTACGGGNGGCWGCAG;

16S amplicon PCR reverse primer = 5′-GTCTCGTGGGCTCGGAGATGTGTATAAGAGACAGGACTACHVGGGTATCTAATCC. Raw reads from Illumina MiSeq were analysed using QIIME2. To enable compositional data analysis, count data were centred log-ratio (CLR)-transformed prior to statistical comparisons. The KEGG Orthology functional profile of each sample was predicted from the 16S rRNA amplicon data using PICRUSt2.

### Cell culture and chemicals

Immortalised mouse IECs, CI-muADINTESTI (CI-IECs, InSCREENeX GmbH, Braunschweig, Germany), were cultured in muADINTESTI medium (InSCREENeX GmbH) containing 2% FBS using dishes or plates coated with epithelial cell coating solution (InSCREENeX GmbH) at 37 °C in 5% CO_2_. For stimulation experiments, cells were seeded two days before harvest to ensure a sub-confluent monolayer at the time of treatment. Immediately before LPS and LTA priming, the culture medium was replaced with DMEM/Ham’s F-12. CI-IECs were primed with 10 μg/mL LPS (from *Escherichia coli* O111:B4, Sigma-Aldrich) and 10 μg/mL LTA (from *Staphylococcus aureus*, Sigma-Aldrich) for 4 h, followed by incubation with 1 mg/mL AAS for 4 h. For antioxidant experiments, 4 mM NACA (Selleck Chemicals, Houston, TX, USA) or 5–10 mM NAC (Sigma-Aldrich) was added 90 min before AAS stimulation. For heparin treatment, 0.1–10 U/mL of heparin calcium (Mochida Pharmaceutical Co., Ltd.) was added 15 min before AAS stimulation. Harvested cells were used for flow cytometry, and cell lysates were used for western blotting.

### Plasma FITC–dextran (FD4) leakage assay

Intestinal permeability was assessed by a plasma FITC–dextran leakage assay. Mice were fasted for 4 h with free access to water, and then orally gavaged with FITC–dextran (4 kDa; Sigma-Aldrich) (15 mg per mouse in 0.25 mL PBS). Blood was collected from the retro-orbital sinus under anaesthesia using heparin-coated microcapillary tubes at 0 and 15 min after FD4 administration. Samples were centrifuged at 2000 × *g* for 10 min at 4 °C to obtain plasma. Plasma FITC fluorescence was measured using a Varioskan LUX microplate reader (Thermo Fisher Scientific) with excitation at 485 nm and emission at 528 nm. FD4 concentrations were calculated from standard curves generated using pooled mouse plasma spiked with known concentrations of FD4.

### Statistical analysis

Data are presented as mean ± standard error of the mean (SEM). Two-group comparisons were performed using the Mann–Whitney U test. For comparisons involving more than two groups, one-way analysis of variance (ANOVA) followed by Tukey’s multiple comparisons test, or the Kruskal–Wallis test followed by Dunn’s multiple comparisons test for non-parametric data, was used. Statistical analyses were performed using Prism v.9.5.1 (GraphPad Software, Boston, MA, USA). Statistical significance was set at *P* < 0.05. These post hoc tests were used for individual group comparisons, including direct comparisons between the ATB and ATB + AAS groups.

## Supplementary information

Supplementary Information

Uncropped WB Images

## Data Availability

The RNA-seq and 16S rRNA amplicon data are deposited in the DNA Data Bank of Japan under BioProject accession number PRJDB20311. The DRR Run accession numbers are DRR641247–DRR641292. All other datasets generated and analysed in this study are provided within the manuscript and the accompanying supplementary figures or can be obtained from the corresponding author upon reasonable request.
