# Supplementary material for: Aluminium ammonium sulphate induces epithelial pyroptosis and eosinophilic inflammation under dysbiotic conditions: ameliorative effects of heparin
Source: Cell Death Discov. 2026 Jul 1;12:378. doi: 10.1038/s41420-026-03237-1 (PMC13586182; doi:10.1038/s41420-026-03237-1)
Supplement: Supplementary file 1 [file 41420_2026_3237_MOESM1_ESM.pdf]

Supplementary Information

Supplementary Methods to: "Aluminium ammonium sulphate induces epithelial pyroptosis and eosinophilic inflammation under dysbiotic conditions: ameliorative effects of heparin"

Oral administration of aluminium salts

C57BL/6J mice were administered 10^{-5} mol aluminium ammonium sulphate (AAS) or potassium aluminium sulphate (PAS) ($KAl(SO_4)_2 \cdot 12H_2O$; FUJIFILM Wako Pure Chemical Co., Osaka, Japan) in PBS or PBS alone via oral gavage using an oral sonde under isoflurane anaesthesia.

Cell culture and chemicals

Immortalised mouse intestinal epithelial cells (IECs), CI-muADINTESTI (CI-IECs) were primed with LPS and LTA and stimulated with AAS, as described in the Methods section. For antibiotics (ATB) treatment, CI-IECs were treated with or without 500 μg/mL of an ATB mixture (ampicillin: neomycin: vancomycin: metronidazole, 4:4:2:1) for 14 h before priming with lipopolysaccharide (LPS) and lipoteichoic acid (LTA). For belnacasan treatment, 1.25–10 μM belnacasan (TargetMol, MA, USA) in dimethyl sulfoxide (DMSO) or DMSO alone was added to the cells 15 min before priming with LPS and LTA. The cell lysates were used for western blot analysis.

Heparin cytotoxicity assay

CI-IECs were cultured in 24-well plates and treated with various concentrations of heparin (0.5–200 U/mL) for 24 h. Cells were harvested, stained with 7-AAD and anti-EpCAM antibody (BioLegend, CA, USA), and analysed by flow cytometry. Data represent n = 4 wells/group.

Cytokine ELISA in jejunal and colonic homogenates

Jejunal and colonic tissues were collected 16 h after the final AAS administration. Approximately 60 mg of tissue was washed in cold PBS and homogenised in 600 μL of PBS containing 0.1% Triton X-100 and protease inhibitors (Nacalai Tesque, Tokyo, Japan) using CK28 beads in a Precellys 24 homogeniser. Homogenates were centrifuged at 12,000 × g for 5 min at 4 °C, and the supernatants were collected. Concentrations of CCL11 and IL-5 were measured using Quantikine ELISA kits (R&D Systems, MN, USA), and absorbance was detected using a Varioskan LUX microplate reader (Thermo Fisher Scientific, MA, USA).

***In vivo* treatment of belnacasan**

Belnacasan (2.5 mg) was dissolved in a solution (10% DMSO, 40% polyethylene glycol 300 (PEG300), 5% Tween80, and 45% saline) and was orally administered or injected intraperitoneally into mice (100 mg/kg body weight) 30 min before oral administration of AAS.

Supplementary Figures

Supplementary Fig. 1. Aluminium salts promote eosinophilic infiltration in the murine small intestine, whereas intestinal neutrophil infiltration is not increased. A–D C57BL/6 mice were orally administered 10^{-5} mol PAS or AAS once weekly for 3 weeks. The small intestine and 250 µL of blood were collected 16 h after the final administration. **A** Viable eosinophil counts in the small intestinal lamina propria (LP) and peripheral blood, determined by trypan blue exclusion. **B** Flow cytometry gating strategy for eosinophils. Cells were stained with 7-AAD, anti-CD45, anti-MHC class II (I-A/I-E), anti-Siglec-F, anti-CCR3, and a lineage cocktail (anti-CD3, CD19, B220, NK1.1). Single 7-AAD^{-} CD45^{+} lineage^{-} cells were gated and analysed for Siglec-F^{+} MHC class II^{-} CCR3^{+} eosinophils. **C, D** Representative contour plots (**C**) and summary graphs (**D**) of Siglec-F^{+} MHC class II^{-} eosinophils in LP and blood. Data are presented as mean ± SEM (n = 4 mice/group). *$P < 0.05$ by one-way ANOVA. **E** Number of neutrophils per high-power field (HPF) in jejunal sections stained with haematoxylin and eosin (HE), following oral administration of AAS or PBS once weekly for 3 weeks under ATB or control conditions. Neutrophils were quantified in five random fields from 3 mice per group. Data are presented as mean ± SEM (n = 3 mice/group).

Supplementary Fig. 2. AAS increases epithelial cell death in the murine small intestine. A Number of viable intestinal epithelial cells (IECs) in the small intestine 16 h after oral administration of 10^{-5} mol PAS or AAS. IECs were isolated from the small intestine and counted using trypan blue exclusion. **B** Flow cytometry gating strategy for identifying dying/dead Zombie^{+} Annexin V^{+} cells. Cells were stained with Zombie, Annexin V, anti-CD45, and anti-EpCAM antibodies. Single EpCAM^{+} or CD45^{+} cells were gated, and percentages of dying/dead

Zombie^{+} Annexin V^{+} cells were analysed. **C, D** Representative flow cytometry plots (**C**) and quantification (**D**) showing the percentages of dying/dead Zombie^{+} Annexin V^{+} EpCAM^{+} or CD45^{+} cells in mice orally administered PAS or AAS. Data represent mean ± SEM (n = 4 mice/group). *P < 0.05 by Kruskal–Wallis test. **E** Number of viable IECs and percentages of dying/dead Zombie^{+} EpCAM^{+} IECs in the small intestine 1 h after oral administration of 10^{-5} mol AAS following ATB treatment. IECs were stained with Zombie, anti-CD45, and anti-EpCAM antibodies. Data are presented as mean ± SEM (n = 6–7 mice/group). *P < 0.05, **P < 0.01, ****P < 0.001 by Kruskal–Wallis test. **F** Flow cytometry gating strategy for identifying dying/dead EpCAM^{+} IECs and CD45^{+} cells. Cells were stained with 7-AAD, anti-CD45, and anti-EpCAM antibodies. Single EpCAM^{+} or CD45^{+} cells were gated, and percentages of 7-AAD^{+} dying cells were analysed.

Supplementary Fig. 3. AAS enhances *Il33* gene expression, whereas ATBs increase *Casp6* gene expression in murine IECs. Mice were administered water with or without ATBs for 2 weeks, followed by oral administration of 10^{-5} mol AAS. Small intestines were collected 1 h after AAS administration for isolation of IECs and RNA sequencing. **A** IECs were isolated from the small intestine and stained with 7-AAD, anti-CD45, anti-EpCAM, and anti-F4/80 antibodies. Viable 7-AAD^{-} EpCAM^{+} CD45^{-} F4/80^{-} IECs were gated and sorted using a FACSAria II. **B** Volcano plot and summary of the number of differentially expressed genes (DEGs) in IECs compared with untreated controls. Genes relevant to cell death and inflammation are highlighted. **C** Heatmap showing expression patterns of selected genes associated with cell death pathways and immune responses. **D** Gene ontology (GO) enrichment analysis of downregulated DEGs,

categorised by Biological Process (BP) and Cellular Component (CC). Data were obtained from 3 mice per group.

Supplementary Fig. 4. AAS induces caspase cleavage in IECs under ATB-treated conditions. **A** Immunoblot analysis of cleaved caspases and cytokines in IEC lysates at 4, 8, and 16 h after oral administration of 10^{-5} mol AAS in ATB-treated mice. Mice were administered ATBs in drinking water for 2 weeks, followed by oral AAS administration. $CD45^{-}$ IECs were isolated by negative magnetic bead selection from small intestinal tissues. Expression of cleaved caspase-1, caspase-6, caspase-11, IL-18, and IL-33 was assessed. Lysates were pooled from 5 mice per group. Representative β-actin loading controls corresponding to each western blot are shown. **B** Immunoblot analysis of cleaved caspase-9 in IEC lysates 4 h after oral administration of various doses of AAS in mice treated with or without ATBs. **C** Flow cytometry gating strategy for assessing mitochondrial reactive oxygen species (mtROS) using MitoSOX Red in viable $EpCAM^{+}$ or $CD45^{+}$ cells. Cells were stained with Zombie, anti-CD45, anti-EpCAM, and MitoSOX, and viable ($Zombie^{-}$) $EpCAM^{+}$ or $CD45^{+}$ cells were gated for analysis. **D, E** Representative histogram overlays (**D**) and summary graphs (**E**) showing MitoSOX Red fluorescence intensity in viable $CD45^{+}$ cells of the small intestine following AAS administration under ATB treatment. Blue histograms represent PBS-treated controls. Graphs show mean fluorescence intensity. Data are presented as mean ± SEM (n = 6–7 mice/group). *$P < 0.05$ by Kruskal–Wallis test.

Supplementary Fig. 5. Microbial diversity in murine IECs is greater than that in intestinal contents. Mice were administered drinking water with or without ATBs, and 2 weeks later, they received an oral dose of 10^{-5} mol AAS. The small intestine was harvested 1 h after administration. Intestinal contents (IC) were collected, and viable IECs (7-AAD^{-} EpCAM^{+} CD45^{-} F4/80^{-}) were sorted as described in Supplementary Fig. 3A. 16S rRNA amplicon sequencing was performed on both IC and IEC samples. **A, B** Chao1 index and weighted UniFrac distance from the gut microbiome standard, comparing microbiomes in IC and IECs across all mice (**A**) and by treatment group (**B**). **C** Comparison of centred log-ratio (CLR)-transformed abundance of *Candidatus* Arthromitus in IC and IECs. Data are shown as box-and-whisker plots (n = 4 mice/group). *$P < 0.05$, **$P < 0.01$, ****$P < 0.001$ by Kruskal–Wallis test for multiple comparisons or Mann–Whitney U test for pairwise comparisons.

Supplementary Fig. 6. Characterisation of CI-IECs and effects of ATB pretreatment, heparin, and belnacasan on AAS-induced responses *in vitro.* **A** Expression of CD45 and EpCAM in J774.1, murine intestinal IECs, and CI-IECs. Cells were stained with 7-AAD, anti-CD45, and anti-EpCAM antibodies. Viable 7-AAD^{-} cells were analysed by flow cytometry. **B** Immunoblot analysis of pro-caspase-1, pro-caspase-6, pro-caspase-8, pro-caspase-11, full-length IL-18 (FL-IL-18), IL-33 (FL-IL-33), GSDMD (FL-GSDMD), gasdermin E (FL-GSDME), NLRP3, TLR2, and TLR4 in lysates from J774.1, murine IECs, and CI-IECs. **C** Immunoblot analysis of cleaved caspase-1, caspase-6, caspase-11, IL-33, and GSDMD in CI-IEC lysates stimulated with 1 mg/mL AAS for 4 h after priming with 10 μg/mL LPS and LTA for 4 h, with or without prior treatment with an ATB mixture (500 μg/mL; ampicillin:neomycin:vancomycin:metronidazole = 4:4:2:1) for 14 h. **D, E** Flow cytometric

analysis of cell death after heparin treatment. CI-IECs were incubated with or without various concentrations of heparin (0.5–200 U/mL) for 24 h, stained with 7-AAD, and analysed by flow cytometry. **D** Representative plots are shown. **E** Quantification of 7-AAD$^+$ EpCAM$^+$ dead CI-IECs after heparin treatment. Data are presented as mean ± SEM (n = 4 wells/group). **F** Immunoblot analysis of cleaved caspase-1, caspase-6, caspase-11, IL-33, and GSDMD in CI-IEC lysates treated with belnacasan (1.25–10 μM) before stimulation with AAS (1 mg/mL) for 4 h after LPS/LTA priming. Representative β-actin loading controls corresponding to each western blot are shown (panels **B**, **C**, and **F**).

Supplementary Fig. 7. Belnacasan does not ameliorate pyroptotic cell death in IECs or eosinophilic infiltration in the murine small intestine. **A** Immunoblot analysis of cleaved caspase-1, caspase-11, IL-33, and GSDMD in IEC lysates from mice treated with oral or intraperitoneal (i.p.) belnacasan (beln.) or vehicle (veh.) prior to AAS administration following ATB treatment. Mice received ATBs in drinking water for 2 weeks, followed by administration of belnacasan (2.5 mg) or vehicle. Thirty minutes later, 5×10^{-5} mol AAS was administered orally. The small intestine was collected 4 h later, and CD45$^-$ IECs were isolated as described in Fig. 3C. Data are from pooled IEC lysates of five mice per group. **B** Immunoblot analysis of cleaved caspase-6, caspase-8, and GSDME in IECs from the same treatment groups as in **A**. Representative β-actin loading controls corresponding to each western blot are shown (panels **A** and **B**). **C, D** Representative flow cytometry contour plots (**C**) and summary graphs (**D**) showing the percentage of dying/dead 7-AAD$^+$ IECs. IECs were stained as described in Fig. 2**D**. Data are presented as mean ± SEM (n = 3 mice/group). **E** CCL11 and IL-5 concentrations in supernatants of jejunal and colonic tissue homogenates. Mice were treated with ATBs followed by oral

administration of AAS (10^{-5} mol) with or without injection of 5 U heparin once weekly for 3 weeks. The jejunum and colon were harvested 16 h after the final dose, and CCL11 and IL-5 levels in tissue homogenate supernatants were measured by ELISA. Data are presented as mean ± SEM (n = 4 mice/group). **F, G** Representative HE and direct fast scarlet (DFS) staining of jejunal sections (**F**) and quantification of eosinophils per HPF (**G**). Two weeks after ATB treatment, mice were i.p. injected with belnacasan or vehicle, followed by oral administration of 5×10^{-5} mol AAS once weekly for 3 weeks. Jejunal tissues were processed as in Fig. 9. Arrows indicate eosinophils. Eosinophils were counted in five random fields per sample. Data are presented as mean ± SEM (n = 3–4 mice/group).

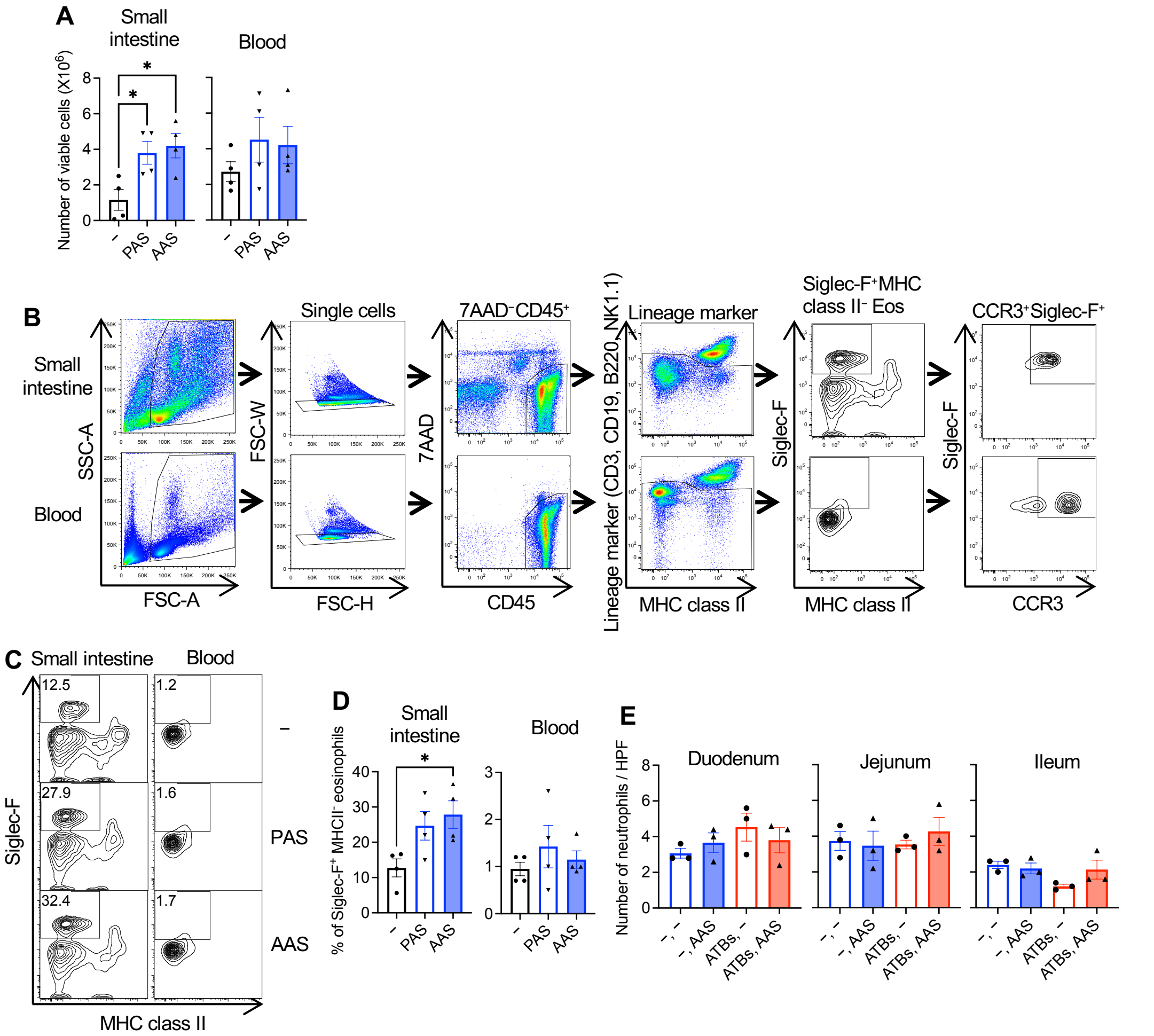

Supplementary Fig. 1

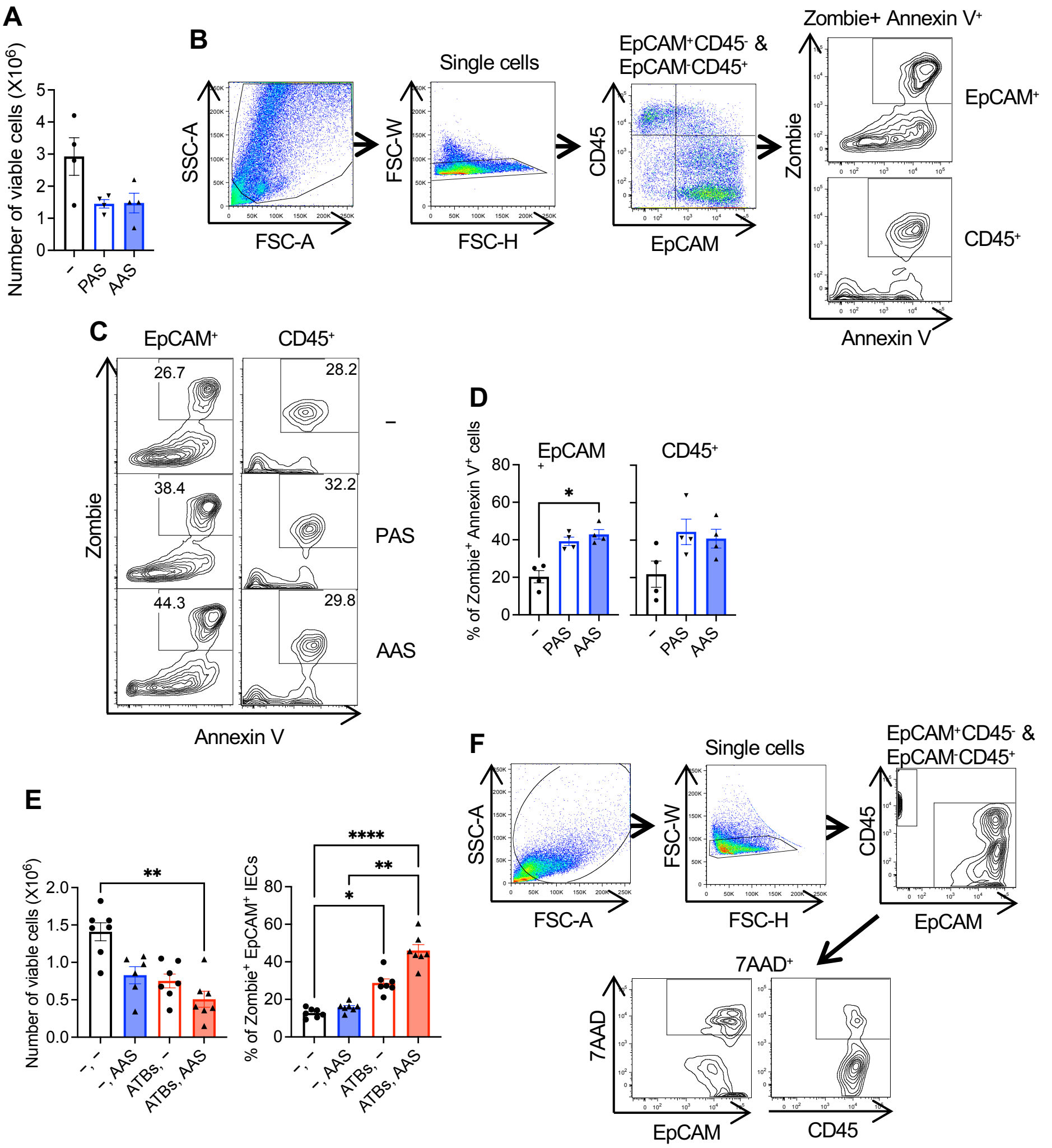

Supplementary Fig. 2

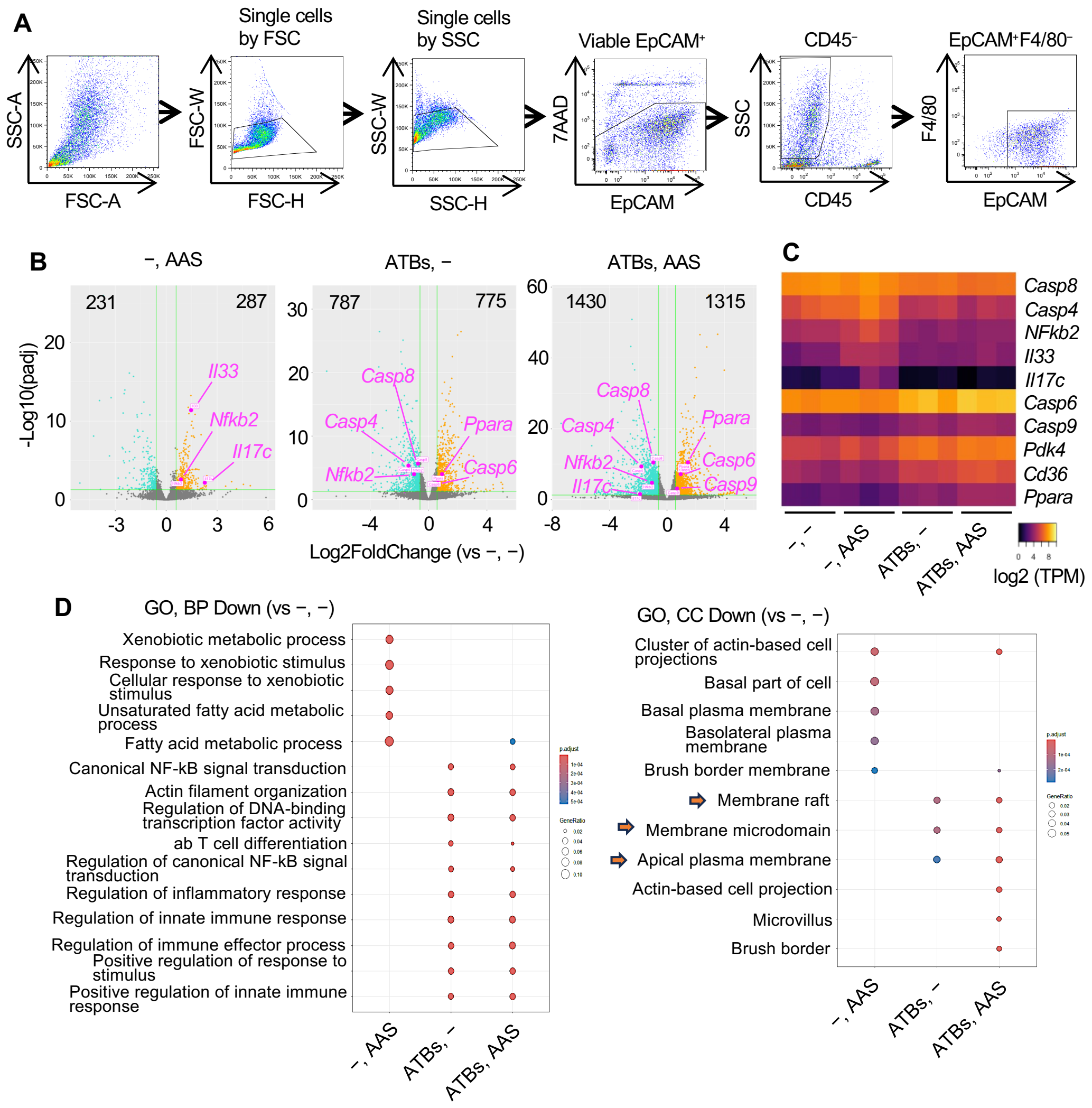

Supplementary Fig. 3

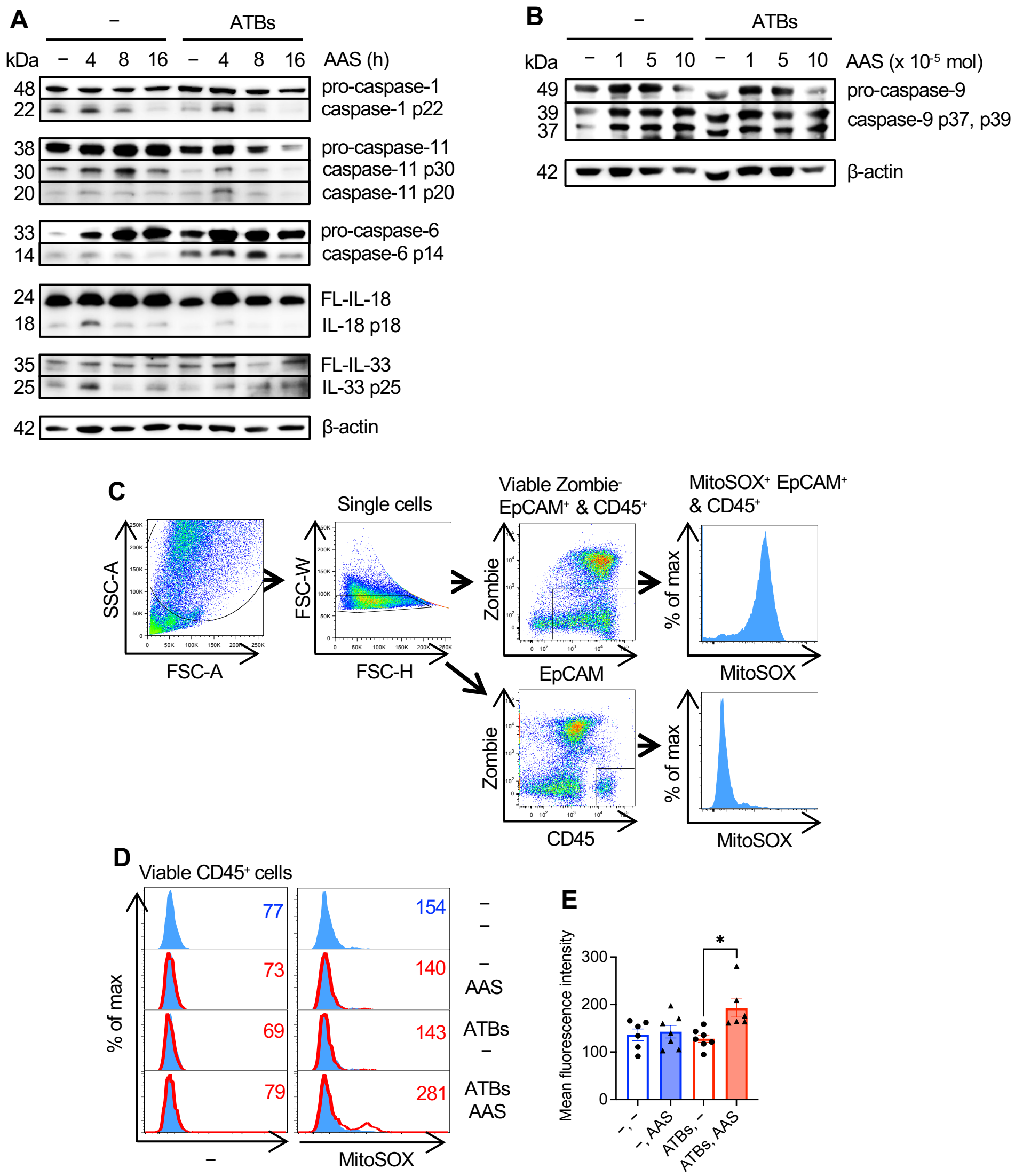

Supplementary Fig. 4

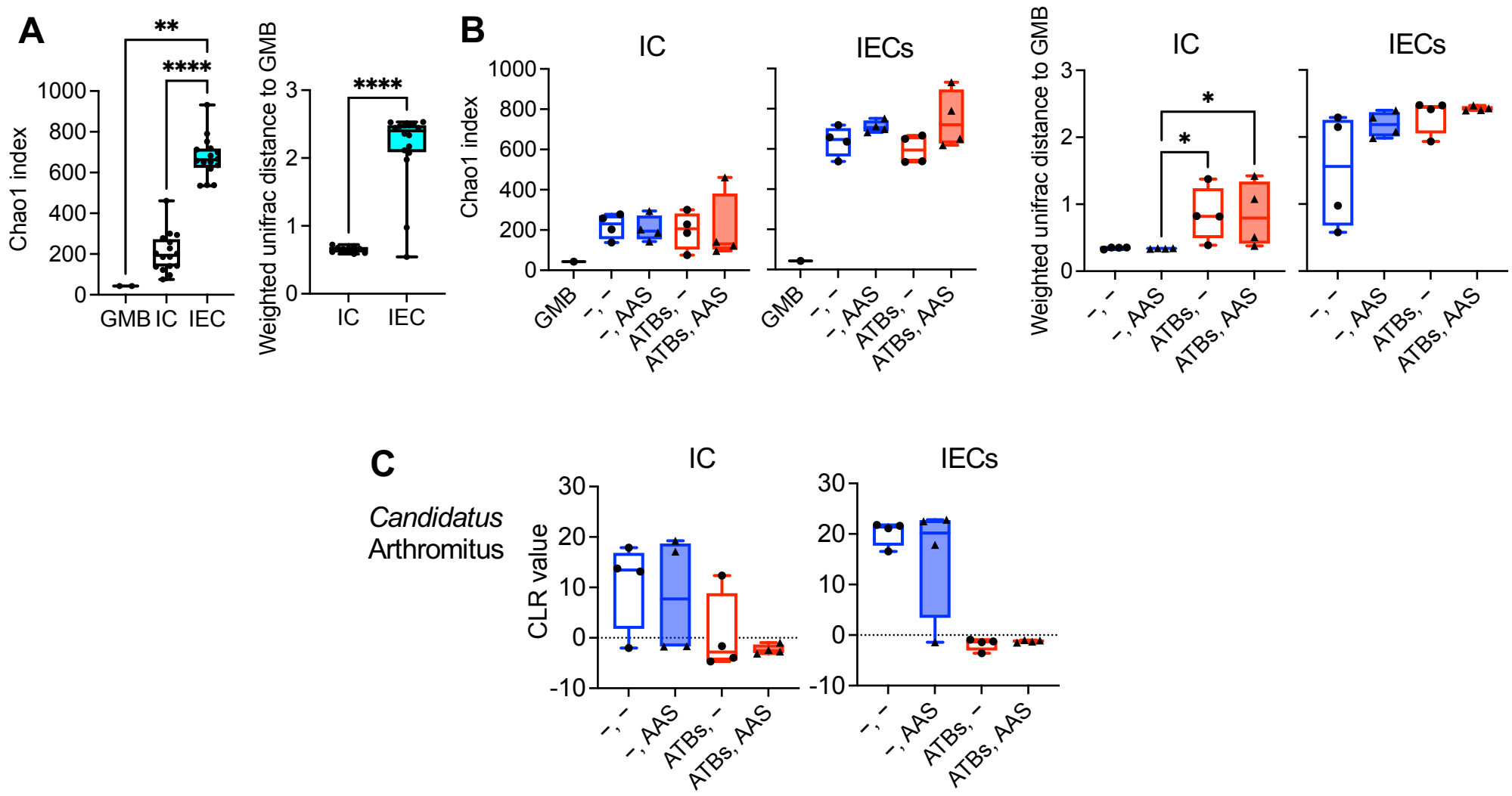

Supplementary Fig. 5

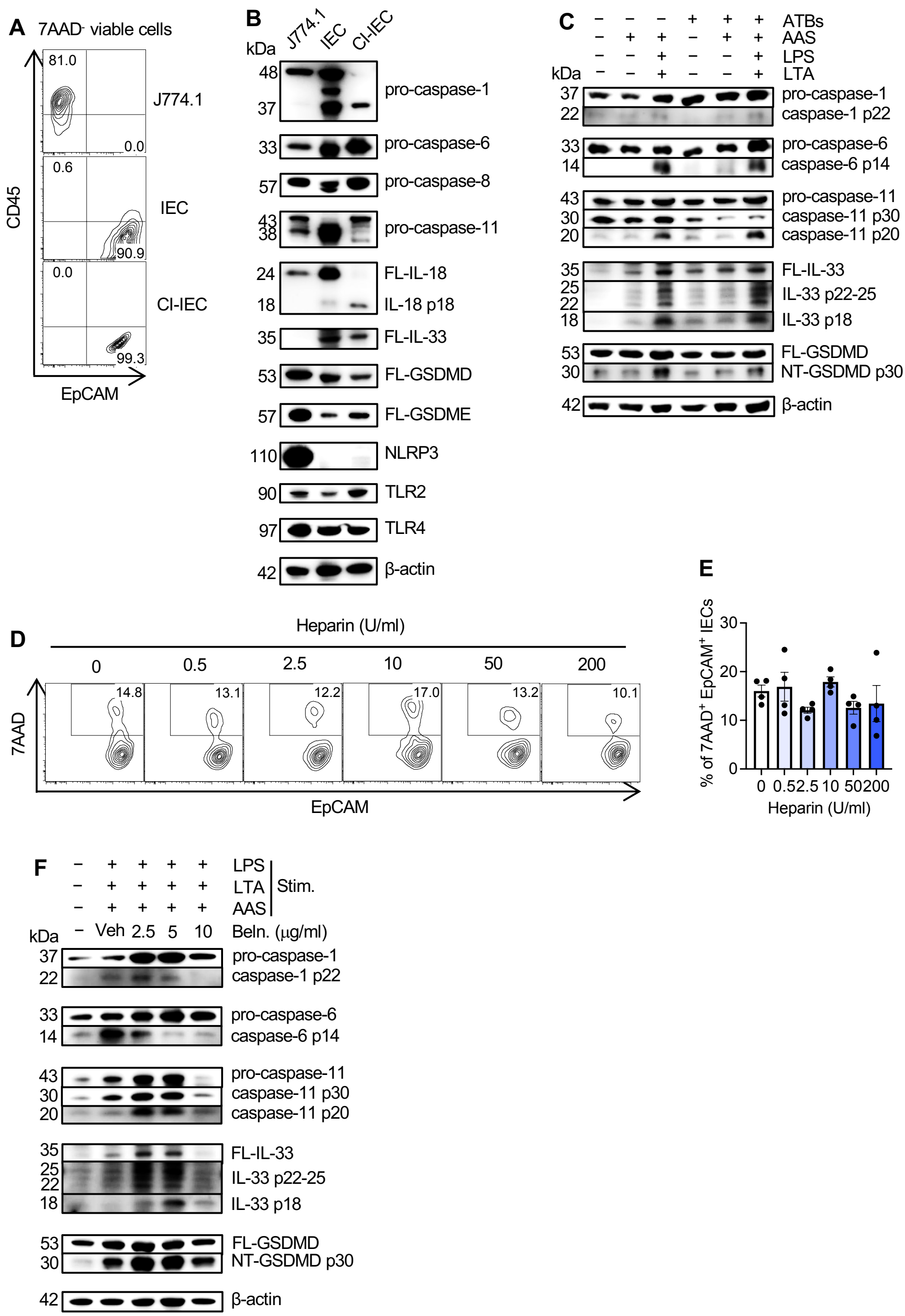

Supplementary Fig. 6

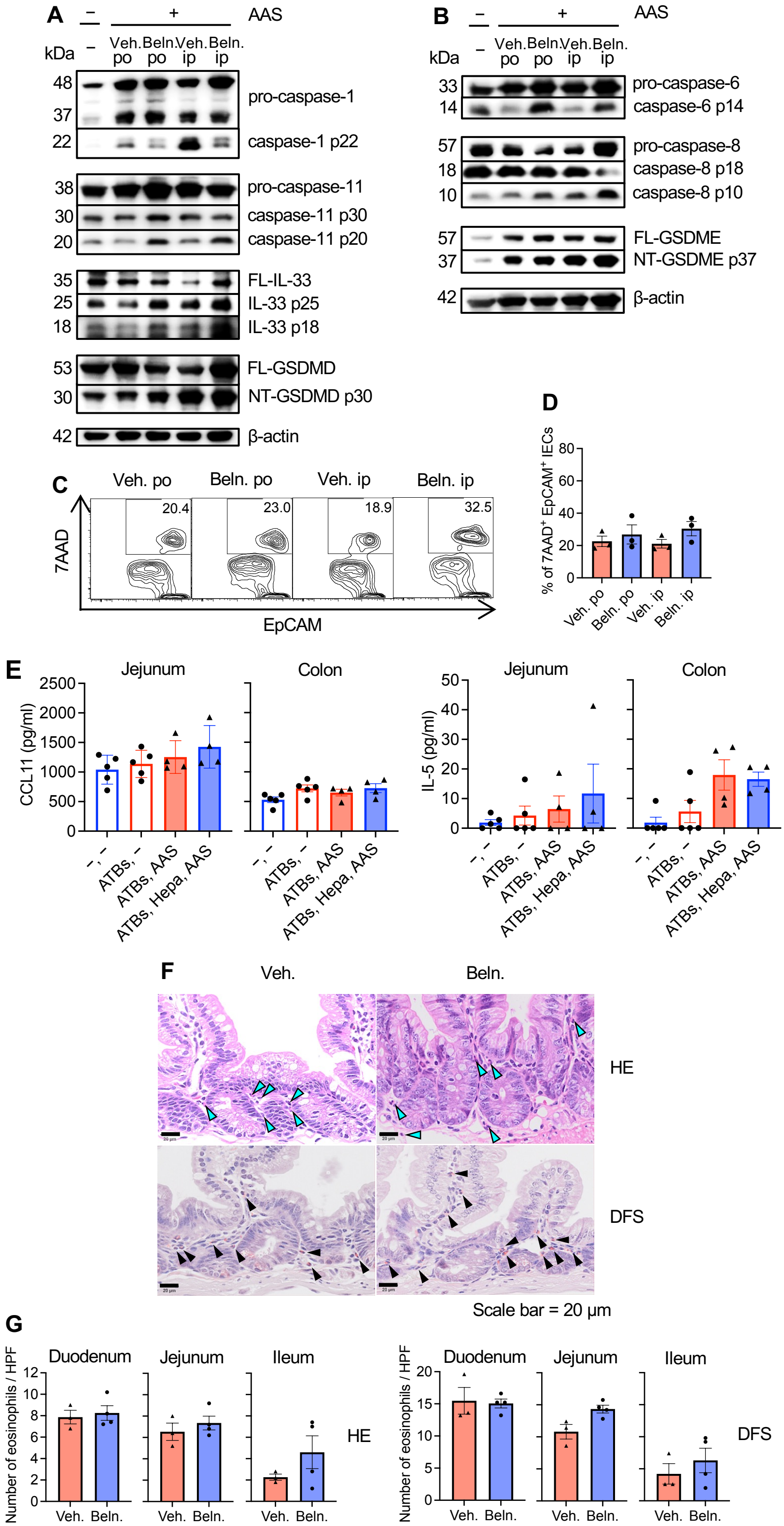

Supplementary Fig. 7
