# Supplementary material for: Aluminium ammonium sulphate induces epithelial pyroptosis and eosinophilic inflammation under dysbiotic conditions: ameliorative effects of heparin
Source: Cell Death Discov. 2026 Jul 1;12:378. doi: 10.1038/s41420-026-03237-1 (PMC13586182; doi:10.1038/s41420-026-03237-1)
Supplement: Supplementary file 2 [file 41420_2026_3237_MOESM2_ESM.pdf]

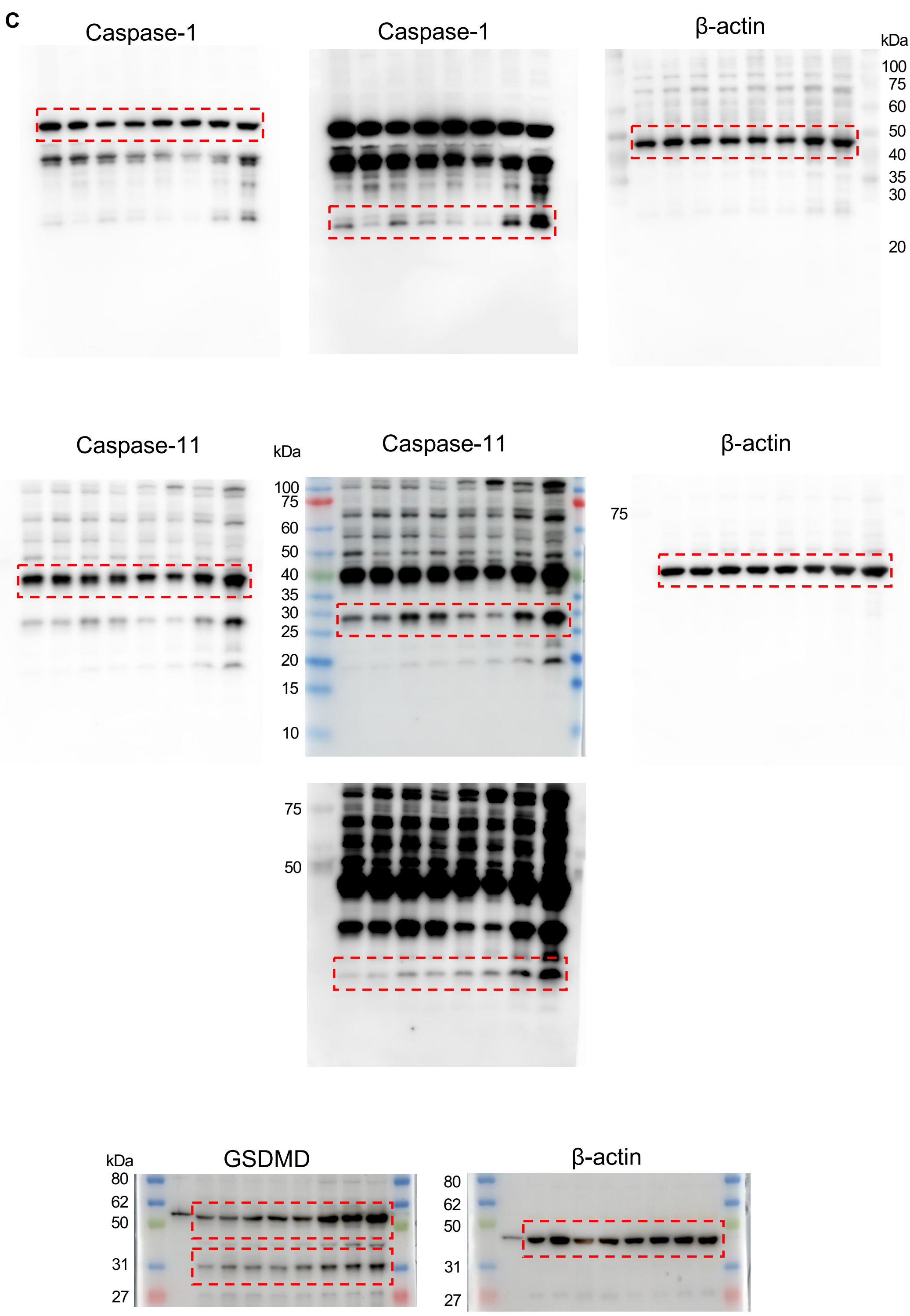
c
Caspase-1
Caspase-1
β-actin
kDa
100
75
60
50
40
35
30
20
Caspase-11
kDa
Caspase-11
100
75
60
50
40
35
30
25
20
15
10
β-actin
75
75
50
kDa
GSDMD
80
62
50
31
27
β-actin
80
62
50
31
27

Fig. 3

C

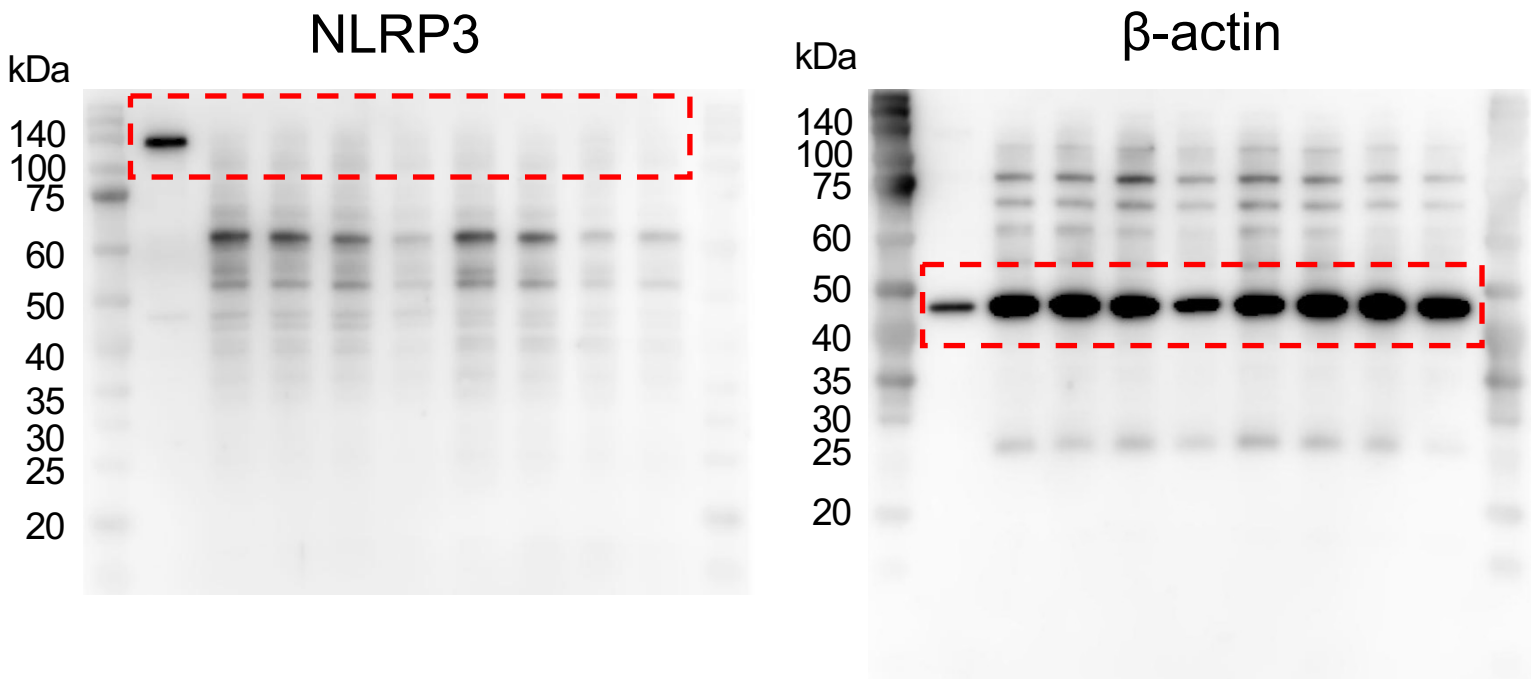
NLRP3
kDa
140
100
75
60
50
40
35
30
25
20
β-actin
kDa
140
100
75
60
50
40
35
30
25
20

Fig. 3

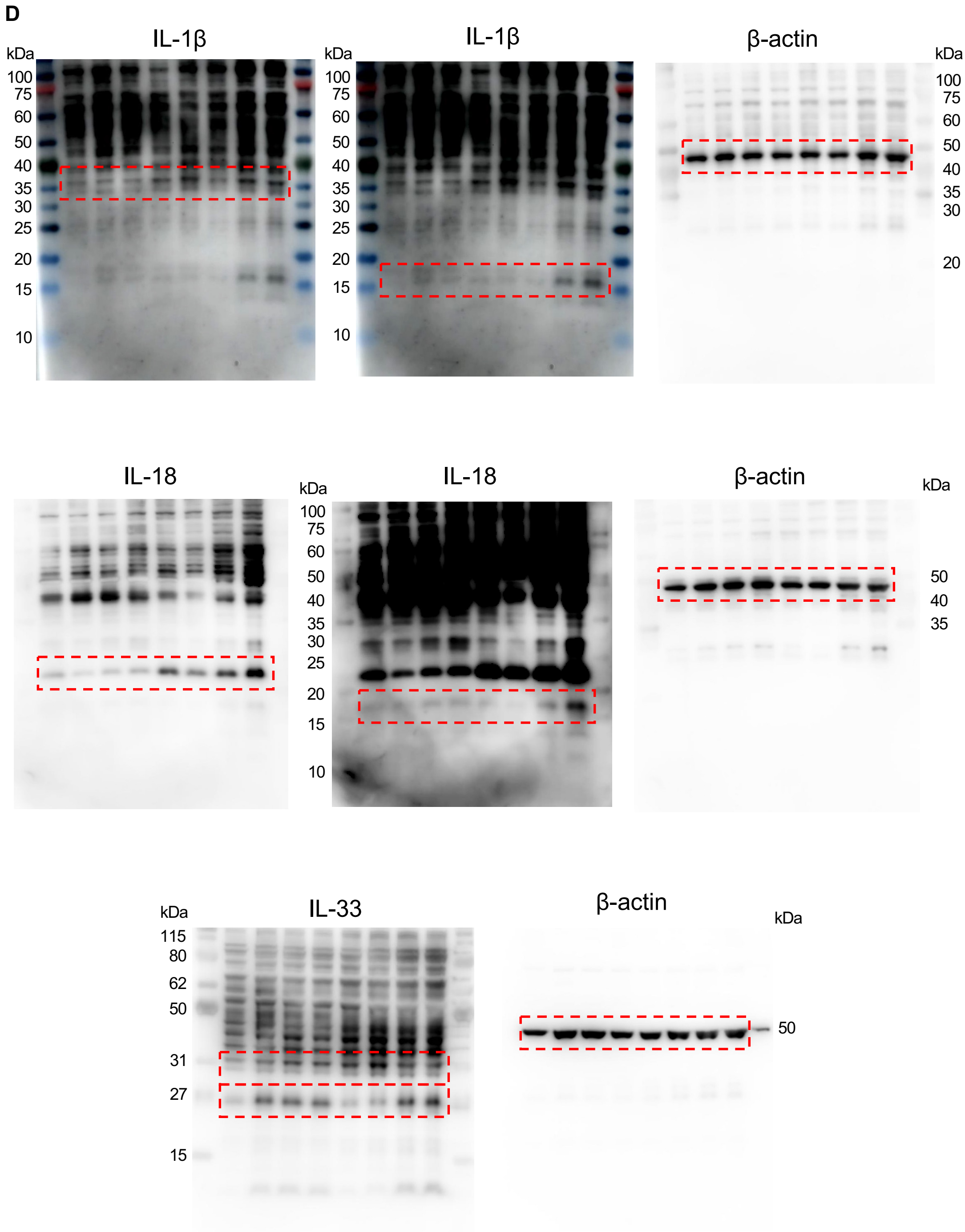
D
IL-1β
kDa
100
75
60
50
40
35
30
25
20
15
10
IL-1β
kDa
100
75
60
50
40
35
30
25
20
15
10
β-actin
kDa
100
75
60
50
40
35
30
20
IL-18
IL-18
kDa
100
75
60
50
40
35
30
25
20
15
10
β-actin
kDa
50
40
35
IL-33
kDa
115
80
62
50
31
27
15
β-actin
kDa
50

Fig. 3

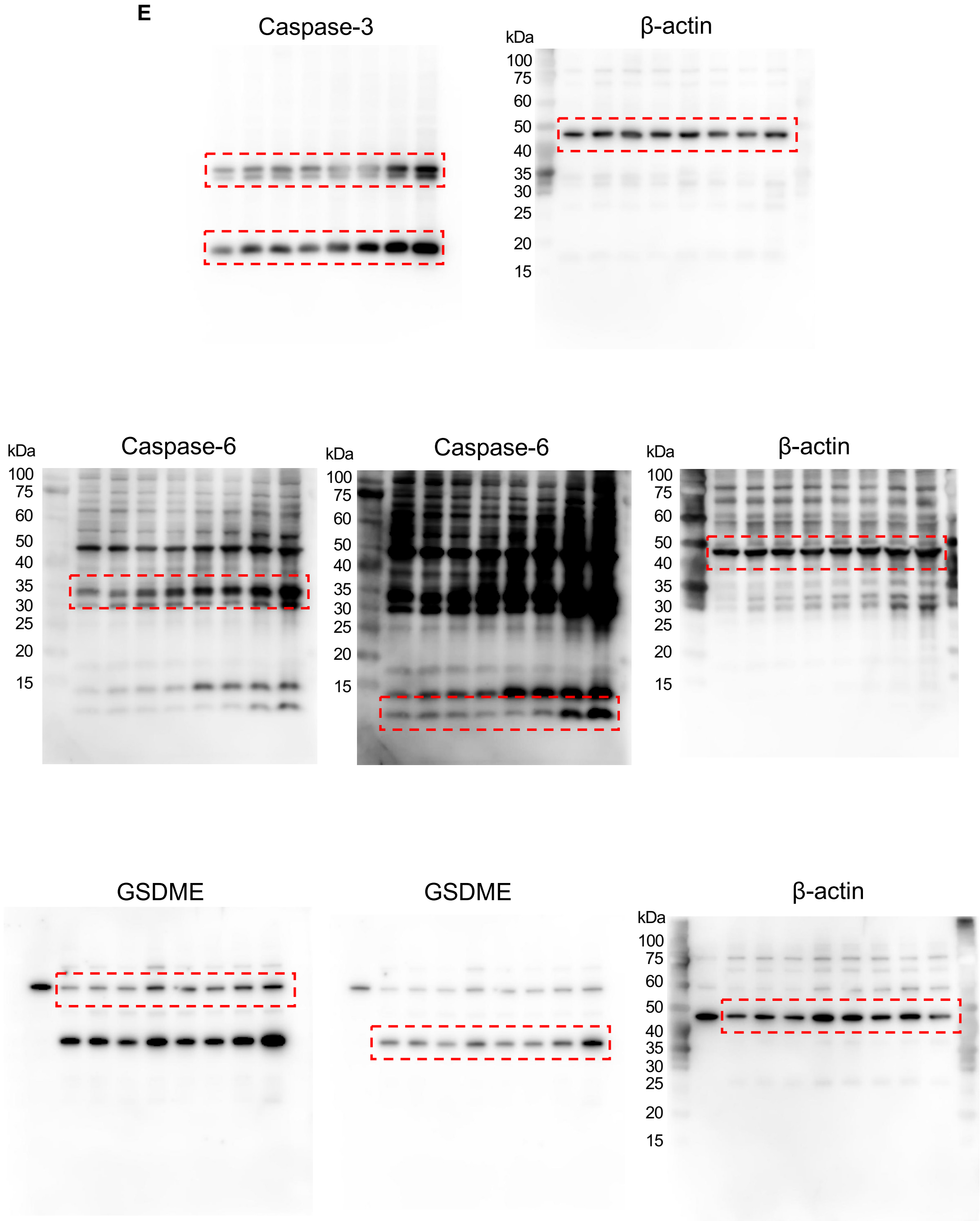
E
Caspase-3
β-actin
kDa
100
75
60
50
40
35
30
25
20
15
Caspase-6
Caspase-6
β-actin
GSDME
GSDME
β-actin

Fig. 3

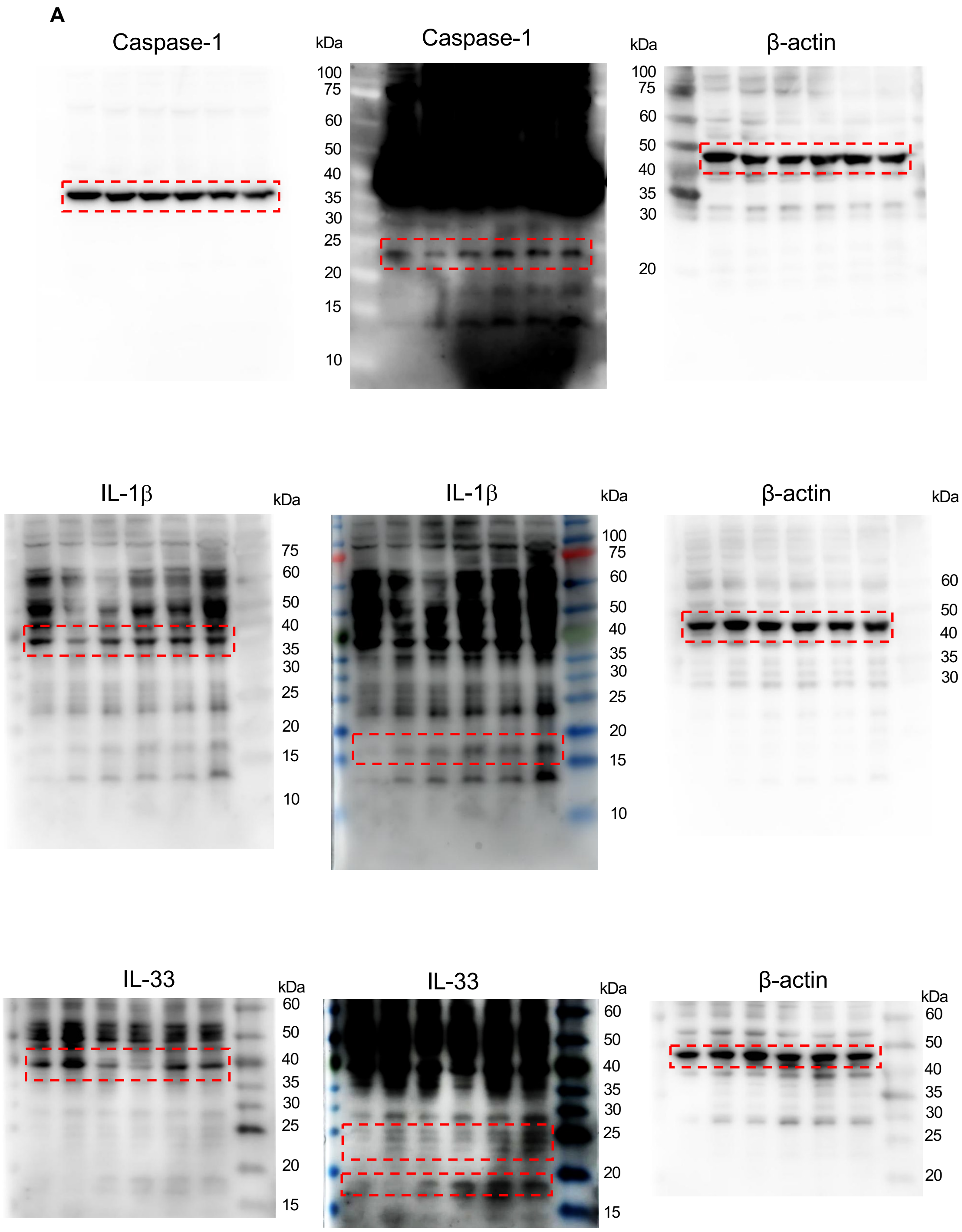
A
Caspase-1
kDa
100
75
60
50
40
35
30
25
20
15
10
Caspase-1
kDa
100
75
60
50
40
35
30
20
β-actin
IL-1β
kDa
75
60
50
40
35
30
25
20
15
10
IL-1β
kDa
100
75
60
50
40
35
30
25
20
15
10
β-actin
kDa
60
50
40
35
30
IL-33
kDa
60
50
40
35
30
25
20
15
IL-33
kDa
60
50
40
35
30
25
20
15
β-actin
kDa
60
50
40
35
30
25
20

Fig. 5

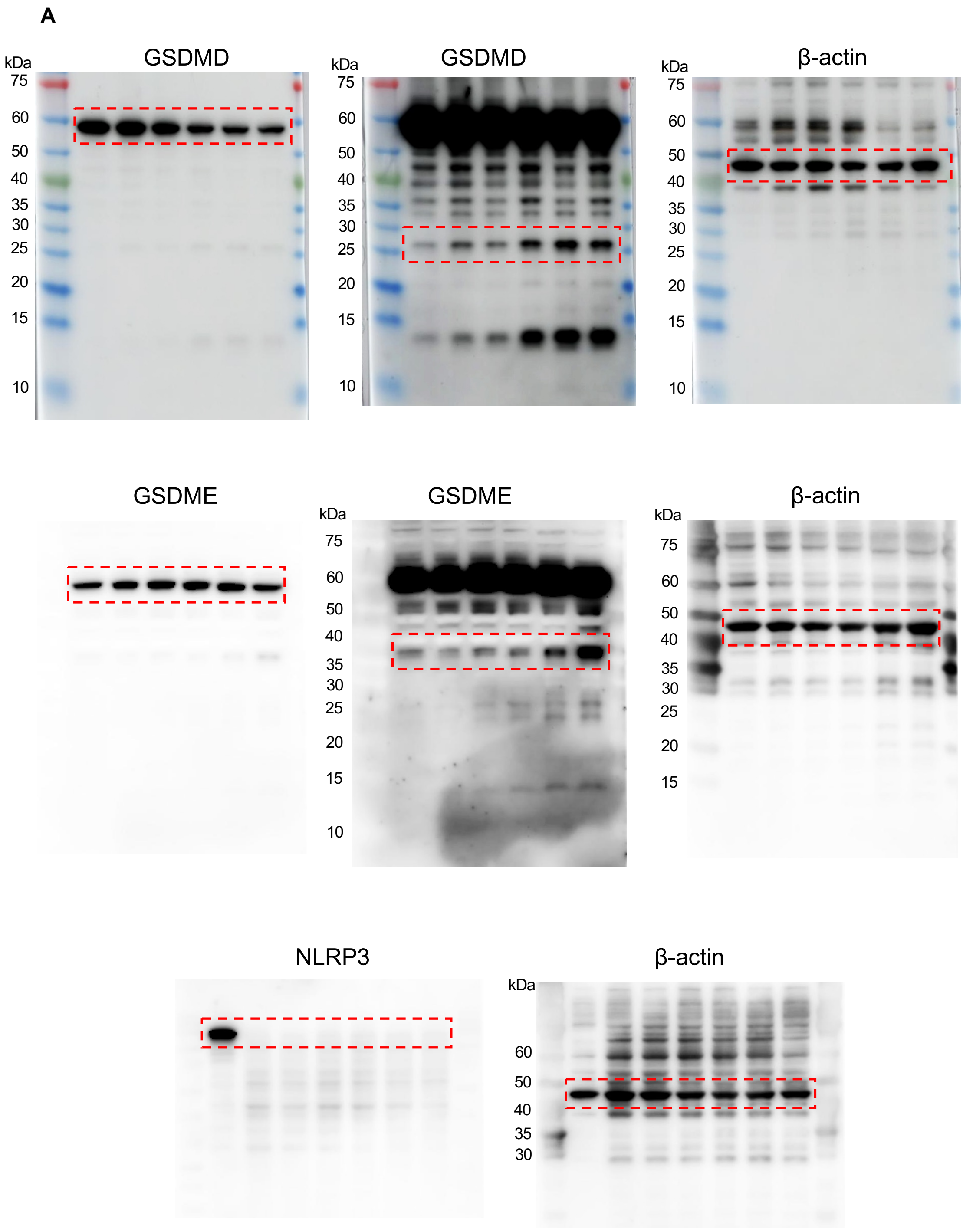
A
GSDMD
kDa
75
60
50
40
35
30
25
20
15
10
GSDMD
kDa
75
60
50
40
35
30
25
20
15
10
β-actin
kDa
75
60
50
40
35
30
25
20
15
10
GSDME
GSDME
kDa
75
60
50
40
35
30
25
20
15
10
β-actin
kDa
75
60
50
40
35
30
25
20
15
NLRP3
β-actin
kDa
60
50
40
35
30

Fig. 5

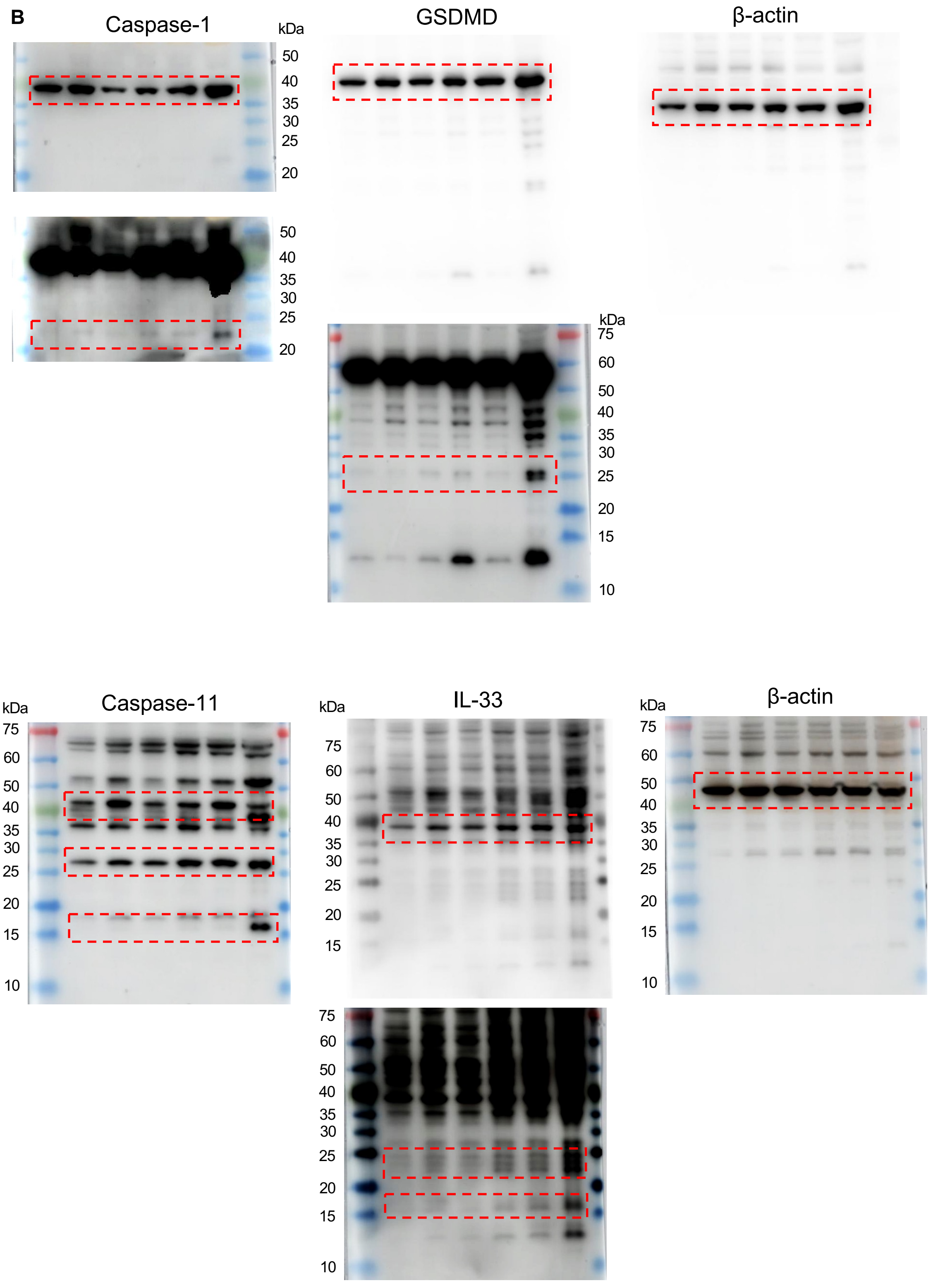
B
Caspase-1
kDa
50
40
35
30
25
20
GSDMD
β-actin
kDa
75
60
50
40
35
30
25
20
15
10
Caspase-11
IL-33
β-actin

Fig. 5

C

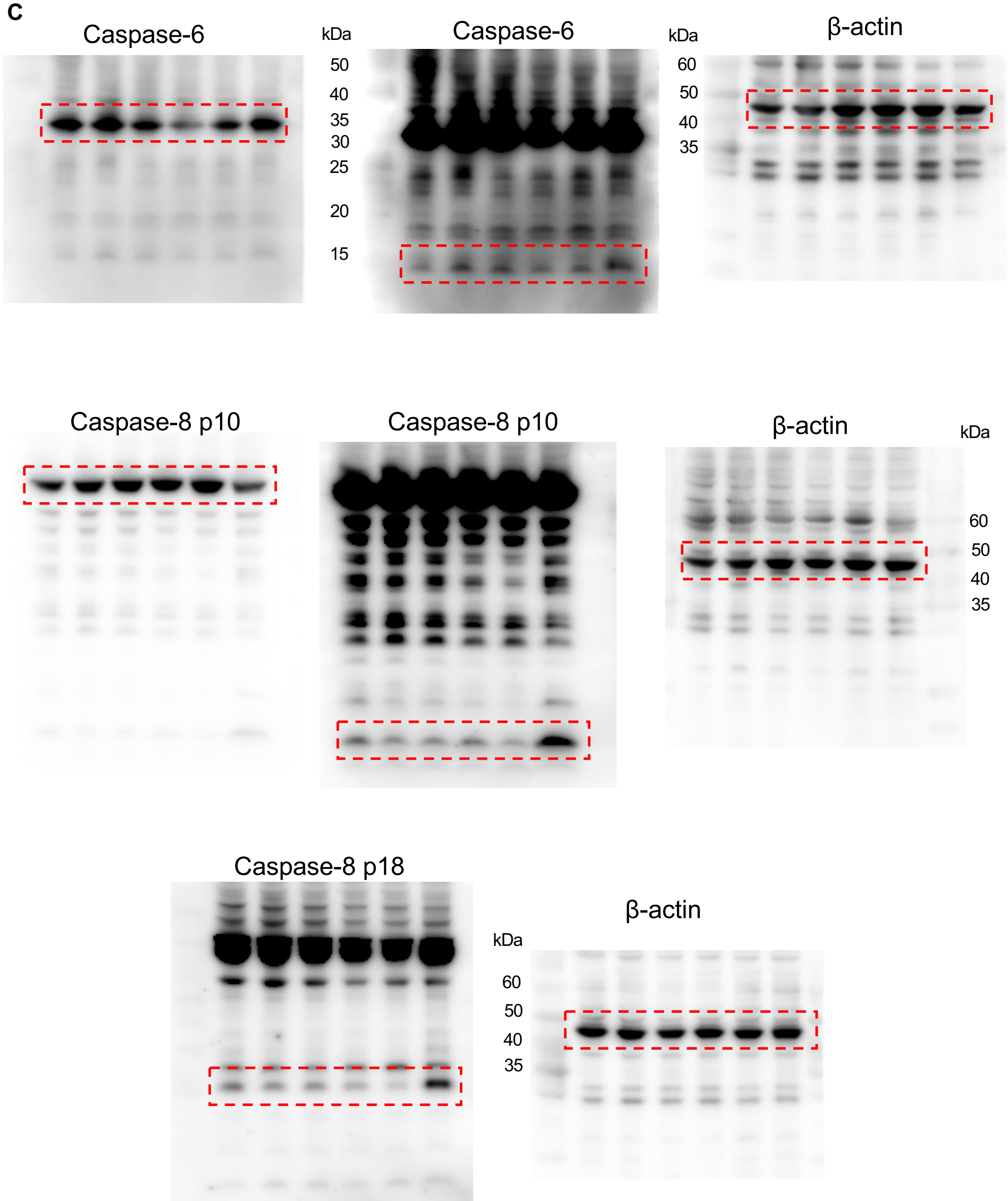
Caspase-6
kDa
50
40
35
30
25
20
15
Caspase-6
kDa
60
50
40
35
β-actin
Caspase-8 p10
Caspase-8 p10
β-actin
kDa
60
50
40
35
Caspase-8 p18
β-actin
kDa
60
50
40
35

Fig. 5

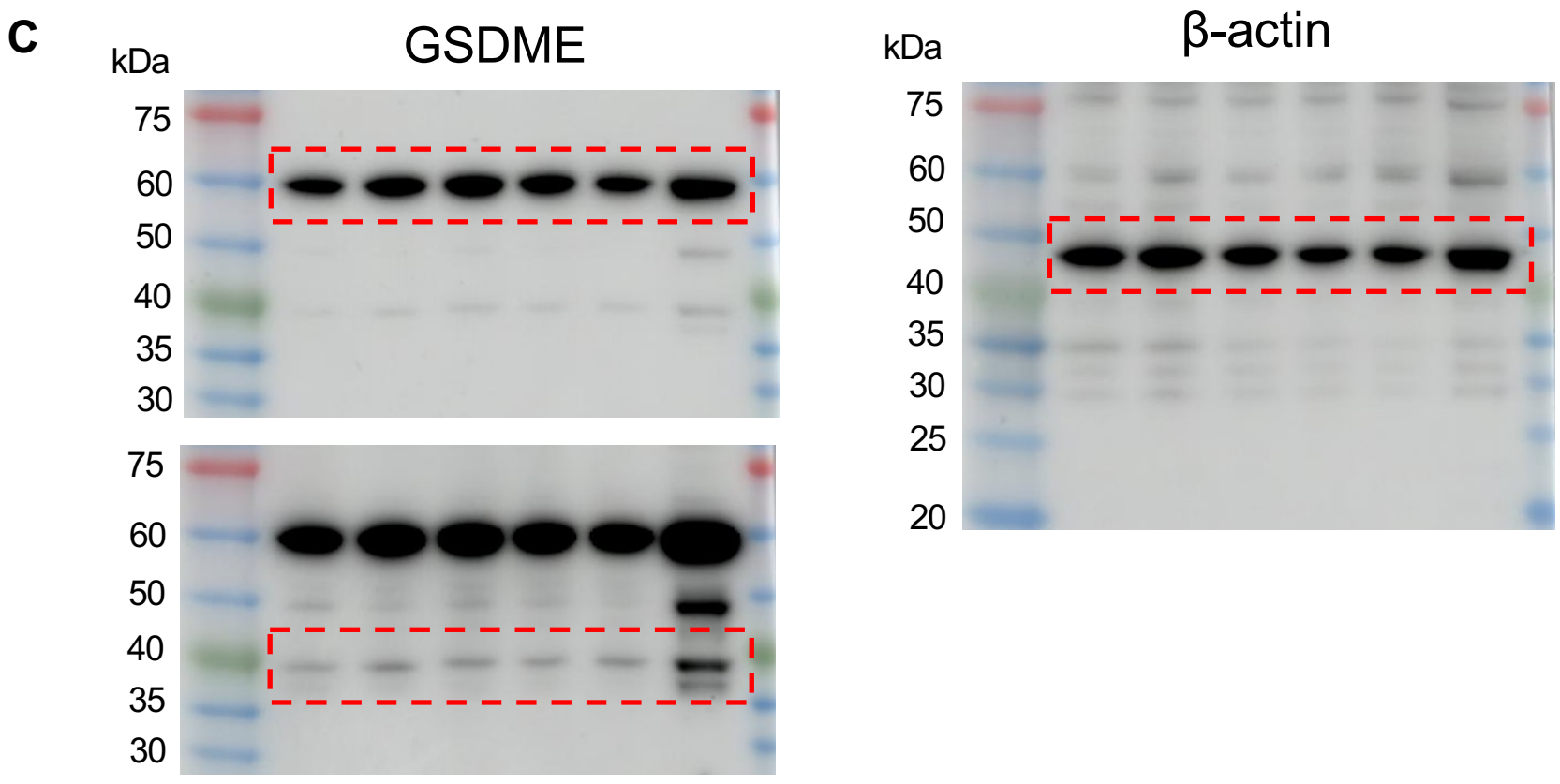
C
kDa
GSDME
75
60
50
40
35
30
75
60
50
40
35
30
kDa
β-actin
75
60
50
40
35
30
25
20

Fig. 5

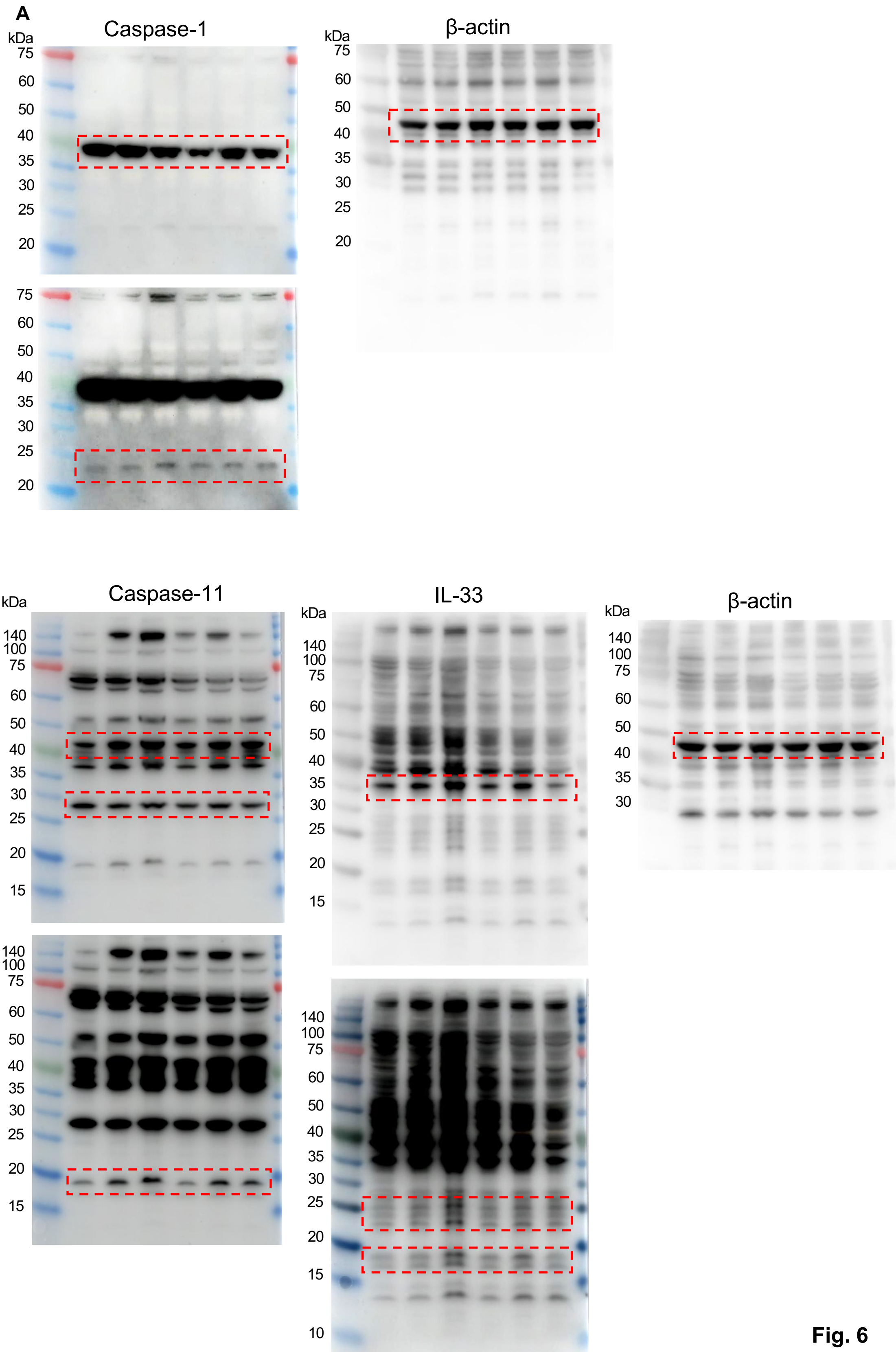

A
Caspase-1
β-actin
kDa
75
60
50
40
35
30
25
20
Caspase-11
IL-33
β-actin
140
100
15
10

Fig. 6

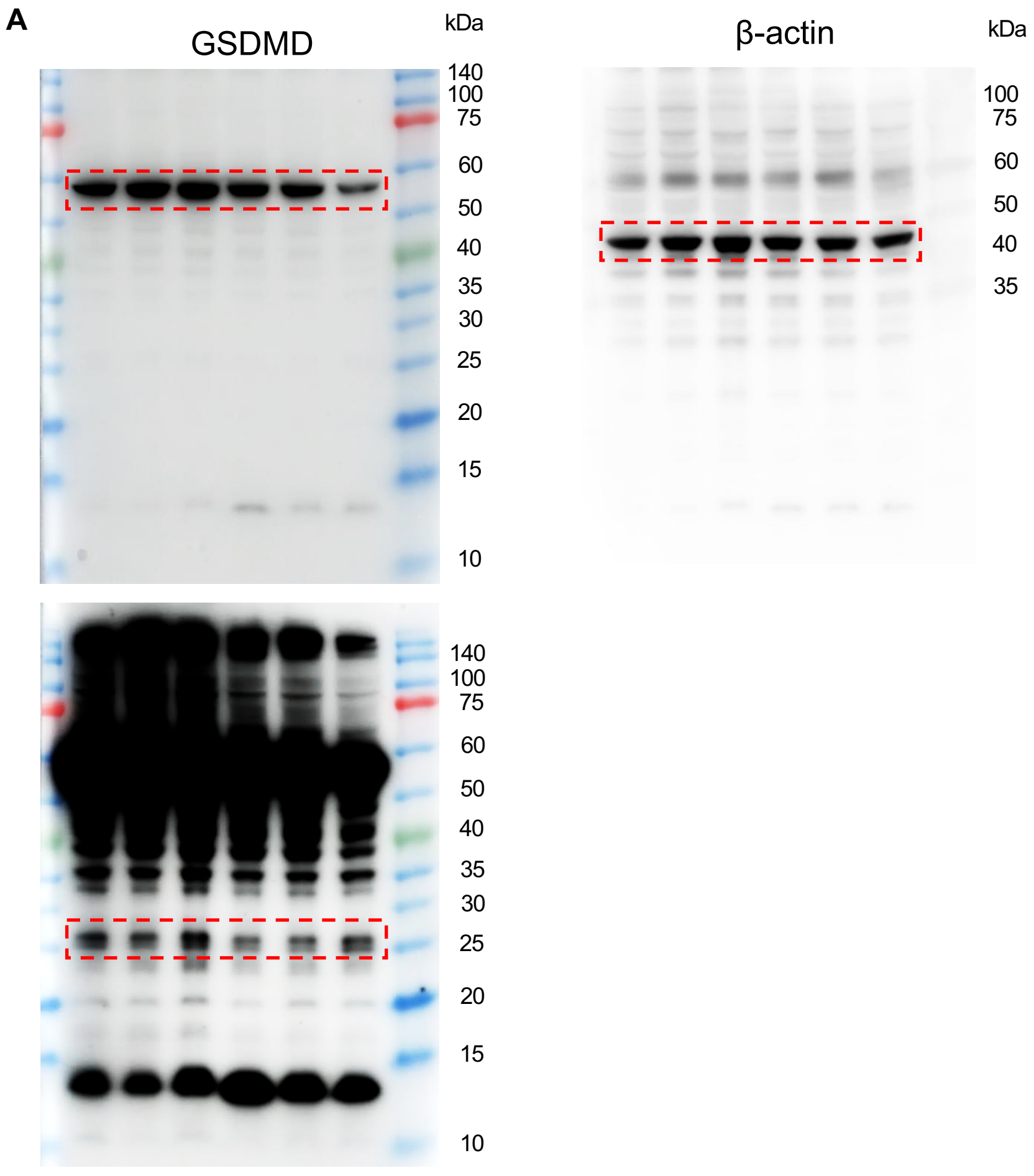
A
GSDMD
kDa
140
100
75
60
50
40
35
30
25
20
15
10
140
100
75
60
50
40
35
30
25
20
15
10
β-actin
kDa
100
75
60
50
40
35

Fig. 6

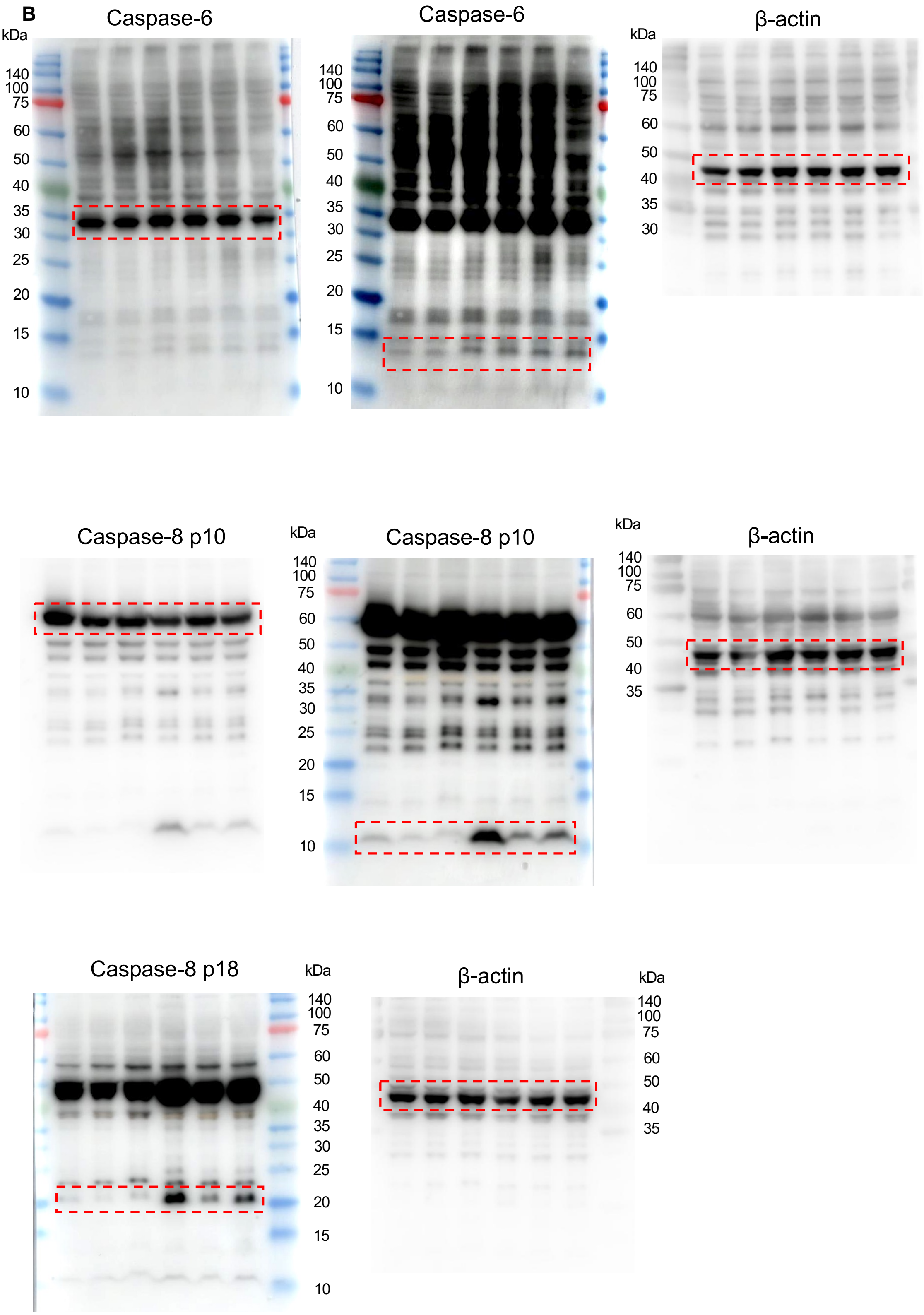
B
Caspase-6
kDa
140
100
75
60
50
40
35
30
25
20
15
10
Caspase-6
β-actin
Caspase-8 p10
Caspase-8 p10
β-actin
Caspase-8 p18
β-actin

Fig. 6

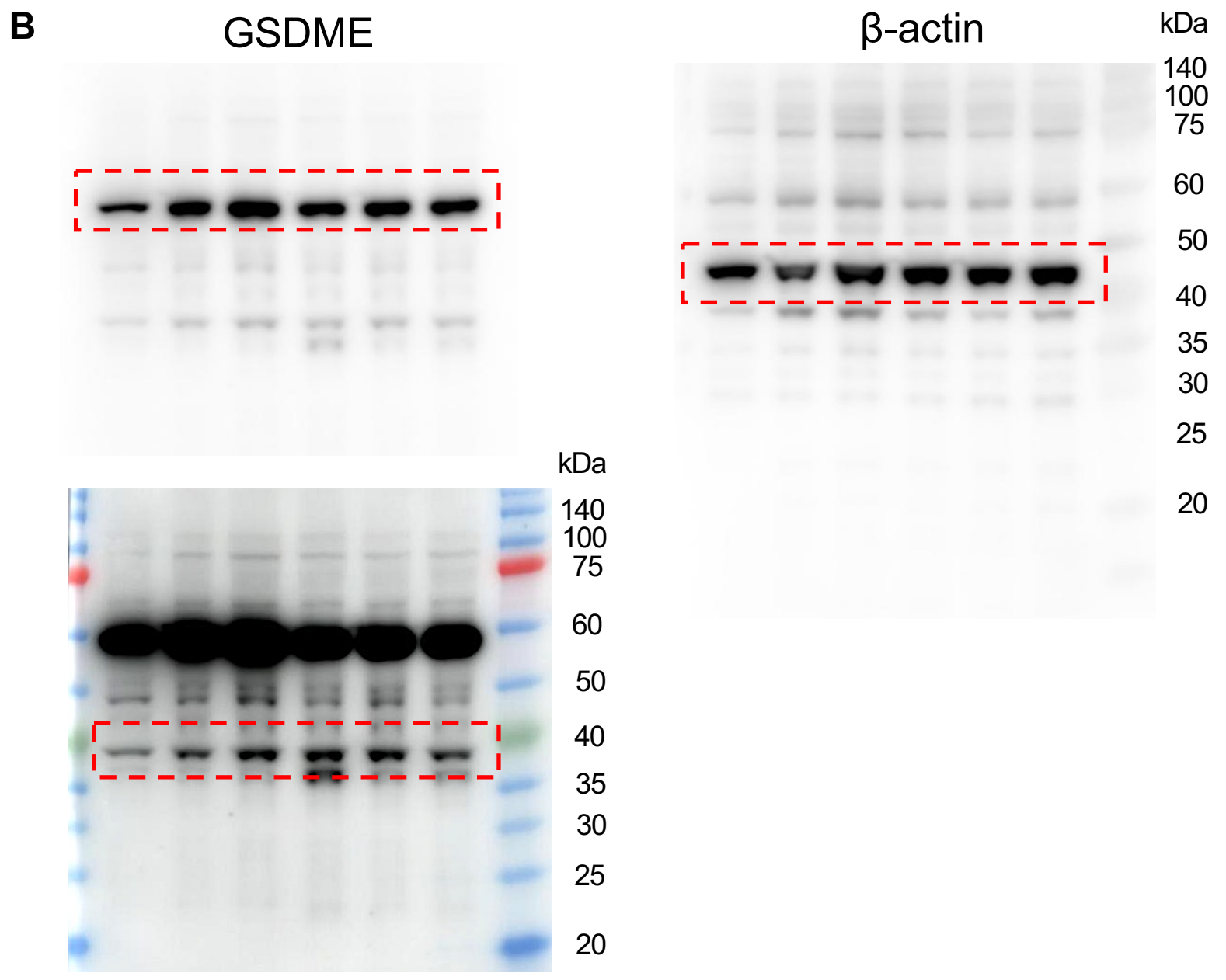
B
GSDME
β-actin
kDa
140
100
75
60
50
40
35
30
25
20
kDa
140
100
75
60
50
40
35
30
25
20

Fig. 6

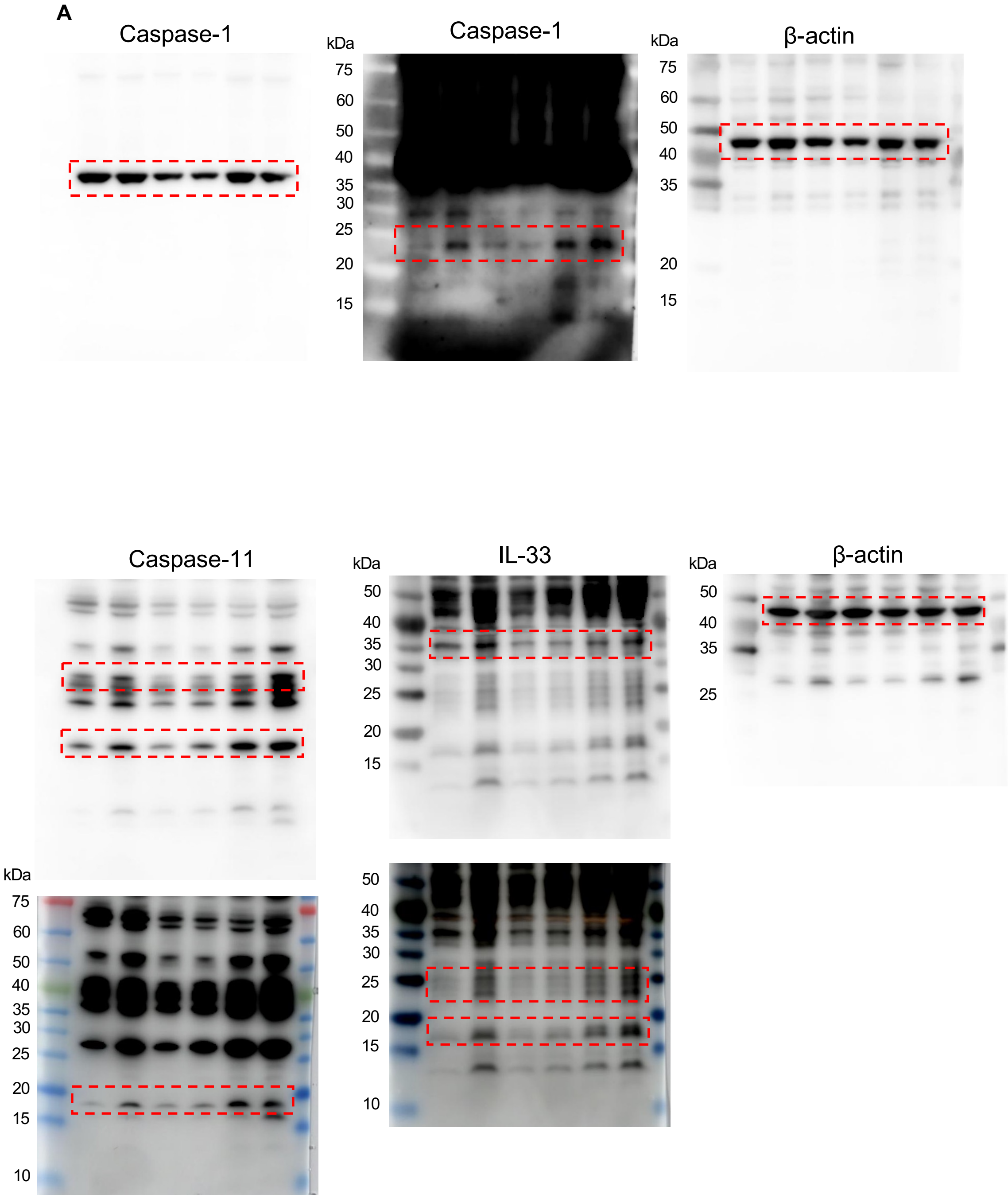
A
Caspase-1
kDa
75
60
50
40
35
30
25
20
15
Caspase-1
kDa
75
60
50
40
35
20
15
β-actin
Caspase-11
kDa
50
40
35
30
25
20
15
IL-33
kDa
50
40
35
25
β-actin
kDa
75
60
50
40
35
30
25
20
15
10
50
40
35
30
25
20
15
10

Fig. 7

A

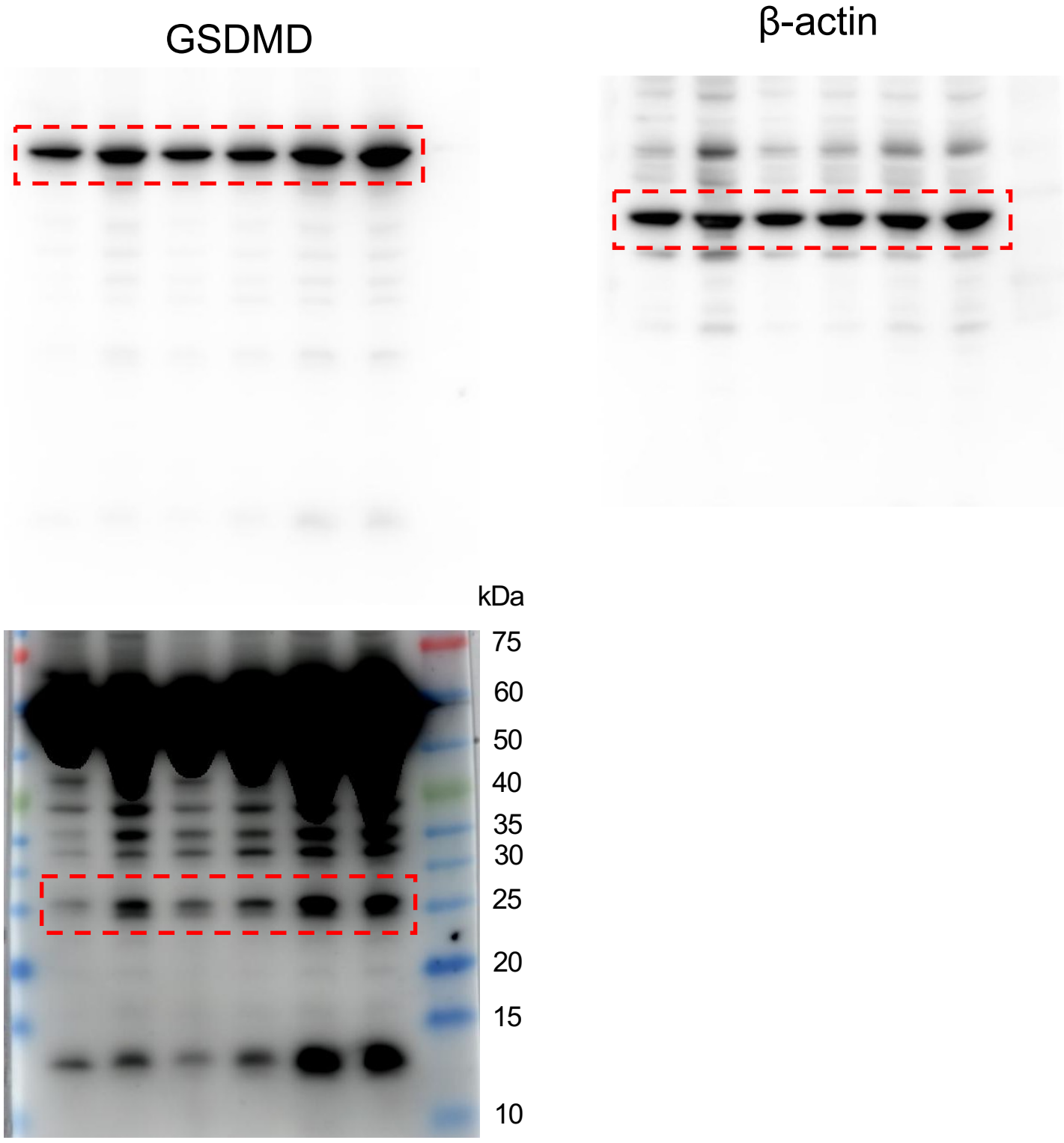

Fig. 7

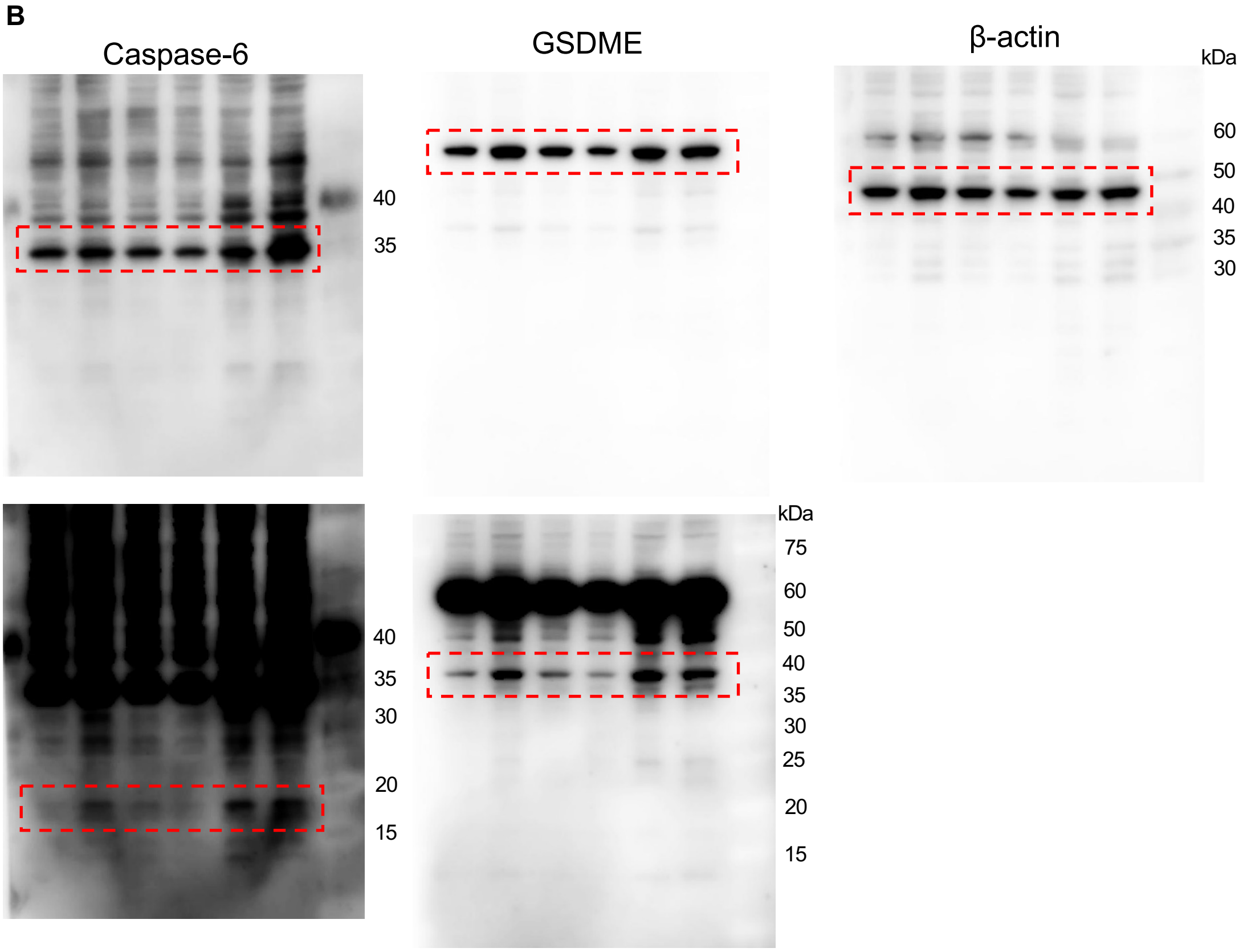
B
Caspase-6
GSDME
β-actin
kDa
60
50
40
35
30
40
35
40
35
30
20
15
kDa
75
60
50
40
35
30
25
20
15

Fig. 7

B

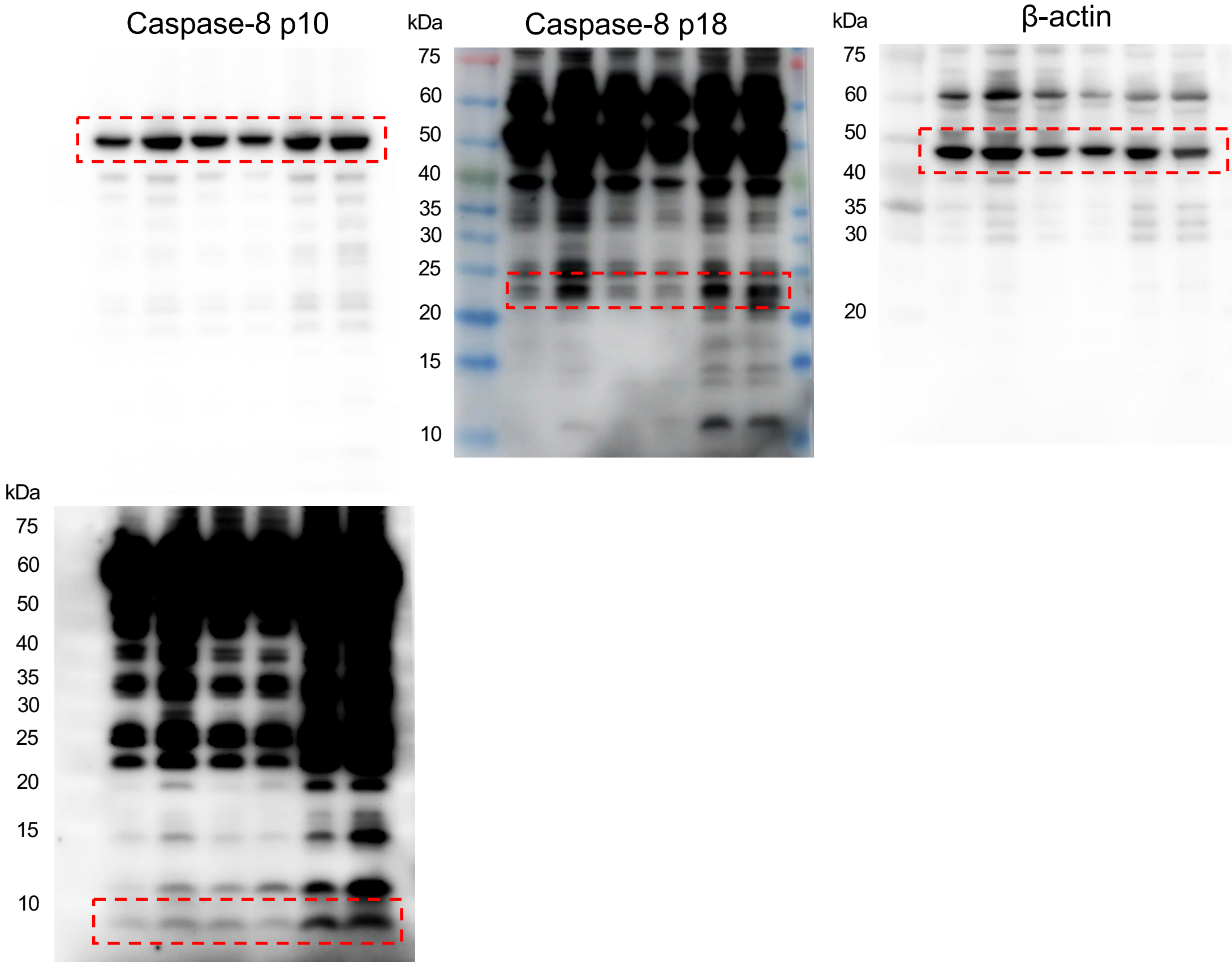
Caspase-8 p10
kDa
75
60
50
40
35
30
25
20
15
10
Caspase-8 p18
kDa
75
60
50
40
35
30
25
20
15
10
β-actin
kDa
75
60
50
40
35
30
20

Fig. 7

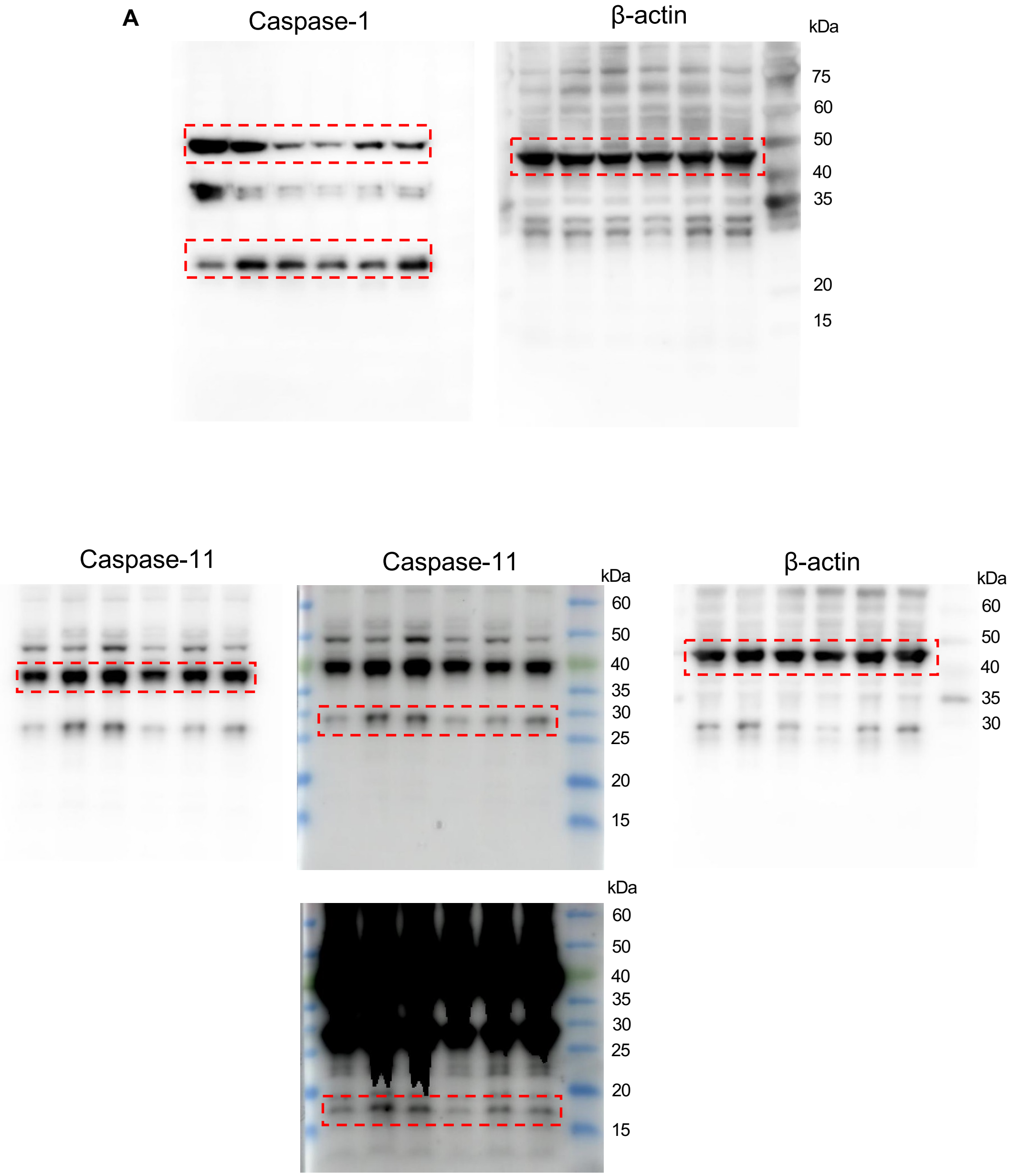
A
Caspase-1
β-actin
kDa
75
60
50
40
35
20
15
Caspase-11
Caspase-11
kDa
60
50
40
35
30
25
20
15
β-actin
kDa
60
50
40
35
30
kDa
60
50
40
35
30
25
20
15

Fig. 8

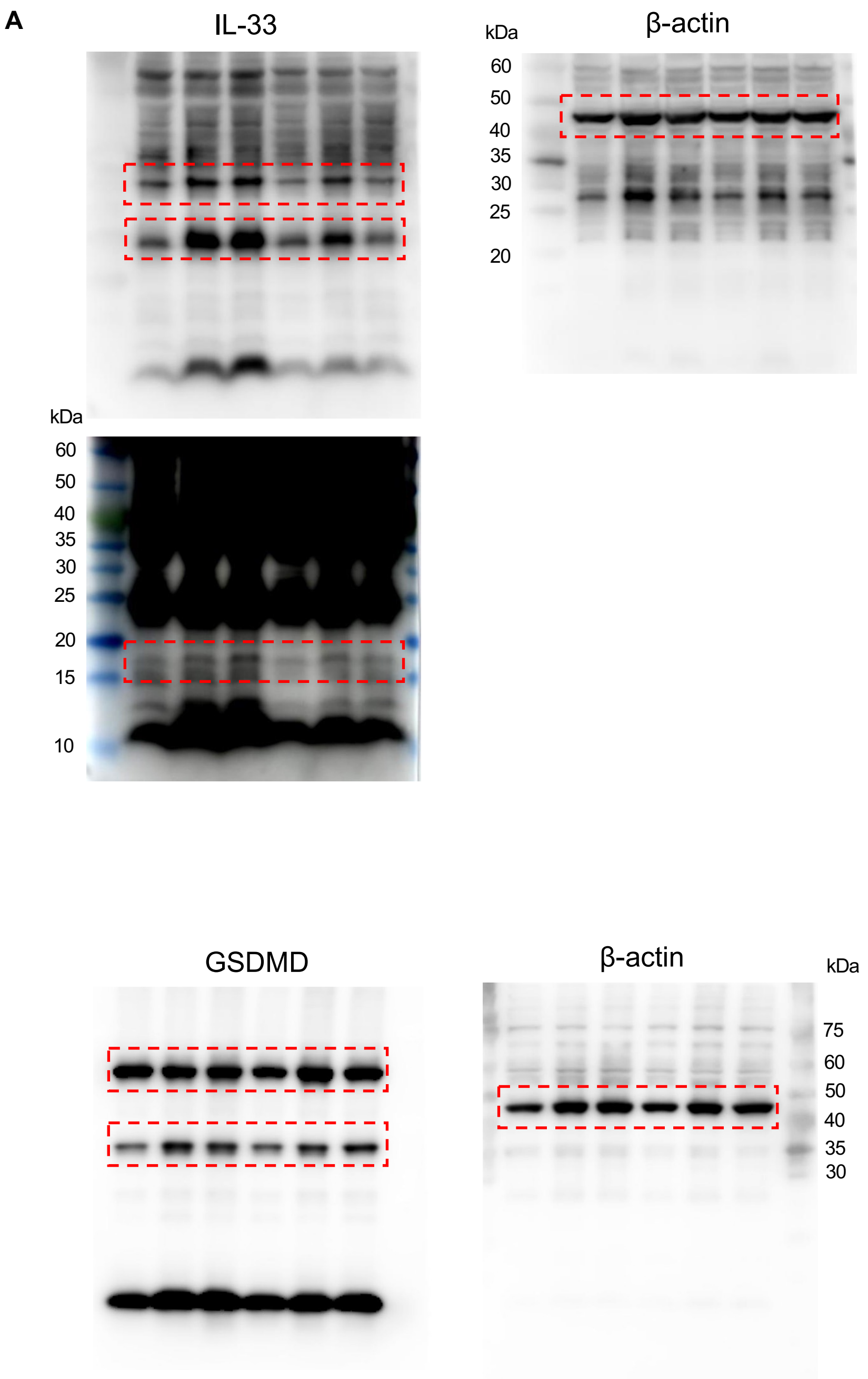
A
IL-33
kDa
60
50
40
35
30
25
20
15
10
β-actin
kDa
60
50
40
35
30
25
20
GSDMD
β-actin
kDa
75
60
50
40
35
30

Fig. 8

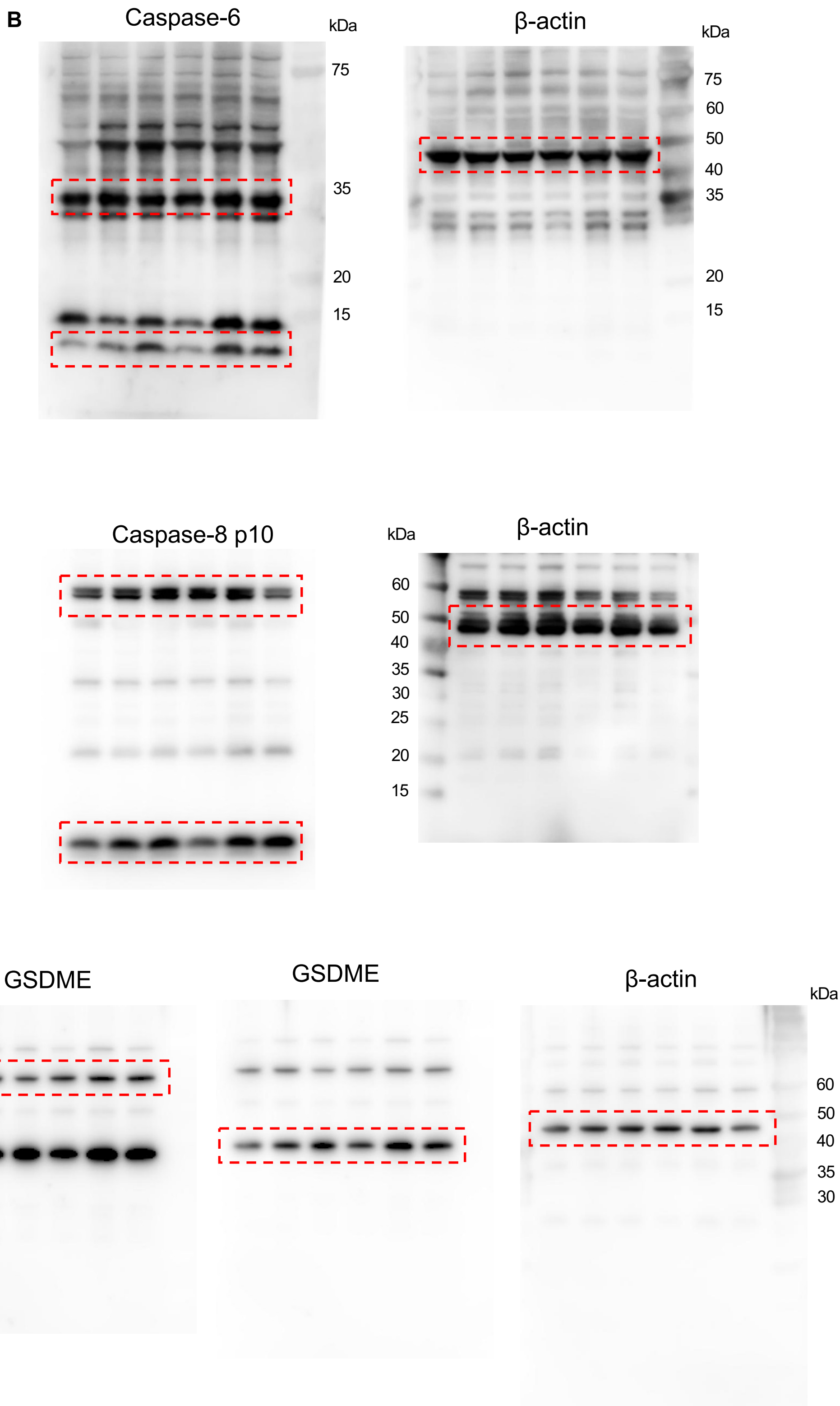
B
Caspase-6
kDa
75
35
20
15
β-actin
kDa
75
60
50
40
35
20
15
Caspase-8 p10
kDa
β-actin
60
50
40
35
30
25
20
15
GSDME
GSDME
β-actin
kDa
60
50
40
35
30

Fig. 8
